# Causal message-passing for experiments with unknown and general network interference

**DOI:** 10.1073/pnas.2322232121

**Published:** 2024-09-27

**Authors:** Sadegh Shirani, Mohsen Bayati

**Affiliations:** ^a^Operations, Information & Technology, Graduate School of Business, Stanford University, Stanford, CA 94305

**Keywords:** experiment design, network interference, total treatment effect, approximate message-passing

## Abstract

Randomized experiments are a powerful methodology for data-driven evaluation of decisions or interventions. Yet, their validity may be undermined by network interference. This occurs when the treatment of one unit impacts not only its outcome but also that of connected units, biasing traditional treatment effect estimations. Our study introduces a framework to accommodate complex and unknown network interference, moving beyond specialized models in the existing literature. Our framework, termed causal message-passing, is grounded in high-dimensional approximate message-passing methodology. It is tailored for multiperiod experiments and is particularly effective in settings with many units and prevalent network interference. The framework models causal effects as a dynamic process where a treated unit’s impact propagates through the network via neighboring units until equilibrium is reached. This approach allows us to approximate the dynamics of potential outcomes over time, enabling the extraction of valuable information before treatment effects reach equilibrium. Utilizing causal message-passing, we introduce a practical algorithm to estimate the total treatment effect, defined as the impact observed when all units are treated compared to the scenario where no unit receives treatment. We demonstrate the effectiveness of this approach across five numerical scenarios, each characterized by a distinct interference structure.

## Significance

Randomized experiments are the gold standard for assessing the impact of various decisions, including policy changes, medical treatments, or product enhancements. However, these experiments often face the challenge of network interference, where a decision affecting one individual impacts others, complicating the analysis. Our research introduces Causal Message-Passing, a framework inspired by techniques from physics and statistics to tackle this complexity. It allows for a more nuanced understanding of interconnected data, crucial for predicting outcomes in environments where individual responses are interdependent. This approach enhances the accuracy of decision-making in networked settings, offering insights for scientists and practitioners across diverse fields.

Randomized experiments are crucial for establishing causal relationships by randomly assigning units (experiment subjects) to treatment and control groups and comparing outcomes. Their effectiveness relies on the stable unit treatment value assumption (SUTVA) (1, 2), which assumes a unit’s outcome is unaffected by other units’ treatments. However, SUTVA often fails in real-world contexts with unit interactions (3). For instance, in studies of medication for contagious diseases, interactions between units can lead to network effects, where treated units influence control units, potentially biasing results. Addressing such network interference is vital for accurate estimation of treatment effect.

When SUTVA is violated, new methods are needed to measure causal effects with minimal assumptions. Arbitrary interference models make estimation infeasible due to nonidentifiability and exponential growth in cardinality of potential outcomes (3–9). Estimation results are also sensitive to model misspecifications (7). Therefore, it is important to relax assumptions and ensure tractable estimation to effectively study causal inference under network interference.

The present study introduces a framework for modeling and analyzing causal effects in the presence of network interference. Specifically, when a unit is treated, the intervention impacts outcomes of its neighboring units. These changes, in turn, impact the outcomes of units interacting with those neighbors, creating a dynamic process that propagates through the network of unit interactions until equilibrium is reached. Drawing inspiration from statistical physics (10, 11), this dynamics can be seen as the dissemination of information through a network of units via message exchanges. We then employ the approximate message-passing (AMP) methodology (12, 13) to show that the dynamics of potential outcomes over time can be approximated by one-dimensional state evolution equations. In light of this, we refer to our approach as Causal Message-Passing (Causal-MP).

The proposed framework has also a potential outcome interpretation (14). Specifically, at each time instant, the model represents the outcome for each unit as a weighted combination of nonlinear functions applied to the outcomes of units in previous time periods, their treatment assignments, and their covariates. This structure, reminiscent of neural networks, enables the model to adapt to broad families of network interference patterns.

The rigorous theoretical investigation of the proposed model introduces a toolkit for designing and analyzing methods to study unobserved counterfactuals in the presence of time-dependent network interference. To the best of our knowledge, no existing research addresses time-dependent network interference. However, further assumptions, particularly regarding identifiability, are necessary to estimate causal effects. We illustrate this in scenarios where the interference pattern has a time-invariant mean and in multiperiod Bernoulli randomized designs under a nonlinear parametric assumption on the interference structure. This leads to a straightforward algorithm with strong consistency guarantees for estimating the total treatment effect (TTE), which we validate through extensive simulations. Notably, our estimation procedure operates without any prior knowledge of the interference structure. We defer the adaptation of this algorithm to settings with more systematic time-dependent interference, using our time-dependent state evolution results, to future studies.

Unlike mean-field models in the network interference literature, which typically assume that each unit interacts with a collective average field, Causal-MP focuses on units and their unique interactions within a network, accounting for heterogeneous unit behaviors, and enabling analysis of treatment effects before they reach equilibrium.

## Example and Challenges in Network Interference.

**Fig. 1.**
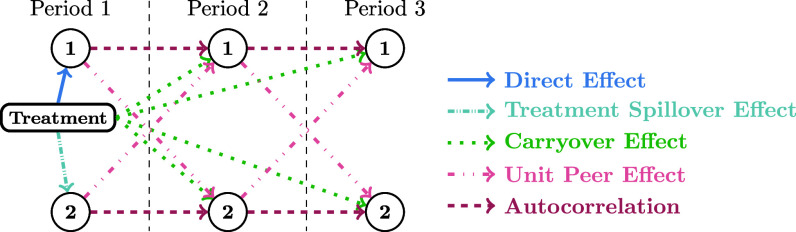
Illustration of different causal relationships; circles represent unit outcomes.

Assume we aim to evaluate a new medical treatment for a contagious disease by observing two individuals over three time periods (Fig. 1). The treatment triggers several effects: a direct effect on the treated unit (unit 1), a treatment spillover effect impacting the control unit (unit 2), and a carryover effect influencing future outcomes (4, 15, 16). Additionally, interactions between units lead to unit peer effects and autocorrelation, adding complexity (5, 17). An anticipation effect, where units’ behavior is influenced by expected future events, also exists but is not shown in Fig. 1.

A causal model must encompass these diverse effects while tackling several challenges. Identifying causal relationships is complex due to intricate interactions, especially when interference structures are unknown. Interference patterns evolve, requiring models that account for temporal changes. Treatment efficacy may also change over time, demanding models that span multiple periods. Additionally, budgetary constraints limit the treatment group size, necessitating methods that provide reliable inferences even with fewer treated units. Noise and unobserved covariates further complicate the analysis, requiring models that can handle incomplete data effectively.

Our proposed framework aims to address these complexities by simplifying evolving high-dimensional complexities into tractable one-dimensional dynamics at any point in time. This approach opens the door to utilizing all observed data during the experiments, even before the effects stabilize.

## 1. Other Related Literature

The assumption of no interference, known as SUTVA, is foundational in causal inference (1, 2, 9, 18). However, recent research increasingly aims to relax this assumption. Some studies focus on testing for interference (15, 19–24), while others propose new assumptions and methods for estimating causal effects without SUTVA (5, 25–33). We survey these emerging developments and proceed by a brief discussion of the literature on AMP algorithms.

### 1.1. Neighborhood Interference Assumption (NIA).

The NIA is a prominent approach in the literature for relaxing SUTVA, positing that an individual’s outcome is influenced solely by the treatments of neighboring units in the network (9). Studies have expanded NIA under known or partially observed interference patterns (25, 26, 30, 31, 33). In the absence of known network structures, (5, 28, 29) have proposed unbiased estimators under various designs and constraints. Recently, under a more flexible version of NIA, the consistency of inverse-probability weighting estimators has been derived (27). Unlike these studies, this work does not assume NIA.

### 1.2. Partial Interference.

Partial interference is another route for relaxing SUTVA, where the population is partitioned into nonoverlapping clusters without any interference between them (34–36). Extending this concept to complex networks, the bias in standard estimators increases by the number of intercluster edges (5). Examples of approaches to mitigate this bias include variants of cluster-randomized designs (37–39), all necessitate knowledge of the interference network, a prerequisite not essential for the current study.

### 1.3. Restrictions on Interference Network.

In the literature, various constraints have been placed on interference structures beyond the NIA and partial interference. Examples include bounding the largest node degree and proving the asymptotic normality of certain estimators (32, 40), introducing methods based on unique observation patterns or localized interference (30, 41, 42), and restricting network topologies (26, 27, 31, 43). Application-specific restrictions on interference patterns have also been explored (44–49), encompassing contexts ranging from marketplaces to dynamical systems.

### 1.4. Single-time Point Observation.

The majority of the literature on network interference has concentrated on single-time point observations, thereby observing each unit’s outcome once (5, 8, 25, 27, 28, 33, 50–53). Recently, there is a pivot toward multitime point observations (54). In this evolving context, Li and Wager (32) discuss challenges and the utility of additional data, while Boyarsky and Namkoong (55) utilize temporal treatment variations to model interference. This work also studies multiperiod experiments.

### 1.5. Deterministic versus Stochastic Models.

The literature typically considers outcomes and network structures as deterministic, with randomness only in treatment assignments (5, 8, 19, 25, 27, 51, 53, 56). Recent studies, however, have incorporated stochastic elements, using random graph models (33), Bernoulli-distributed outcomes (32), and analyzing network noise impacts on bias and variance (57). This work considers a stochastic model with randomness in outcomes, treatments, covariates, and interference patterns.

### 1.6. Model-Based Approaches.

An alternative approach uses structured outcome models, typically linear, to account for interference (5, 31, 43, 58–63). This method requires prior knowledge of network topology to choose relevant statistics. A key issue is that these models can oversimplify complex social interactions (64). The model in this paper aims to capture more complex patterns of interference.

### 1.7. AMP.

The origins and motivations for AMP trace back to refs. 12, 65, and 66. The theoretical foundation was established in refs. 13 and 67 and further expanded with various degrees of generality by other authors (68–76). For a comprehensive overview, see refs. 77–79. AMP traditionally studies high-dimensional estimation problems via iterative dynamical systems involving nonlinear transformations and a mixing matrix. The goal is to use state evolution to refine the nonlinear functions and improve estimation accuracy. Our approach diverges as we observe the outcomes, not the mixing matrix or nonlinear functions, and use state evolution to infer statistics for estimating causal effects. AMP is related to tree approximation methods from statistical physics (80), information theory (81), and computer science (82), with its insights primarily rooted in statistical physics (65).

## 2. Problem Formulation and Main Results

In this section, we present an outcome specification that captures a general interference structure, followed by an informal description of our theoretical results,^*^ which form the basis for a practical algorithm to estimate causal effects and an algorithm for estimating CI. Finally, we illustrate an application of Causal-MP in the context of the Bernoulli randomized design.

### 2.1. Potential Outcome Specification.

Before introducing the setup, we define the notations [M] and ${\left[M\right]}_{0}$ to refer to the sets 1,…,M and 0,1,…,M, respectively, for any positive integer M. Now, consider a setting with N units indexed by n in [N] over a time horizon of T+1 periods, indexed by t in ${\left[T\right]}_{0}$. At time instant t>0, each unit n is randomly assigned a treatment denoted by $W_{t}^{n}$ which is distributed according to a probability distribution $\pi_{t}$. We refer to the set $E=\{\pi_{1},\ldots ,\pi_{T}\}$ as the experimental design (9, 83) and assume that for all t, the support of $\pi_{t}$ is a subset of the real line that includes 0 and at least one nonzero element. Whenever $W_{t}^{n}=0$, we say that unit n is under control; otherwise, we say unit n receives the treatment. We let $W\in {\;R}^{T\times N}$ be the treatment assignment matrix, where its $n^{\mathrm{th}}$ column ${\vec{W}}^{n}$ represents the treatment assignment of unit n throughout the experiment: ${\vec{W}}^{n}=(W_{1}^{n},\ldots ,W_{T}^{n}{)}^{⊤}$. Additionally, w denotes a specific realization of W.

We adopt the Neyman–Rubin causal framework (14) and denote by $Y_{t}^{n}\left(W\right)$ the potential outcome of unit n at time t. In this context, the observed data consist of a treatment allocation w and outcomes $Y_{t}^{n}\left(W=w\right)$, $n\in \left[N\right],\;t\in {\left[T\right]}_{0}$. In addition, for an integer M, we let the matrix $X\in {\;R}^{M\times N}$ be the covariate matrix such that its $n^{\mathrm{th}}$ column (denoted by ${\vec{X}}^{n}$) gives the characteristics of unit n (e.g., age, gender).

To define the potential outcome specification, we let ${\left\{g_{t}\right\}}_{t\in {\left[T\right]}_{0}}$ be a family of unknown measurable functions such that $g_{t}:\;R\times {\;R}^{T}\times {\;R}^{M}\mapsto \;R$. For each t and n, the output from $g_{t}$ is given as $g_{t}\left(Y_{t}^{n}\left(W\right),{\vec{W}}^{n},{\vec{X}}^{n}\right)$. With a slight extension in notation, $g_{t}({\vec{Y}}_{t}\left(W\right),W,X)$ represents a vector of size N, where the $n^{\mathrm{th}}$ element is defined by $g_{t}\left(Y_{t}^{n}\left(W\right),{\vec{W}}^{n},{\vec{X}}^{n}\right)$. Here, we denote ${\vec{Y}}_{t}\left(W\right)=(Y_{t}^{1}\left(W\right),\ldots ,Y_{t}^{N}\left(W\right){)}^{⊤}$ which is the column vector that contains the outcomes of all individuals at time t. Then, given the vector of initial outcomes ${\vec{Y}}_{0}$, for t=0,1,…,T−1, we define

[1] $$\begin{array}{c} {\vec{Y}}_{t+1}\left(W\right)=(A+B_{t})g_{t}\left({\vec{Y}}_{t}\left(W\right),W,X\right)+{\vec{\epsilon }}_{t}, \end{array}$$

where A and $B_{t}$ are N×N matrices and ${\vec{\epsilon }}_{t}=(\epsilon_{t}^{1},\ldots ,\epsilon_{t}^{N}{)}^{⊤}$. Here, the matrices A and $B_{t}$ are unknown and capture the interference structure. Let ${\;\text{A}}^{\mathrm{ij}}$ and ${\;\text{B}}_{t}^{\mathrm{ij}}$ denote the element in the $i^{\mathrm{th}}$ row and $j^{\mathrm{th}}$ column of A and $B_{t}$, respectively; ${\;\text{A}}^{\mathrm{ij}}+{\;\text{B}}_{t}^{\mathrm{ij}}$ quantifies the impact of unit j on unit i at time t. We refer to A and $B_{t}$ as the fixed interference matrix and time-dependent interference matrix, respectively. Let $G_{t}=A+B_{t}$, the interference matrix at time t. The function $g_{t}$ represents the impact of past outcomes, treatment assignments, and covariates on current outcomes. Finally, ${\vec{\epsilon }}_{t}$ is the zero-mean noise term accounting for misspecifications and measurement errors. We eliminate the notation W in ${\vec{Y}}_{t}\left(W\right)$ and simply write $Y_{t}$ whenever there is no ambiguity in the potential outcome concept.

Remark 1. [Direct Effect] In Eq. **1**, the direct effect of the treatment of each unit i on its outcome, $Y_{t+1}^{i}$, results from the combination of impact of $W_{t+1}^{i}$ on the function $g_{t}$ followed by multiplication by ${\;\text{A}}^{\mathrm{ii}}+{\;\text{B}}_{t}^{\mathrm{ii}}$. In *SI Appendix*, section 6.1, we discuss a setting involving a more explicit direct effect.

Remark 2. [Nonadditive Noise] In *SI Appendix*, section 6.2, we consider a more general setting where a nonlinear random operator is applied to Eq. **1**, allowing for a broader range of outcome distributions, including binary outcomes.

One can interpret the specification in Eq. **1** as follows: If we exclude the noise term ${\vec{\epsilon }}_{t}$, the potential outcome for unit n at time t+1 is a weighted combination of “messages” it receives from all other units. Each message is a (nonlinear) function of the outcome of the sending unit at time t, its entire treatment assignment, and its covariate vector. This is expressed as $Y_{t+1}^{n}=\sum_{i\in [N]}{\;\text{G}}_{t}^{\mathrm{ni}}g_{t}\left(Y_{t}^{i},{\vec{W}}^{i},{\vec{X}}^{i}\right)$. The potential outcome $Y_{t+1}^{n}$ will subsequently be used in the message that unit n sends to other units in future periods. Tracing back in time and interpreting each $Y_{t}^{i}$ as a weighted combination of the messages it receives from other units at time t, the impact of unit i on unit n is shaped by a combination of the impacts it receives from other units, combined with the treatment assignment and personal characteristics of unit i. This message exchange captures dynamics of the interference effect within the network of units. A more detailed discussion of this message-passing interpretation, including the absence of the well-known Onsager term present in AMP formulations, is deferred to *SI Appendix*, section 9.

The specification in Eq. **1** captures key interference effects depicted in Fig. 1, such as treatment spillover, unit peer effects, and autocorrelation. Furthermore, the treatments and outcomes of all units can affect the outcome of any specific unit, thus relaxing the commonly employed NIA in the literature (9). Additionally, Eq. **1** accounts for the propagation of intervention effects across the network, enabling distant units to influence each other’s outcomes through intermediary units over multiple time periods. The presence of each unit’s entire treatment assignment over time on the right-hand side of Eq. **1** also represents the anticipation effect.

In the remaining, we assume the interference matrices, as well as the noise vectors to be Gaussian random variables with unknown parameters.

Assumption 1 *Entries of* A *are, i.i.d., Gaussian random variables with mean* μ/N *and variance* $\sigma^{2}/N$, *independent from anything in the model. Similarly, for any* $t\in {\left[T\right]}_{0}$, *entries of* $B_{t}$ *are, i.i.d., Gaussian random variables with mean* $\mu_{t}/N$ *and variance* $\sigma_{t}^{2}/N$, *independent from all other sources of the randomness in the model*.

Assumption 2 *Elements of noise vector* ${\vec{\epsilon }}_{t}$ *are, i.i.d., Gaussian random variables with mean zero and finite variance* $\sigma_{e}^{2}$, *independent from anything in the model*.

We note that Assumption 1 simplifies the theoretical analysis and supports rich interference patterns observed in real data. In *SI Appendix*, section 6.4, we show that our results extend to settings where entries of A and $B_{t}$ are binary under neighborhood or partial interference assumptions. Leveraging insights from the universality results from the AMP literature, we discuss how our results are expected to hold when these entries are not necessarily, i.i.d., or Gaussian. Moreover, numerical results in section 4 validate our theoretical predictions in settings with binary entries under the NIA, across various networks such as Facebook friends data, random geometric graphs, Erdös-Rényi graphs, and clustered networks. To provide some intuition for the richness of Assumption 1, we note that the SD of the entries of A and $B_{t}$ are assumed to be of order $O(1/\sqrt{N})$, while their means are of order O(1/N). This allows the entries to be heterogeneous, even though they share a common mean and distribution. Such heterogeneity permits diverse local dependencies between outcomes and treatments that cannot be captured with a mean-field assumption requiring outcomes to be independent, conditional on a global average quantity.

In the following sections, we will rigorously analyze data generated by Eq. **1** under Assumptions 1 and 2. Our main objective is to develop practical algorithms for identifying and estimating the underlying causal effects.

### 2.2. Causal Estimands.

The literature has explored several estimands for causal effects, with a focus on average effects rather than individual-level effects (5, 23, 27, 32, 53, 84). Here, we aim to estimate the total treatment effect (TTE), also known as global treatment effect (GTE). This estimand measures the average effect of altering the treatment for the entire community. For instance, in the case of a contagious disease, TTE quantifies the reduction in the number of infections or the impact on healthcare costs whenever the new medication is administered to all individuals, as compared to a scenario where nobody receives it. Precisely, for any two treatment allocations $w^{\prime }$ and $w^{\prime \prime }$, we define

[2] $$\begin{array}{c} \;{\text{TTE}}_{t}\left(w^{\prime \prime },w^{\prime }\right)=\lim_{N\to \infty }\frac{1}{N}\sum_{n=1}^{N}(Y_{t}^{n}\left(w^{\prime \prime }\right)-Y_{t}^{n}\left(w^{\prime }\right))\;. \end{array}$$

A notable special case arises when entries of $w^{\prime \prime }$ and $w^{\prime }$ are drawn from Bernoulli distributions with means $\pi^{\prime \prime }$ and $\pi^{\prime }$, respectively (32, 37). Then, with a slight abuse of notation, we write $\;{\text{TTE}}_{t}\left(\pi^{\prime \prime },\pi^{\prime }\right)$. For example, the case with $\pi^{\prime \prime }=1$ and $\pi^{\prime }=0$ represent scenarios in which all treatment assignments are set to 1 or 0, respectively. In problems where only a single observation of the data is accessible, the literature typically assumes that t is sufficiently large for the effect to have stabilized and then estimates $\;{\text{TTE}}_{t}\left(1,0\right)$ at the equilibrium (5, 9, 27, 53). However, in this paper, we aim to estimate $\;{\text{TTE}}_{t}\left(\overset{ˇ}{\pi },0\right)$ for any desired $\overset{ˇ}{\pi }\in \left[0,1\right]$ and all values of t, which will allow us to trace the evolution of $\;{\text{TTE}}_{t}\left(\overset{ˇ}{\pi },0\right)$, even before stabilization. $\;{\text{TTE}}_{t}\left(\overset{ˇ}{\pi },0\right)$ is particularly relevant when it is impractical to deliver the treatment to the entire population. In general, estimating the TTE is insightful in scenarios where a decision maker aims to use the result of the experiment to determine whether the treatment should be expanded to everyone or not (37).

#### 2.2.1. Direct and indirect effects.

TTE is generally a combination of indirect (network) and direct effects. In *SI Appendix*, section 6.1, we also discuss how to estimate these two components.

### 2.3. State Evolution of the Experiment.

Let the potential outcomes ${\left\{Y_{t}^{n}\right\}}_{n\in \left[N\right],t\in {\left[T\right]}_{0}}$ follow Eq. **1**. The goal is to derive efficient estimators for the TTE defined in Eq. **2**. To this end, we will show that

[3] $$\begin{array}{c} \lim_{N\to \infty }\sum_{n=1}^{N}\frac{Y_{t}^{n}}{N}\;\overset{\;\text{a.s.}}{=}\nu_{t}\left(E\right),\;\lim_{N\to \infty }\sum_{n=1}^{N}\frac{{\left(Y_{t}^{n}\right)}^{2}}{N}-\nu_{t}{\left(E\right)}^{2}\;\overset{\;\text{a.s.}}{=}\rho_{t}{\left(E\right)}^{2}, \end{array}$$

where deterministic quantities $\nu_{t}\left(E\right)$ and $\rho_{t}{\left(E\right)}^{2}$ are defined by the recursions,

[4] $$\begin{array}{c} \begin{array}{cc} \nu_{t+1}\left(E\right) & =\left(\mu +\mu_{t}\right)E\left[g_{t}(\nu_{t}\left(E\right)+\rho_{t}\left(E\right)Z,\vec{W},\vec{X})\right],\\ \rho_{t+1}{\left(E\right)}^{2} & =\left(\sigma^{2}+\sigma_{t}^{2}\right)E\left[g_{t}(\nu_{t}\left(E\right)+\rho_{t}\left(E\right)Z,\vec{W},\vec{X}{)}^{2}\right]+\sigma_{e}^{2}\;, \end{array} \end{array}$$

that are initialized by $\nu_{1}\left(E\right)$ and $\rho_{1}{\left(E\right)}^{2}$, representing the expected sample mean and variance of outcomes at t=1, respectively. In addition, Z∼N(0,1) is independent from $\left(\vec{W},\vec{X}\right)∼\Pi \times p_{X}$, where $\Pi :=\pi_{1}\times \ldots \times \pi_{T}$ and $p_{X}$ denotes the (large N) limit of the empirical distribution of columns of the covariate matrix X. Thus, $p_{X}$ defines a probability distribution over $R^{M}$ and Π denotes the probability distribution of the treatments determined by E.

Note that Eq. **4** allows tracking the dynamics of $\nu_{t}\left(E\right)$ and $\rho_{t}{\left(E\right)}^{2}$, which represent the large N sample mean and variance of the potential outcomes at time t, respectively. To simplify the notation, we drop the reference to E and simply write $\nu_{t}$ and $\rho_{t}^{2}$, whenever there is no ambiguity.

We also show a more general result than Eq. **4**, under some moment conditions concerning $p_{X}$ and Π. For any continuous^†^ function ψ with at most polynomial growth, we show:

[5] $$\begin{array}{c} \lim_{N\to \infty }\sum_{n=1}^{N}\frac{\psi (Y_{t}^{n},{\vec{W}}^{n},{\vec{X}}^{n})}{N}\overset{\;\text{a.s.}}{=}\;E\left[\psi (\nu_{t}+\rho_{t}Z,\vec{W},\vec{X})\right]\;. \end{array}$$

Then, inspired by the literature on AMP algorithms, we refer to Eq. **4** as state evolution equations of the experiment. We provide the rigorous statements and related details in section 3. Here, we discuss the intuition and implications derived from Eqs. **4** and **5**.

Consider an experimental data ${\left\{Y_{t}^{n}\left(w\right)\right\}}_{n\in \left[N\right],t\in {\left[T\right]}_{0}}$ from a given experimental design E. Note that we can utilize these data to estimate the limiting sample means ${\left\{\nu_{t}\right\}}_{t\in {\left[T\right]}_{0}}$ and the sample SD ${\left\{\rho_{t}\right\}}_{t\in {\left[T\right]}_{0}}$ using Eq. **3**. These statistics serve as the basis for estimating complex functions involving units’ outcomes, treatment assignments, and covariates using Eq. **5**. Having access to E and $p_{X}$, the unknown parameters in the state evolution equations are denoted as $U:=\left(\mu ,\sigma ,{\left\{\mu_{t}\right\}}_{t\in {\left[T\right]}_{0}},{\left\{\sigma_{t}\right\}}_{t\in {\left[T\right]}_{0}},{\left\{g_{t}\right\}}_{t\in {\left[T\right]}_{0}},\sigma_{e}\right)$. By estimating U, we can estimate any desired average counterfactual, including the TTE defined in Eq. **2**.

Note that our approach does not impose any constraints on the experimental design and does not require specific knowledge about interference structure. As a result, the state evolution equations, besides Eq. **5**, offer a versatile framework for the causal analysis of data. Notably, Eq. **5** suggests that we can effectively compute various functions, relevant to understanding the average behavior of individuals.

Overall, the framework presented in this section addresses the challenges of analyzing high-dimensional interconnected data by reducing it to the study of one-dimensional state evolution equations. However, the problem of estimating the unknown parameters U still needs to be tackled.

### 2.4. Causal Effects Estimation.

In this section, we propose a meta algorithm for estimating causal effects, building on Eqs. **4** and **5**. This is done by estimating the parameters $U:=\left(\mu ,\sigma ,{\left\{\mu_{t}\right\}}_{t\in {\left[T\right]}_{0}},{\left\{\sigma_{t}\right\}}_{t\in {\left[T\right]}_{0}},{\left\{g_{t}\right\}}_{t\in {\left[T\right]}_{0}},\sigma_{e}\right)$ using the available data. We then utilize Eqs. **4** and **5** for a second time to compute the desired counterfactuals and estimands. In this regard, according to the available data and our specific research objectives, we can introduce constraints on the parameters of the outcome model Eq. **1** to facilitate the estimation procedure of U. Algorithm 1outlines a general scheme to estimate the total treatment effect when altering the treatment allocations from $w^{\prime }$ to $w^{\prime \prime }$, corresponding to experimental designs $E^{\prime }$ and $E^{\prime \prime }$, respectively.

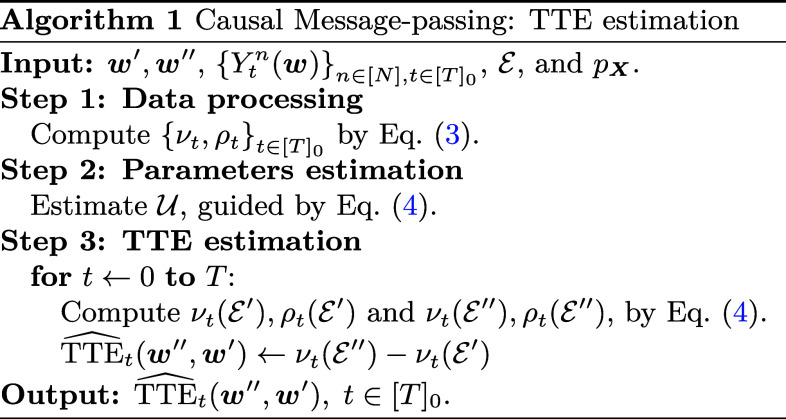

#### Explanation of Algorithm 1.

Algorithm 1presents a systematic and flexible framework for conducting the estimation process, which comprises three main steps explained below.

##### Step 1: Data processing.

The initial step of the algorithm focuses on data preprocessing, transforming the data into a suitable format for further analysis. The computational cost of this step scales linearly with the number of units and results in the generation of two vectors of size T+1.

##### Step 2: Parameters estimation.

In the second step, we utilize the preprocessed data to estimate U. To accomplish this, we can make use of a parametric function class for ${\left\{g_{t}\right\}}_{t\in {\left[T\right]}_{0}}$, and introduce simplifying assumptions about the interference matrices. By leveraging the observed outcomes and Eq. **4**, we estimate the relevant parameters.

Note that we can consider more complex models by collecting richer data. For example, if we can divide units into distinct clusters or conduct the experiment in multiple stages with different treatments, we can incorporate additional data into the estimation. This allows using more sophisticated outcome models that capture the intricacies of the data.

##### Step 3: TTE estimation.

In the final step, the algorithm estimates the TTE by estimating the counterfactual scenarios under experimental designs $E^{\prime }$ and $E^{\prime \prime }$. The algorithm then computes the TTE over the desired time horizon. It is important to note that the time horizon for estimating the TTE can differ from the time horizon of collecting the data.

Overall, Algorithm 1is designed to estimate the total treatment effect by observing a specific scenario and estimating other counterfactual scenarios. It should be noted that the designs $E_{1}$ and $E_{2}$, and therefore $w^{\prime }$ and $w^{\prime \prime }$, are arbitrary and at least one of the counterfactual scenarios cannot be directly observed. However, by leveraging the available data and estimation techniques, the algorithm provides an estimate of the TTE. It is worth mentioning that the same procedure can be adapted to estimate other estimands related to the system. In the next sections, we discuss a heuristic for the estimation of the CI of TTE and then we give a more concrete version of Algorithm 1tailored to the context of Bernoulli randomized design.

### 2.5. CI.

To guarantee the reliability of our TTE estimates, obtaining CI is important. While we defer a proper treatment of this topic to follow-up studies, we describe a heuristic approach here that seems promising, based on the numerical studies in section 4. By utilizing the data ${\left\{Y_{t}^{n}\left(w\right)\right\}}_{n\in \left[N\right],t\in {\left[T\right]}_{0}}$, we employ the state evolution equations (Eq. **4**) to devise a resampling-based heuristic for CI estimation of the TTE. This method involves resampling unit outcomes and repeatedly using Algorithm 1to approximate the TTE distribution, considering the outcome dependencies due to network interference.

Specifically, CI computation involves a two-step method. For a given q∈(0,1) and positive integer B, the first step is resampling the units B times, with each unit’s inclusion relying on an independent Bernoulli random variable with probability q. Then, for each sample, Algorithm 1is applied to estimate the TTE over the time horizon, yielding B values of TTE estimates for each time t. The second step involves calculating the CI using the mean and SD of these estimated TTEs across the B samples. Selecting q should balance a trade-off between the accuracy of each sample’s estimates, which increases with higher q, against the correlation between samples, which favors lower q values. While a thorough analysis of this method is reserved for future work, our numerical results in section 4 suggest that selecting smaller q as N grows provides reasonable CI.

### 2.6. Application to Bernoulli Randomized Design.

We consider a two-stage Bernoulli randomized experiment as a specific case of the experimental design. This approach aligns with the prevailing practice in many firms, where a dynamic phase release of the new treatment is carried out through a sequence of randomized experiments (24, 85). Subsequently, the time horizon is divided into two intervals: $\left\{0,1,\ldots ,T_{1}\right\}$ and $\left\{T_{1}+1,\ldots ,T=T_{1}+T_{2}\right\}$. Each unit receives the treatment with probabilities $\pi_{1}$ and $\pi_{2}$ in the first and second intervals, respectively, where $\pi_{1}\neq \pi_{2}$. The main objective is to estimate the $\;{\text{TTE}}_{t}\left(\overset{ˇ}{\pi },0\right)$, as defined in section 2.2, throughout the time horizon ${\left[T\right]}_{0}=\left\{0,1,\ldots ,T\right\}$. Here, letting $\pi_{1}=0$ is equivalent to considering historical data with no experiment in the first stage.

Additionally, we consider approximation of the function $g_{t}$, described as follows:

[6] $$\begin{array}{c} \begin{array}{cc} g_{t}\left(Y_{t}^{n},{\vec{W}}^{n},{\vec{X}}^{n}\right) & =\Delta^{n}+\Xi^{n}Y_{t}^{n}+\Lambda^{n}W_{t+1}^{n}\\ & \;\;\;+\Gamma^{n}Y_{t}^{n}W_{t+1}^{n}+{\left({\vec{\Theta }}^{n}\right)}^{⊤}{\vec{X}}^{n}. \end{array} \end{array}$$

In Eq. **6**, $\Delta^{n},\Xi^{n},\Lambda^{n},\Gamma^{n}\in \;R$ and ${\vec{\Theta }}^{n}\in {\;R}^{M}$ are unknown random objects independent of everything else. Eq. **6** is a first-order approximation of the function $g_{t}$ plus the second-order term $\Gamma^{n}Y_{t}^{n}\;W_{t+1}^{n}$. In this context, $\Delta^{n}$ represents the baseline effect, the coefficients $\Xi^{n},\Lambda^{n},\Gamma^{n}$ correspond to the specific effects of the current outcome and treatment, and the random vector $\Theta^{n}$ captures the influence of the covariates of the unit n. Note that all these coefficients are treated as random objects, specific to each unit. This differs from the existing literature, where both the outcomes and network structure are assumed to be deterministic (8, 15, 19, 51).

Considering the contagious disease example in Figure 1, the expression $\Delta^{n}+\Xi^{n}Y_{t}^{n}+{\left({\vec{\Theta }}^{n}\right)}^{⊤}{\vec{X}}^{n}$ represents the severity of symptoms in the absence of medication. It combines several components: $\Delta^{n}$ captures the inherent severity level, $\Xi^{n}Y_{t}^{n}$ reflects the influence of the current health condition on future health outcomes, and ${\left({\vec{\Theta }}^{n}\right)}^{⊤}{\vec{X}}^{n}$ incorporates the impact of the individual specific covariates such as age, gender, etc., on their health condition. The term $\Lambda^{n}W_{t+1}^{n}+\Gamma^{n}Y_{t}^{n}W_{t+1}^{n}$ accounts for the effect of administering the new medication to individual n. The inclusion of the multiplicative term $\Gamma^{n}Y_{t}^{n}W_{t+1}^{n}$ allows the consideration that the efficacy of treatment can vary according to the severity of symptoms. This flexible modeling approach recognizes the potential heterogeneity in treatment effects, allowing for a more nuanced understanding of the impact of the new medication.

We restrict ourselves to the setting where $\mu_{t}$ is constant. To simplify the notation, without loss of generality, we modify the coefficients $\Delta^{n}$, $\Xi^{n}$, $\Lambda^{n}$, $\Gamma^{n}$, and ${\vec{\Theta }}^{n}$, assuming $\mu_{t}+\mu =1$. This implies that while the interference pattern can vary over time, i.e., the matrix $B_{t}$ can change completely over time, its mean remains the same throughout the experiment. In the context of our example, this means that individuals may interact with different intensities on different days, but the overall interaction level remains constant over time. We will show in the numerical simulations of *SI Appendix*, section 7.4 that the above model is robust even when the data contain seasonal trends (i.e., when $\mu_{t}$ varies). Meanwhile, we anticipate that one approach to handling the case of varying $\mu_{t}$, similar to our parametric assumption on $g_{t}$, would be to assume a parametric form for $\mu_{t}$ by expressing it as a linear combination of basis functions of t, which we defer to future work.

Algorithm 2outlines the procedure for estimating the TTE in the specified context. It employs data segmented into two parts of lengths $T_{1}$ and $T_{2}$, with treatment probabilities $\pi_{1}$ and $\pi_{2}$, respectively. Similar to Algorithm 1, it begins with a preprocessing step, followed by two linear regressions to estimate the necessary parameters. The algorithm then uses the sample mean of observed outcomes to estimate the counterfactual scenario for the desired treatment level $\overset{ˇ}{\pi }$ and calculates the TTE in the final step. The consistency of this estimator is demonstrated in section 3.

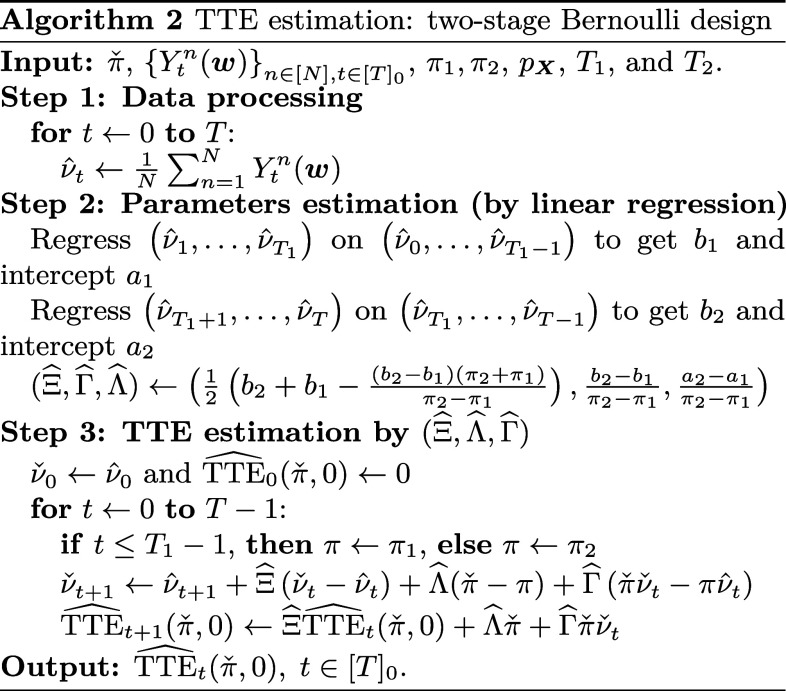

Note that Algorithm 2operates without requiring covariate estimation. This feature is particularly advantageous in scenarios with unobserved covariates. By bypassing the need for covariate inclusion, Algorithm 2streamlines the analysis, reducing potential biases and complexities associated with covariate observation or estimation.

While we impose certain restrictions on the structure of the functions $g_{t}$ in Eq. **6**, note that these restrictions still encompass a wide range of linear and nonlinear models that have been extensively studied in the existing literature (5, 9, 31, 32). In addition, in section 4, we provide comprehensive numerical analysis to support the flexibility and adaptability of Algorithm 2. The data generation processes in these examples do not obey the specifications assumed by Algorithm 2and are designed to assess the applicability of the algorithm in estimating the TTE, even when the data are generated using more complex underlying structures.

Remark 3. [Incorporating Prior Information] In Algorithm 2, performance may improve by integrating prior information relevant to the data and setting at hand. For instance, during the update ${\hat{\;\text{TTE}}}_{t+1}\left(\overset{ˇ}{\pi },0\right)\leftarrow \hat{\Xi }{\hat{\;\text{TTE}}}_{t}\left(\overset{ˇ}{\pi },0\right)+\hat{\Lambda }\overset{ˇ}{\pi }+\hat{\Gamma }\overset{ˇ}{\pi }{\hat{\nu }}_{t}\left(\overset{ˇ}{\pi }\right)$, one might project the right-hand side to specific subintervals of R, informed by prior knowledge.

Remark 4. [TTE Estimation at Equilibrium] We can also use state evolution equations Eq. **4** to establish an estimator for the TTE at equilibrium. This stands in contrast to Algorithm 2, which is tailored to estimate the TTE over the entire time horizon. The formal statement of this finding is laid out in *SI Appendix*, section 10.

## 3. Technical Results and Proofs Overview

In this section, we first outline and discuss the technical assumptions required for the proofs. We then present the rigorous statements of the theoretical contributions. We conclude by analyzing Algorithm 2and assessing the consistency of the resulting estimator.

### 3.1. Main Result.

We proceed by considering a sequence of systems indexed by N, representing the population size. Accordingly, we use ${\vec{Y}}_{t}^{\left(N\right)}$, $W^{\left(N\right)}$, and $X^{\left(N\right)}$ to denote the vector of potential outcomes at time t, treatments, and covariates of the $N^{\mathrm{th}}$ system. We drop the notation N when there is no ambiguity. Then, for any fixed t>0, we analyze the behavior of the elements in ${\vec{Y}}_{t}^{\left(N\right)}$ as the system size N approaches infinity. This investigation provides us with valuable insights into the evolution of the outcomes and how they are influenced by the design of the experiment.

To present the main results, we introduce several notations. For any vector $\vec{v}\in {\;R}^{\ell }$, for some ℓ≥1, we denote its Euclidean norm as $∥\vec{v}∥$. For a fixed k≥1, we define CP(k) as the class of functions $f:{\;R}^{\ell }\to \;R$, that are continuous and exhibit polynomial growth of order k. That is, there exists a constant c such that $\left|f\left(\vec{x}\right)\right|\leq c\left(1+{\left\|\vec{x}\right\|}^{k}\right)$. Moreover, we consider a probability space (Ω,F,P), where Ω represents the sample space, F is the sigma-algebra of events, and P is the probability measure. We denote the expectation with respect to P as E. Additionally, for any other probability measure p, we use $E_{p}$ to denote the expectation with respect to p.

Next, we state an assumption that is standard in the AMP literature and then discuss it in the context of our experimental design problem.

Assumption 3 *Fix* k≥2. *We assume that*

1. *For any* $t\in {\left[T\right]}_{0}$, *the function* $g_{t}:{\;R}^{1+T+M}\to \;R$ *is a* $CP(\frac{k}{2})$ *function*.
2. *Let* $p_{N}$ *be the empirical distribution of columns of* X, *then* $p_{N}$ *converges weakly to a probability measure* $p_{X}$ *on* ${\;R}^{M}$ *such that* $E_{p_{X}}[{\left\|\vec{X}\right\|}^{k}]<\infty$ *and, as* N *grows to* ∞, $E_{p_{N}}[{\left\|\vec{X}\right\|}^{k}]\to E_{p_{X}}[{\left\|\vec{X}\right\|}^{k}]$.
3. *If* $\vec{W}∼\Pi$, *then* $E[{\left\|\vec{W}\right\|}^{k}]<\infty$.
4. ${\vec{Y}}_{0}$, W, X, *and the function* $g_{0}$ *are such that for some deterministic values* $\nu_{1}$ *and* $\rho_{1}$, *we have*
  $$\begin{array}{cc} \nu_{1} & =\lim_{N\to \infty }\frac{\mu +\mu_{0}}{N}\sum_{n=1}^{N}g_{0}(Y_{0}^{n},{\vec{W}}^{n},{\vec{X}}^{n})<\infty \;,\\ 0<\rho_{1}^{2}-\sigma_{e}^{2} & =\lim_{N\to \infty }\frac{\sigma^{2}+\sigma_{0}^{2}}{N}\sum_{n=1}^{N}g_{0}(Y_{0}^{n},{\vec{W}}^{n},{\vec{X}}^{n}{)}^{2}<\infty \;. \end{array}$$
5. *There exist a* $CP(\frac{k}{2})$ *function* ${\overset{¯}{g}}_{0}:{\;R}^{T+M}\mapsto \;R$ *such that for all* t *and* $CP(\frac{k}{2})$ *functions* $\varphi :{\;R}^{T+M}\mapsto \;R$, *we have*
  $$\begin{array}{c} \lim_{N\to \infty }\frac{1}{N}\sum_{n=1}^{N}g_{0}(Y_{0}^{n},{\vec{W}}^{n},{\vec{X}}^{n})\varphi \left({\vec{W}}^{n},{\vec{X}}^{n}\right)\\ \overset{\;\text{a.s.}}{=}\;E\left[{\overset{¯}{g}}_{0}(\vec{W},\vec{X})\varphi \left(\vec{W},\vec{X}\right)\right], \end{array}$$
  *and* $E\left[{\overset{¯}{g}}_{0}{\left(\vec{W},\vec{X}\right)}^{2}\right]\leq \frac{\rho_{1}^{2}-\sigma_{e}^{2}}{\sigma^{2}+\sigma_{0}^{2}}$ *where* $\left(\vec{W},\vec{X}\right)∼\Pi \times p_{X}$.

Assumption 3 encompasses a set of regularity conditions on the system parameters and model attributes. Specifically, Part 1 ensures that the functions $g_{t}$ do not exhibit fast explosive behavior, guaranteeing the well-posedness of the large system asymptotics. Part 2 ensures that the empirical distribution $p_{N}$ remains stable and does not diverge as the sample size increases. This assumption holds, for instance, when unit covariates (the columns of the covariate matrix X) are, i.i.d., with distribution $p_{X}$ with finite moments of order k. Moreover, Assumption 3-3 holds for a wide range of treatment assignments, including cases where the support of Π is bounded, such as the Bernoulli design.

Assumptions 3-4 and 3-5 are required for the proofs and rule out restrictive initial conditions. For example, both are satisfied if $g_{0}$ is a nonzero function and the sequence of initial outcomes ${\vec{Y}}_{0}$ is drawn from a distribution that possesses finite moments of order k. Specifically, a straightforward application of the law of large numbers in Theorem 9, outlined in the appendices, yields 3-4. Subsequently, reusing the same theorem along with 3-4 implies 3-5. Overall, Assumptions 3-4 and 3-5 ensure that the initial outcomes ${\vec{Y}}_{0}$ and the function $g_{0}$ are informative and contribute to the estimation process.

Regularity conditions related to the outcome functions are commonly found in the existing literature. Examples include assuming bounded moments of a certain degree for the potential outcome functions (53), considering bounded outcomes (27), and assuming boundedness of the potential outcome function and its derivatives (33).

Given $\nu_{1}$ and $\rho_{1}$ as in Assumption 3, we proceed by considering the state evolution equations in Eq. **4** for t≥1. Then, we present the following theorem that formalizes a more general version of the result that was previously stated in section 2.3. This theorem also characterizes the joint distribution of $Y_{1}^{n},\ldots ,Y_{t+1}^{n}$ within large sample asymptotics, providing a better understanding of the underlying statistical properties of a high-dimensional network data.

Theorem 1 *Fixing* k≥2, *assume the sequence of initial outcomes* ${\vec{Y}}_{0}$, *the treatment assignment* W, *as well as the covariates* X *are given and suppose Assumption 3 holds. Then, we have the following statements for all* t≥0.

1. *For any function* $\psi :{\;R}^{t+1+T+M}\mapsto \;R$ *that* ψ∈CP(k), *we have*
  [7] $$\begin{array}{c} \begin{array}{cc} & \lim_{N\to \infty }\frac{1}{N}\sum_{n=1}^{N}\psi (Y_{1}^{n},\ldots ,Y_{t+1}^{n},{\vec{W}}^{n},{\vec{X}}^{n})\\ \overset{\;\text{a.s.}}{=}\; & E[\psi (\nu_{1}+\rho_{1}Z_{1},\ldots ,\nu_{t+1}+\rho_{t+1}Z_{t+1},\vec{W},\vec{X})], \end{array} \end{array}$$
  *where* $Z_{s}∼N\left(0,1\right),\;s=1,\ldots ,t+1,$ *are independent of* $\left(\vec{W},\vec{X}\right)∼\Pi \times p_{X}$.
2. *Let* $V_{t}$ *be a matrix with columns equal to* $Y_{s},\;s=1,\ldots ,t$; *that is* $V_{t}:=\left[Y_{1}|Y_{2}|\ldots |Y_{t}\right]$. *Then, the following matrix is positive definite almost surely*:
  [8] $$\begin{array}{c} \lim_{N\to \infty }\frac{V_{t}^{⊤}V_{t}}{N}-\lim_{N\to \infty }\frac{V_{t}^{⊤}1_{N\times 1}}{N}\lim_{N\to \infty }\frac{1_{1\times N}V_{t}}{N}≻0, \end{array}$$
  *where* $1_{l_{1}\times l_{2}}$ *is matrix of size* $l_{1}\times l_{2}$ *with all entries equal to* 1.

Broadly speaking, Theorem 1 yields concise one-dimensional dynamical equations that consolidate the analysis of high-dimensional network data in the large sample asymptotic. Specifically, Eq. **7** indicates that unit outcomes follow a Gaussian distribution at any given time. Moreover, when considering the entire time horizon, the outcomes exhibit a nondegenerate multivariate normal distribution (Eq. **8**). This implies that the joint distribution of outcomes can be well-characterized and analyzed. In summary, Theorem 1 provides a framework for analyzing high-dimensional network data. It establishes the Gaussian nature of outcomes, facilitating the computation of various statistics and functions that capture the average behavior of the system.

To obtain the results of Theorem 1, the main challenge arises from the dependence between the fixed interference matrix A and potential outcomes ${\left\{Y_{t}^{n}\right\}}_{n\in [N]}$ for any time t>0. Indeed, the observed outcomes reveal some information about A that we need to incorporate into any calculation concerning future observations. To overcome this obstacle, we leverage the “conditioning technique” introduced in ref. 67 and developed further in ref. 13. This technique is commonly employed in the literature on AMP algorithms, e.g., refs. 68, 70, 75, and 79. They typically consider a fixed symmetric coefficient matrix or a Wishart matrix, with entries of order $O(1/\sqrt{N})$, and also assume a pseudo-Lipschitz nonlinearity. We adapt the analysis established in ref. 13 to the current setting, assuming that the coefficient matrix is nonsymmetric, dropping the Lipschitz assumption of nonlinearity and consider an additional noise term in each round. As a result, the analysis becomes easier in some sense (as there is no “Onsager” or memory term), yet involving additional randomness structures. We provide the rigorous proofs in *SI Appendix*, section 11.

Recent literature on finite sample analysis of AMP (75, 76) shows that the distribution of unit outcomes at a finite time t converges to a Gaussian distribution at a rate of $\sqrt{\log(N)/N}$ (76). Accordingly, Algorithm 2must grapple with an added $O(\sqrt{1/N})$ noise present in the sample means. To reduce the effect of this noise term, one can either increase the population size N or extend the experiment duration T. In section 4, we demonstrate that Algorithm 2remains effective even with values of N as small as 500 and T around 60. While deriving the precise impact of finite sample error on estimation results is an exciting research direction, it is worth noting that many tech companies conduct online experiments with thousands to millions of units (86).

### 3.2. Consistency of the TTE Estimator in Algorithm 2.

We proceed by demonstrating that Algorithm 2yields a strongly consistent estimator for the total treatment effect defined in Eq. **2**.

Theorem 2 *Suppose that there exist at least two distinct sample means* ${\hat{\nu }}_{t^{\prime }}\neq {\hat{\nu }}_{t^{\prime \prime }}$, *for some* $t^{\prime }\neq t^{\prime \prime }$, *and* $\Delta^{n},\Xi^{n},\Lambda^{n},\Gamma^{n}\in \;R$ *and* ${\vec{\Theta }}^{n}$, n∈[N], *are random objects independent of everything else with bounded* $k^{\mathrm{th}}$ *moments. Let* ${\hat{\;\text{TTE}}}_{t}\left(\overset{ˇ}{\pi },0\right)$ *be the estimator defined in Algorithm 2. Then*, ${\hat{\;\text{TTE}}}_{t}\left(\overset{ˇ}{\pi },0\right)$ *is a strongly consistent estimator for the total treatment effect; that is, for any* $t\in {\left[T\right]}_{0}$, *we have*

[9] $$\begin{array}{c} \lim_{N\to \infty }{\hat{\;\text{TTE}}}_{t}\left(\overset{ˇ}{\pi },0\right)\overset{\;\text{a.s.}}{=}\;\;{\text{TTE}}_{t}\left(\overset{ˇ}{\pi },0\right). \end{array}$$

According to Theorem 2, as the number of individuals N grows large, the estimator converges to the true value of the TTE almost surely.

We can establish a similar consistency result for Algorithm 1as long as the estimation of parameters in the second step remains consistent. A proof scheme analogous to Theorem 2, relying on the results of Theorem 1 and the state evolution dynamics in Eq. **4**, would be sufficient to obtain these results. In summary, by appropriately choosing the model specifications and accurately estimating the set U in the second step of Algorithm 1, we can leverage the proposed framework to design and analyze various desired causal effects in diverse models. This claim is supported in 4 through a comprehensive analysis of different systems with more general interference patterns that relax the assumptions of Theorem 2.

In addition to TTE estimation, *SI Appendix*, section 6.1 details the estimation of direct and indirect effects. Using Theorem 1, we show that the difference-in-means and Horvitz–Thompson estimators are strongly consistent for the direct effect. This is similar to results of refs. 46 and 53 but in our distinct setting, with prevalent and locally heterogeneous interference, as discussed in section 2. This result, combined with Theorem 2, is then used to estimate the indirect effect.

## 4. Numerical Illustrations

In this section, we investigate five experimental scenarios utilizing a Bernoulli design and employ Algorithm 2to estimate the total treatment effect. We also use the resampling idea outlined in section 2.5 to estimate CI. The first scenario involves a linear-in-means model under a staggered roll-out design with three different interference structures. The second scenario considers a binary potential outcome model, and the third scenario focuses on estimating the total effect of speeding up servers in a parallel server system.

The design employed in each case is detailed subsequently. Unless otherwise specified, each experiment begins from a near-equilibrium state with all units in the control condition. This state is obtained by running the data-generating process with all treatment variables set to zero for 10 “burn-in” periods before the experimentation phase begins. Each simulation is replicated 5,000 times, with each replication involving a new network, a new realization of the randomized treatment assignment, and new noise.

In section 4.1.1, we aim to compare Algorithm 2with several benchmarks. Therefore, we use a stochastic block model that includes a clustered interference pattern, enabling the use of clustered randomized experiments. The remaining scenarios are aimed at showcasing the capability of Algorithm 2to estimate the entire dynamics of TTE and its robustness to varying interference structures or outcome dynamics. In all scenarios, we compare the estimates with the ground truth value of the treatment effect, which is feasible because we have access to the data-generating process and can recreate the necessary counterfactual outcomes. Additional numerical results are provided in *SI Appendix*, section 7.

### 4.1. Linear-in-Means Model with Staggered Roll-out Design.

We begin by adapting and replicating the linear-in-means model (27, 43). However, similar to ref. 37, we make slight modifications to the original static model to capture dynamic settings. Specifically, for i∈[N], we define the following dynamic outcome model:

[10] $$\begin{array}{c} \begin{array}{cc} Y_{t+1}^{i} & =\alpha_{1}+\alpha_{2}\frac{\sum_{j=1}^{N}E^{\mathrm{ij}}Y_{t}^{j}}{\sum_{j=1}^{N}E^{\mathrm{ij}}}+\alpha_{3}\frac{\sum_{j=1}^{N}E^{\mathrm{ij}}W_{t+1}^{j}}{\sum_{j=1}^{N}E^{\mathrm{ij}}}\\ & \;+\alpha_{4}W_{t+1}^{i}+\varepsilon_{i}. \end{array} \end{array}$$

In Eq. **10**, the matrix E$={\left[E^{\mathrm{ij}}\right]}_{i,j}$ defines the adjacency matrix of a graph and $\varepsilon_{n}∼N\left(0,1\right)$. Following ref. (27), we set $\left(\alpha_{1},\alpha_{2},\alpha_{3},\alpha_{4}\right)=\left(-1,0.8,1,1\right)$ as parameters. Here, $\alpha_{1}$ signifies the baseline effect, $\alpha_{2}$ indicates the autocorrelation and peer effect, $\alpha_{3}$ reflects the spillover effect, and $\alpha_{4}$ corresponds to the direct effect, as shown in Fig. 1. Additionally, to deliver the treatments, we have employed a staggered roll-out design with $\left(\pi_{1},\pi_{2}\right)=\left(0.2,0.5\right)$. Under this design, each unit that receives treatment in the first stage remains under treatment in the second stage as well. This assumption takes into account practical constraints that might prevent units from switching between control and treatment groups. For example, in the context of a contagious disease, the new treatment can induce long-lasting effects on the treated individuals, making it impractical or ethically challenging to reverse the treatment once it has been applied.

Next, we investigate the robustness of the proposed method against misspecification in the interference structure by considering three different graphs as detailed below.

#### 4.1.1. Linear-in-means model with stochastic block network.

In the first scenario, inspired by Eckles et al. (37), we consider an interference network captured through a directed stochastic block model with 10 blocks, each representing a different cluster. We examine populations with N= 500, 1,000, and 10,000, where each unit is connected to an average of five units within the same cluster and an average of 0.9 units from other clusters.

We benchmark the performance of Algorithm 2as follows. Considering a randomized controlled trial (RCT) where we randomize the treatment over units, when the interference structure is completely unknown, a potential approach is to ignore the interference and rely on simple estimators (37). Accordingly, we consider difference-in-means (DM) and Horvitz–Thompson (HT) estimators, outlined in *SI Appendix*, section 6.1. In this setting, both DM and HT indeed estimate the direct effect, as shown in *SI Appendix*, section 6.1. However, if the network structure is partially known up to clusters, the literature suggests randomizing the treatments over the clusters, referred to as Cluster RCT (87). In Cluster RCTs, and in the ideal case where clusters have no intercluster connections, both DM and HT are expected to yield unbiased estimates of the TTE (8, 37, 38).

**Fig. 2.**
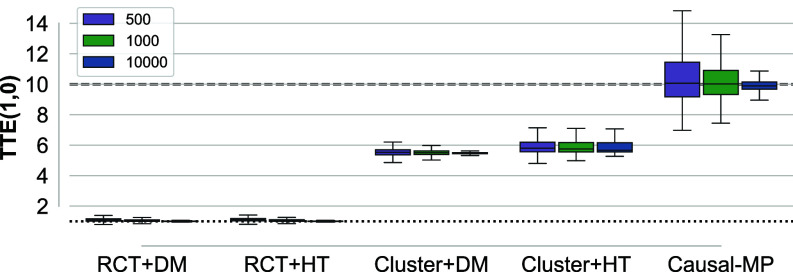
Linear-in-means model with stochastic block network; Causal-MP vs. DM and HT estimators in RCT and Cluster RCT. The ground truth value for the TTE is 10 (the dashed line) and for the direct effect is 1 (the dotted line).

Fig. 2 depicts the results, highlighting the accuracy of Causal-MP compared to other benchmarks, which exhibit significant biases. Specifically, in the absence of perfect network observation, other estimators might overlook unit connections (whether within clusters or between clusters), resulting in biased estimations of the treatment effect. In contrast, applying Algorithm 2without any knowledge of the interference structure provides an accurate estimation of the TTE, underscoring the relevance of the proposed framework.

#### 4.1.2. Linear-in-means model with Facebook friends lists network.

**Fig. 3.**
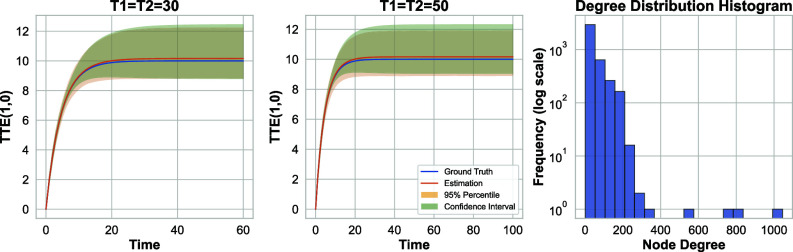
*Left*: Linear-in-means model with Facebook network; 95% CI for the total treatment effect estimation when $\left(\pi_{1},\pi_{2}\right)=\left(0.2,0.5\right)$. *Right*: degree distribution of the Facebook network.

Next, we explore the linear-in-means model as described in Eq. **10**, but utilize a Facebook network dataset with 4,039 nodes and 88,234 edges, obtained from ref. 88. Then, Fig. 3 displays the results. The right plot in this figure illustrates the degree distribution of the nodes, highlighting significant variation in node degrees. Additionally, Fig. 3 includes the (estimated) 95% CI for the output of Algorithm 2, calculated via the heuristic detailed in section 2.5 using B=500 and q=0.15. It is worth noting that the slight bias observed is potentially due to the relatively small network size (N= 4,039) or misspecifications between the assumptions of our proposed methodology and the real network data. But the overall performance demonstrates the versatility of the proposed approach.

#### 4.1.3. Linear-in-means model with random geometric graph.

In this setting, proposed by Leung (27), we generate the graph with adjacency matrix E using random geometric graph models. Specifically, we create a graph with vertex set [N], such that for each pair of distinct units i and j in [N], we define $E^{\mathrm{ij}}=1_{\|{\vec{V}}_{i}-{\vec{V}}_{j}\|\leq r_{N}}$, where for each unit n in [N], ${\vec{V}}_{n}={\left[V_{n}^{1},V_{n}^{2}\right]}^{⊤}\in {\;R}^{2}$ is determined by independently sampling each coordinate from the uniform distribution over the interval [0,1], and $r_{N}=\sqrt{8{\left(\pi N\right)}^{-1}}$. Importantly, this model implies an average connectivity where each individual is linked to 8 other units, which implies a moderate level of interference. Moreover, we let $\varepsilon_{n}=V_{n}^{1}-0.5+N\left(0,1\right)$ in Eq. **10**; consequently, as Leung (27) notes, the noise term generates unobserved homophily, and units with closer $V_{n}^{1}$ values have similar outcomes.

**Fig. 4.**
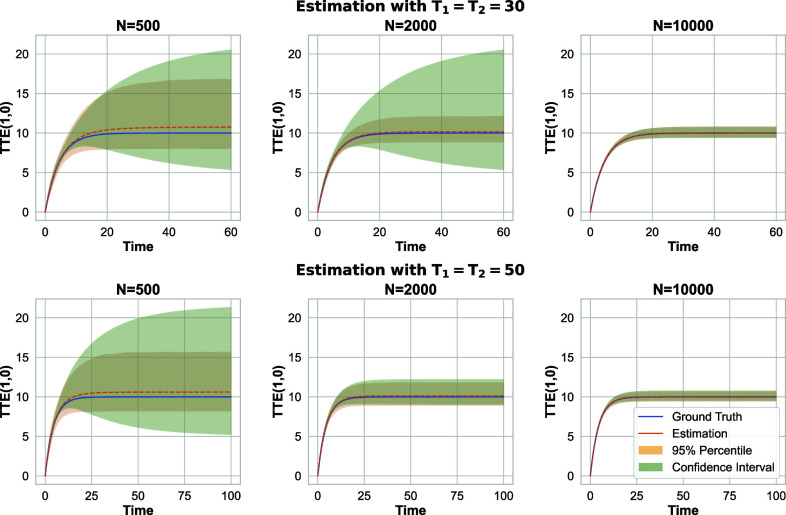
Linear-in-means model with random geometric graph; 95% CI for the total treatment effect estimation when $\left(\pi_{1},\pi_{2}\right)=\left(0.2,0.5\right)$.

Fig. 4 displays the results, while the first row depicts experiments with T=60 and the second row illustrates longer experiments with T=100. This figure presents both the average estimated TTE, obtained using Algorithm 2along with its (true) 95% CI, and the ground-truth TTE derived from the replications. Additionally, Fig. 4 includes the (estimated) 95% CI for the output of Algorithm 2, calculated via the heuristic detailed in section 2.5 using B=500. The parameter q is set to 0.4, 0.3, and 0.25 corresponding to N=500, 2,000, and 10,000, respectively. Finally, in light of Remark 3, we incorporate a “prior-knowledge” that the magnitude of the TTE is at most 100.

As N or T grow in Fig. 4, we observe more accurate estimation. The former directly aligns with the consistency result in Theorem 2. The latter is also (but indirectly) related to Theorem 2. Particularly, the inputs to Algorithm 2contain error terms, that go to zero with N. For any finite N, their impact on the estimation error is compensated with increasing T. This indicates that as we gather more data over an extended period, the accuracy of the estimates further improves, as reflected in the lower width of the CI in the figure.

### 4.2. Binary Outcome Model with Microrandomized Trial.

The setting we examine next is a binary potential outcome model (32). Precisely, we consider $Y_{t+1}^{i}∼\;\text{Bernoulli}\left(O_{t}\right)$, such that,

[11] $$\begin{array}{c} O_{t}=\alpha_{1}+\alpha_{2}W_{t+1}^{i}Z_{t}^{i}+\alpha_{3}Y_{t}^{i}Z_{t}^{i}+\alpha_{4}W_{t+1}^{i}Y_{t}^{i}Z_{t}^{i} \end{array}$$

where $Z_{t}^{i}=\sum_{j=1}^{N}E^{\mathrm{ij}}Y_{t}^{j}$ represents the number of neighbors of individual i with an outcome of 1. Following Example 1 in ref. 32, we let E to be the adjacency matrix of an Erdös-Rényi graph where each pairs of vertices are connected, independently, with probability $p_{\;\text{edge}}$. We set the parameter values as $\left(\alpha_{1},\alpha_{2},\alpha_{3},\alpha_{4},p_{\;\text{edge}}\right)=\left(0.5,0.15,0.20,0.01,3/N\right)$. Consequently, on average, each individual is connected to three other units, resulting in a low interference level in the network.

We adopt a microrandomized trial (MRT) with a Bernoulli design to determine the treatment group in each period. Specifically, we set $\left(\pi_{1},\pi_{2}\right)=\left(0.25,0.75\right)$, and for each period in stage j∈{1,2}, we generate a new treatment vector ${\vec{W}}_{t}$ such that $W_{t}^{i}\;\overset{\;\text{iid}}{∼}\;\;\text{Bernoulli}\left(\pi_{j}\right)$, where $t\in [T_{j}]$. MRTs were initially introduced as an experimental design for developing just-in-time adaptive interventions by Liao et al. (89) and Klasnja et al. (90). Since then, they have gained popularity in various research areas, particularly in studying mobile health interventions aimed at increasing physical activity among sedentary individuals (91).

Here, we consider the binary outcome model without the initial “burn-in” periods and defer the case with the burn-in periods to *SI Appendix*, section 7.2. Indeed, not all experiments begin with the population under study in a stable equilibrium. For instance, consider the evaluation of a new medication for a recently emerged contagious disease. In this situation, the health status of experimental units might be subject to rapid changes due to the disease’s spread and the dynamic interactions among individuals. This instability complicates the estimation of treatment effects because baseline conditions are not consistent. Therefore, it is crucial to develop methods capable of accurately tracking the dynamics of the treatment.

**Fig. 5.**
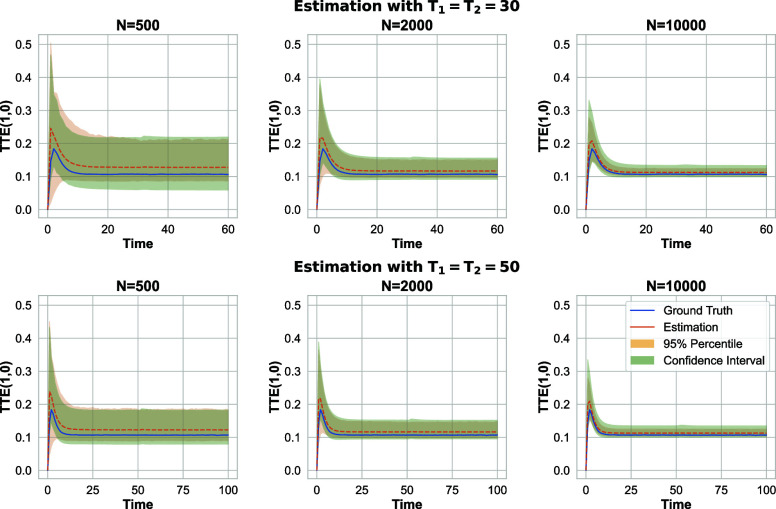
Nonequilibrium binary outcome model: 95% CI for the total treatment effect estimation when $\left(\pi_{1},\pi_{2}\right)=\left(0.25,0.75\right)$.

Fig. 5 shows the results, highlighting a quick jump to the ground truth value of the TTE in the early stages of the experiment. This jump is successfully captured by Algorithm 2, demonstrating its reliable performance in tracking the dynamics of the TTE throughout the experiment. Similar to Fig. 4, increasing the sample size and extending the time horizon leads to significantly improved precision in the estimates. Here, B=500 and q is set to 0.6, 0.5, and 0.3 corresponding to N=500, 2,000, and 10,000, respectively. Given the binary outcome model, we incorporate the prior knowledge that the TTE’s magnitude is capped at 1, as discussed in Remark 3.

### 4.3. Parallel Server System Speed-up Effect.

We next consider a parallel server system with N servers that operates under the join-the-shortest queue policy, a widely adopted routing strategy for server farms (92). This policy assigns each incoming job to one of the shortest queues. Motivated by Kuang and Mendelson (93), our primary focus is on understanding the interference impact of change in the service rate (speeding up the servers in our case) on the overall “server utilization.” To study this, we conduct a randomized experiment by speeding up selected servers. However, it is crucial to account for the interference effect among servers, which arises due to the join-the-shortest queue policy. This interference can cause servers in the control group, maintaining their original service rates, to experience reduced demand as a result of the allocation policy. Consequently, the treatment assignment impacts the control group.

We generate the data by simulating a parallel server system characterized by a Poisson arrival process with a rate of 0.95N and an exponential service time, where each server has a service rate of 1. The treatment we consider involves doubling the speed of randomly selected servers. At the beginning of stage 1 (t=1), we increase the service rate of each server to 2 with a probability $\pi_{1}=0.15$, continuing this setting until the end of the first stage ($t=T_{1}$). Subsequently, at the beginning of stage 2 ($t=T_{1}+1$), we increase the service rate of each server to 2 with a probability $\pi_{2}=0.5$. Therefore, the treatment status of each server remains unchanged within each stage. Then, the observed data from server n at time period t (i.e., the outcome $Y_{t}^{n}$) represents the total time that server n is busy during the time interval [t,t+1). This setup allows us to evaluate the performance of Algorithm 2in a setting with implicit interference introduced by the join-the-shortest queue policy, versus the more explicit specifications as in Eqs. **10** and **11**.

**Fig. 6.**
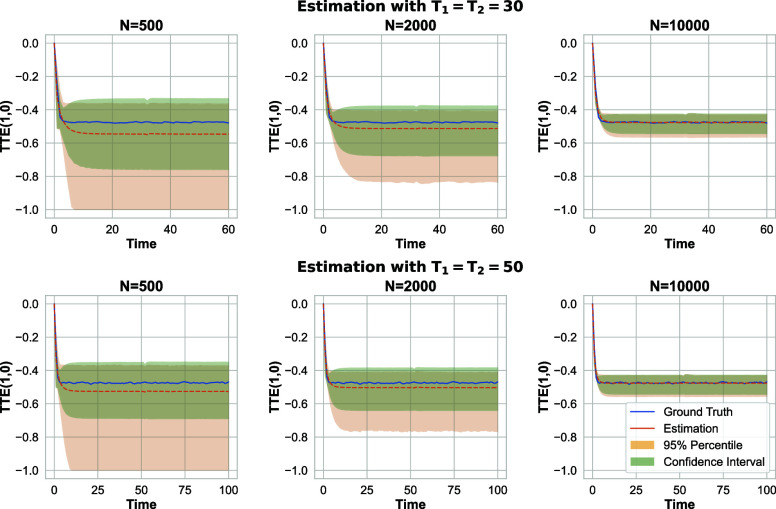
Server speed-up problem with implicit interference due to the join-the-shortest queue policy; 95% CI for the total treatment effect estimation when $\left(\pi_{1},\pi_{2}\right)=\left(0.15,0.5\right)$.

Fig. 6 presents the results of estimating the TTE in systems with different numbers of servers and time horizons. Algorithm 2successfully estimates the small treatment effect, even when treating at most half of the units in the system ($\pi_{2}=0.5$). This demonstrates the effectiveness of our proposed framework for real-world applications, as it accounts for the complex interference effects and yields reliable estimates of treatment effects in diverse scenarios. For the CI heuristic, we set B=500 as before and q is selected to be 0.2, 0.15, 0.1 for N=500, N=2,000, and N=10,000, respectively. Consistent with earlier scenarios, we utilize the prior knowledge that the TTE must be between −1 and 0, as noted in Remark 3.

### 4.4. TTE Estimation at Other Treatment Levels.

**Fig. 7.**
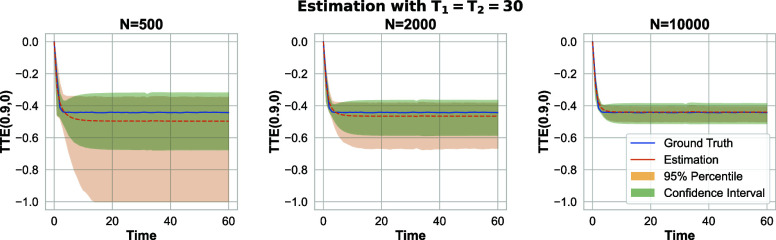
Server speed-up problem under a Bernoulli design: 95% CI for $\;{\text{TTE}}_{t}\left(0.9,0\right)$ estimation when $\left(\pi_{1},\pi_{2}\right)=\left(0.15,0.5\right)$.

In many practical scenarios, it is not possible to deliver the treatment to the entire population, either due to failure in delivery by the experimenter or due to resistance in adoption by the units. For instance, in the contagious disease example, some individuals may avoid using the new medication. In these settings, a broader family of estimands is necessary, as covered by Algorithm 2. Fig. 7 presents the results of estimating $\;{\text{TTE}}_{t}\left(0.9,0\right)$ in the parallel server system speed-up, while $\;{\text{TTE}}_{t}\left(0.9,0\right)$ for linear-in-means and binary outcome settings are deferred to *SI Appendix*, section 7. $\;{\text{TTE}}_{t}\left(0.9,0\right)$ compares the scenario of treating 90% of the population on average (with each experimental unit adopting the treatment with a probability of 0.9) to the scenario of treating no one. The results in Fig. 7 demonstrate the effectiveness of the proposed method for estimating a broader range of treatment effects, beyond just $\;{\text{TTE}}_{t}\left(1,0\right)$.

## 5. Conclusion

Estimating the total treatment effect in presence of network interference is an important scientific and practical question. Previous research has tackled this challenge by limiting interference to immediate neighbors, imposing structural constraints on interference, specializing outcome models, or innovating new estimands to overcome inherent difficulties. This study introduces the Causal Message-passing framework as a methodology for experimental design under unknown network interference. We provide a theoretical analysis of the framework and formulate one-dimensional representations of the high-dimensional outcomes.

In the context of Bernoulli randomized designs, we propose a strongly consistent estimator for the total treatment effect. To exhibit the adaptability of the proposed framework, we present several distinct case studies, with varying interference patterns and outcome specifications. These studies serve as a “proof-of-concept,” affirming the utility of Causal Message-passing in experiment design and analysis where interference patterns are unknown.

There are several directions left for future explorations. One immediate area is assessing applicability and robustness of the proposed methodology in more realistic contexts, such as real-world experimental design settings, with increased risks of model misspecification. On the theoretical side, an analysis of the CI estimation method presents an important avenue for exploration.

## Supplementary Material

Appendix 01 (PDF)

## Data Availability

There are no data underlying this work.

## Footnotes

^*^Rigorous statements are provided in section 3.

^†^The result still holds true if the function ψ is almost everywhere continuous in the first argument and continuous in the other arguments.

## References

[1] D. R. Cox, Planning of Experiments (Wiley, 1958).

[2] D. B. Rubin, Bayesian inference for causal effects: The role of randomization. Ann. Stat. **6**, 34–58 (1978).

[3] C. F. Manski, Identification of treatment response with social interactions. Econ. J. **16**, S1–S23 (2013).

[4] L. Forastiere, F. Mealli, A. Wu, E. M. Airoldi, Estimating causal effects under network interference with Bayesian generalized propensity scores. J. Mach. Learn. Res. **23**, 1–61 (2022).

[5] C. L. Yu, E. M. Airoldi, C. Borgs, J. T. Chayes, Estimating the total treatment effect in randomized experiments with unknown network structure. Proc. Natl. Acad. Sci. U.S.A. **119**, e2208975119 (2022). 36279463 10.1073/pnas.2208975119 PMC9636977

[6] G. W. Basse, E. M. Airoldi, Limitations of design-based causal inference and a/b testing under arbitrary and network interference. Sociol. Methodol. **48**, 136–151 (2018).

[7] V. Karwa, E. M. Airoldi, A systematic investigation of classical causal inference strategies under mis-specification due to network interference. arXiv [Preprint] (2018). https://arxiv.org/abs/1810.08259 (Accessed 31 August 2022).

[8] P. M. Aronow, C. Samii, Estimating average causal effects under general interference, with application to a social network experiment. Ann. Appl. Stat. **11**, 1912–1947 (2017).

[9] D. L. Sussman, E. M. Airoldi, Elements of estimation theory for causal effects in the presence of network interference. arXiv [Preprint] (2017). https://arxiv.org/abs/1702.03578 (Accessed 31 August 2022).

[10] M. Mezard, G. Parisi, M. Virasoro, Spin Glass Theory and Beyond, An Introduction to the Replica Method and Its Applications (World Scientific, Paris, France, 1986), p. 476.

[11] M. Mezard, A. Montanari, Information, Physics, and Computation (Oxford University Press, 2009).

[12] D. L. Donoho, A. Maleki, A. Montanari, Message-passing algorithms for compressed sensing. Proc. Natl. Acad. Sci. U.S.A. **106**, 18914–18919 (2009). 19858495 10.1073/pnas.0909892106 PMC2767368

[13] M. Bayati, A. Montanari, The dynamics of message passing on dense graphs, with applications to compressed sensing. IEEE Trans. Inf. Theory **57**, 764–785 (2011).

[14] G. W. Imbens, D. B. Rubin, Causal Inference in Statistics, Social, and Biomedical Sciences (Cambridge University Press, 2015).

[15] S. Athey, D. Eckles, G. W. Imbens, Exact p-values for network interference. J. Am. Stat. Assoc. **113**, 230–240 (2018).

[16] R. Xiong, S. Athey, M. Bayati, G. Imbens, Optimal experimental design for staggered rollouts. arXiv [Preprint] (2019). https://arxiv.org/abs/1911.03764 (Accessed 10 January 2022).

[17] K. Imai, Z. Jiang, Identification and sensitivity analysis of contagion effects in randomized placebo-controlled trials. J.R. Stat. Soc. Ser. A Stat. Soc. **183**, 1637–1657 (2020).

[18] C. F. Manski, Nonparametric bounds on treatment effects. Am. Econ. Rev. **80**, 319–323 (1990).

[19] P. M. Aronow, A general method for detecting interference between units in randomized experiments. Sociol. Methods Res. **41**, 3–16 (2012).

[20] J. Bowers, M. M. Fredrickson, C. Panagopoulos, Reasoning about interference between units: A general framework. Polit. Anal. **21**, 97–124 (2013).

[21] M. Saveski *et al*., “Detecting network effects: Randomizing over randomized experiments” in *Proceedings of the 23rd ACM SIGKDD International Conference on Knowledge Discovery and Data Mining* (2017), pp. 1027–1035.

[22] J. Pouget-Abadie , Testing for arbitrary interference on experimentation platforms. Biometrika **106**, 929–940 (2019).

[23] Y. Hu, S. Li, S. Wager, Average direct and indirect causal effects under interference. Biometrika **109**, 1165–1172 (2022).

[24] K. Han, S. Li, J. Mao, H. Wu, Detecting interference in a/b testing with increasing allocation. arXiv [Preprint] (2022). https://arxiv.org/abs/2211.03262 (Accessed 24 March 2023).

[25] M. P. Leung, Treatment and spillover effects under network interference. Rev. Econ. Stat. **102**, 368–380 (2020).

[26] D. Viviano, Experimental design under network interference. arXiv [Preprint] (2020). https://arxiv.org/abs/2003.08421 (Accessed 24 August 2023).

[27] M. P. Leung, Causal inference under approximate neighborhood interference. Econometrica **90**, 267–293 (2022).

[28] M. Cortez, M. Eichhorn, C. L. Yu, Exploiting neighborhood interference with low order interactions under unit randomized design. arXiv [Preprint] (2022). https://arxiv.org/abs/2208.05553 (Accessed 18 January 2023).

[29] M. Cortez, M. Eichhorn, C. Yu, “Staggered rollout designs enable causal inference under interference without network knowledge” in *Advances in Neural Information Processing Systems* (2022).

[30] A. Agarwal, S. Cen, D. Shah, C. L. Yu, Network synthetic interventions: A framework for panel data with network interference. arXiv [Preprint] (2022). https://arxiv.org/abs/2210.11355 (Accessed 20 October 2022).

[31] A. Belloni, F. Fang, A. Volfovsky, Neighborhood adaptive estimators for causal inference under network interference. arXiv [Preprint] (2022). https://arxiv.org/abs/2212.03683 (Accessed 7 December 2022).

[32] S. Li, S. Wager, Network interference in micro-randomized trials. arXiv [Preprint] (2022). http://arxiv.org/abs/2202.05356 (Accessed 31 July 2022).

[33] S. Li, S. Wager, Random graph asymptotics for treatment effect estimation under network interference. Ann. Stat. **50**, 2334–2358 (2022).

[34] P. R. Rosenbaum, Interference between units in randomized experiments. J. Am. Stat. Assoc. **102**, 191–200 (2007).

[35] O. Candogan, C. Chen, R. Niazadeh, Correlated cluster-based randomized experiments: Robust variance minimization (Chicago Booth Research Paper No. 21-17, 2021).

[36] E. Auerbach, M. Tabord-Meehan, The local approach to causal inference under network interference. arXiv [Preprint] (2021). https://arxiv.org/abs/2105.03810 (Accessed 31 July 2023).

[37] D. Eckles, B. Karrer, J. Ugander, Design and analysis of experiments in networks: Reducing bias from interference. J. Causal Inf. **5**, 20150021 (2016).

[38] J. Ugander, B. Karrer, L. Backstrom, J. Kleinberg, “Graph cluster randomization: Network exposure to multiple universes” in *Proceedings of the 19th ACM SIGKDD International Conference on Knowledge Discovery and Data Mining* (2013), pp. 329–337.

[39] J. Ugander, H. Yin, Randomized graph cluster randomization. J. Causal Inf. **11**, 20220014 (2023).

[40] A. Chin, Central limit theorems via Stein’s method for randomized experiments under interference. arXiv [Preprint] (2018). https://arxiv.org/abs/1804.03105 (Accessed 4 April 2023).

[41] R. Jagadeesan, N. S. Pillai, A. Volfovsky, Designs for estimating the treatment effect in networks with interference. Ann. Stat. **48**, 679–712 (2020).

[42] Y. Wang, C. Samii, H. Chang, P. Aronow, Design-based inference for spatial experiments with interference. arXiv [Preprint] (2020). https://arxiv.org/abs/2010.13599 (Accessed 4 April 2023).

[43] J. Cai, A. D. Janvry, E. Sadoulet, Social networks and the decision to insure. *Am. Econ. J.: Appl. Econ.* **7**, 81–108 (2015).

[44] D. Holtz, R. Lobel, I. Liskovich, S. Aral, Reducing interference bias in online marketplace pricing experiments. arXiv [Preprint] (2020). https://arxiv.org/abs/2004.12489 (Accessed 11 February 2023).

[45] S. Wager, K. Xu, Experimenting in equilibrium. Manage. Sci. **67**, 6694–6715 (2021).

[46] E. Munro, S. Wager, K. Xu, Treatment effects in market equilibrium. arXiv [Preprint] (2021). https://arxiv.org/abs/2109.11647 (Accessed 11 January 2023).

[47] R. Johari, H. Li, I. Liskovich, G. Y. Weintraub, Experimental design in two-sided platforms: An analysis of bias. Manage. Sci. **68**, 7069–7089 (2022).

[48] V. Farias, A. Li, T. Peng, A. Zheng, Markovian interference in experiments. Adv. Neural Inf. Process. Syst. **35**, 535–549 (2022).

[49] V. F. Farias *et al*., Correcting for interference in experiments: A case study at Douyin. arXiv [Preprint] (2023). https://arxiv.org/abs/2305.02542 (Accessed 4 May 2023).

[50] M. G. Hudgens, M. E. Halloran, Toward causal inference with interference. J. Am. Stat. Assoc. **103**, 832–842 (2012). 10.1198/016214508000000292 PMC2600548 19081744

[51] G. W. Basse, A. Feller, P. Toulis, Randomization tests of causal effects under interference. Biometrika **106**, 487–494 (2019).

[52] M. O. Jackson, Z. Lin, N. N. Yu, Adjusting for peer-influence in propensity scoring when estimating treatment effects. Available at SSRN 3522256 (2020).

[53] F. Sävje, P. Aronow, M. Hudgens, Average treatment effects in the presence of unknown interference. Ann. Stat. **49**, 673 (2021). 34421150 10.1214/20-aos1973 PMC8372033

[54] T. Ni, I. Bojinov, J. Zhao, Design of panel experiments with spatial and temporal interference. Available at SSRN 4466598 (2023).

[55] A. Boyarsky, H. Namkoong, J. Pouget-Abadie, Modeling interference using experiment roll-out. arXiv [Preprint] (2023). https://arxiv.org/abs/2305.10728 (Accessed 18 May 2023).

[56] C. Harshaw, F. Sävje, Y. Wang, A design-based Riesz representation framework for randomized experiments. arXiv [Preprint] (2022). https://arxiv.org/abs/2210.08698 (Accessed 24 October 2022).

[57] W. Li, D. L. Sussman, E. D. Kolaczyk, Causal inference under network interference with noise. arXiv [Preprint] (2021). https://arxiv.org/abs/2105.04518 (Accessed 31 August 2022).

[58] P. Goldsmith-Pinkham, G. W. Imbens, Social networks and the identification of peer effects. J. Business Econ. Stat. **31**, 253–264 (2013).

[59] P. Toulis, E. Kao, “Estimation of causal peer influence effects” in *International Conference on Machine Learning* (PMLR, 2013), pp. 1489–1497.

[60] L. E. Blume, W. A. Brock, S. N. Durlauf, R. Jayaraman, Linear social interactions models. J. Polit. Econ. **123**, 444–496 (2015).

[61] G. W. Basse, E. M. Airoldi, Model-assisted design of experiments in the presence of network-correlated outcomes. Biometrika **105**, 849–858 (2018).

[62] A. Chin, Regression adjustments for estimating the global treatment effect in experiments with interference. J. Causal Inference **7**, 20180026 (2019).

[63] Y. Jiang, H. Wang, Causal inference under network interference using a mixture of randomized experiments. arXiv [Preprint] (2023). https://arxiv.org/abs/2309.00141 (Accessed 31 August 2023).

[64] J. D. Angrist, The perils of peer effects. Labour Econ. **30**, 98–108 (2014).

[65] D. J. Thouless, P. W. Anderson, R. G. Palmer, Solution of “solvable model of a spin glass’’. Philos. Mag. **35**, 593–601 (1977).

[66] Y. Kabashima, A CDMA multiuser detection algorithm on the basis of belief propagation. J. Phys. A **36**, 11111–11121 (2003).

[67] E. Bolthausen, An iterative construction of solutions of the tap equations for the Sherrington–Kirkpatrick model. Commun. Math. Phys. **325**, 333–366 (2014).

[68] A. Javanmard, A. Montanari, State evolution for general approximate message passing algorithms, with applications to spatial coupling. Inf. Inference **2**, 115–144 (2013).

[69] M. Bayati, M. Lelarge, A. Montanari, Universality in polytope phase transitions and message passing algorithms. Ann. Appl. Probab. **25**, 753–822 (2015).

[70] R. Berthier, A. Montanari, P. M. Nguyen, State evolution for approximate message passing with non-separable functions. Inf. Inference **9**, 33–79 (2020).

[71] W. K. Chen, W. K. Lam, Universality of approximate message passing algorithms. arXiv [Preprint] (2020). https://arxiv.org/abs/2003.10431 (Accessed 17 March 2023).

[72] X. Zhong, T. Wang, Z. Fan, Approximate message passing for orthogonally invariant ensembles: Multivariate non-linearities and spectral initialization. arXiv [Preprint] (2021). 10.48550/arXiv.2110.02318 (Accessed 17 March 2023).

[73] T. Wang, X. Zhong, Z. Fan, Universality of approximate message passing algorithms and tensor networks. arXiv [Preprint] (2022). https://arxiv.org/abs/2206.13037 (Accessed 17 March 2023).

[74] R. Dudeja, Y. M. Lu, S. Sen, Universality of approximate message passing with semirandom matrices. Ann. Probab. **51**, 1616–1683 (2023).

[75] C. Rush, R. Venkataramanan, Finite sample analysis of approximate message passing algorithms. IEEE Trans. Inf. Theory **64**, 7264–7286 (2018).

[76] G. Li, Y. Wei, A non-asymptotic framework for approximate message passing in spiked models. arXiv [Preprint] (2022). https://arxiv.org/abs/2208.03313 (Accessed 17 March 2023).

[77] L. Zdeborová, F. Krzakala, Statistical physics of inference: Thresholds and algorithms. Adv. Phys. **65**, 453–552 (2016).

[78] A. Montanari, “Mean field asymptotics in high-dimensional statistics: From exact results to efficient algorithms” in *Proceedings of the International Congress of Mathematicians (ICM 2018)*, B. Sirakov, P. N. de Souza, M. Viana, Eds. (World Scientific, 2018), pp. 2973–2994.

[79] O. Y. Feng , A unifying tutorial on approximate message passing. Found. Trends Mach. Learn. **15**, 335–536 (2022).

[80] H. A. Bethe, W. L. Bragg, Statistical theory of superlattices. Proc. R. Soc. London Ser. A, Math. Phys. Sci. **150**, 552–575 (1935).

[81] R. Gallager, Low-density parity-check codes. IRE Trans. Inf. Theory **8**, 21–28 (1962).

[82] J. Pearl, “Reverend Bayes on inference engines: A distributed hierarchical approach” in *Proceedings of the Second National Conference on Artificial Intelligence* (AAAI Press, Menlo Park, CA/Pittsburgh, PA, 1982), pp. 133–136 (Retrieved 28 March 2009).

[83] P. Bajari *et al*., Multiple randomization designs. arXiv [Preprint] (2021). https://arxiv.org/abs/2112.13495 (Accessed 11 February 2023).

[84] K. Han, J. Ugander, Model-based regression adjustment with model-free covariates for network interference. arXiv [Preprint] (2023). https://arxiv.org/abs/2302.04997 (Accessed 10 February 2023).

[85] R. Kohavi, D. Tang, Y. Xu, Trustworthy Online Controlled Experiments: A Practical Guide to a/b Testing (Cambridge University Press, 2020).

[86] S. Gupta , Top challenges from the first practical online controlled experiments summit. SIGKDD Explor. Newsl. **21**, 20–35 (2019).

[87] S. Puffer, D. J. Torgerson, J. Watson, Cluster randomized controlled trials. J. Eval. Clin. Pract. **11**, 479–483 (2005). 16164589 10.1111/j.1365-2753.2005.00568.x

[88] J. Leskovec, J. Mcauley, Learning to discover social circles in ego networks. Adv. Neural Inf. Process. Syst. **25**, 539–547 (2012).

[89] P. Liao, P. Klasnja, A. Tewari, S. A. Murphy, Sample size calculations for micro-randomized trials in mHealth. Stat. Med. **35**, 1944–1971 (2016). 26707831 10.1002/sim.6847 PMC4848174

[90] P. Klasnja , Microrandomized trials: An experimental design for developing just-in-time adaptive interventions. Health Psychol. **34**, 1220 (2015). 10.1037/hea0000305 PMC4732571 26651463

[91] P. Klasnja , Efficacy of contextually tailored suggestions for physical activity: A micro-randomized optimization trial of heartsteps. Ann. Behav. Med. **53**, 573–582 (2019). 30192907 10.1093/abm/kay067 PMC6401341

[92] V. Gupta, M. H. Balter, K. Sigman, W. Whitt, Analysis of join-the-shortest-queue routing for web server farms. Perform. Eval. **64**, 1062–1081 (2007).

[93] X. Kuang, G. Mendelson, Detecting service slowdown using observational data. arXiv [Preprint] (2024). https://arxiv.org/abs/2401.07305 (Accessed 15 January 2024).

