# Supplementary material for: Causal message-passing for experiments with unknown and general network interference
Source: Proc Natl Acad Sci U S A. 2024 Sep 27;121(40):e2322232121. doi: 10.1073/pnas.2322232121 (PMC11459125; doi:10.1073/pnas.2322232121)
Supplement: Supplementary file 1 — Appendix 01 (PDF) [file pnas.2322232121.sapp.pdf]

Supplementary Appendices for

Causal Message Passing for Experiments with Unknown and General Network Interference

6. Further Extensions and Interpretations

In this section, we explore various ways to generalize and interpret the outcome specification Eq. (1). In §6.1, we begin by extending the results to a setting with direct effects and discuss how both direct and indirect effects can be estimated. This is followed by a different generalization where a non-linear random operator is applied to the right-hand side of Eq. (1). Next, we examine a neural network interpretation of Eq. (1) and briefly discuss extensions to more general interference patterns.

6.1. Direct and the Indirect Effects. In this section, we first start by discussing a slightly more general version of the outcome specification Eq. (1). Recall that the notation $\vec{W}_t$ refers to the treatment vector applied to all units at time t.

Specifically, we consider:

$$\vec{Y}_{t+1} = \big(\mathbf{A} + \mathbf{B}_t\big) g_t\left(\vec{Y}_t, \boldsymbol{W}, \boldsymbol{X}\right) + \tau_{\mathrm{DIR}}\vec{W}_{t+1} + \vec{\epsilon}_t\,, \qquad t = 0, 1, \ldots, T-1, \tag{12}$$

where a new term involving the unknown constant τ_{DIR}, which captures the *direct treatment effect*, is added. We will show that our results from the settings with $\tau_{\mathrm{DIR}} = 0$ can be applied here, through a change of variable. In addition, we will demonstrate that Algorithm 2, without any modification can be used to estimate TTE, i.e., Theorem 2 continues to hold. However, to decompose TTE into its two components, the direct effect τ_{DIR} and the indirect effect, we would need to introduce a new pre-processing step.

Definition 1 *If the treatment variables are binary, for any $t \in [T]$, the difference-in-means and Horvitz-Thompson estimators, denoted by $\hat{\tau}_{DM,t}$ and $\hat{\tau}_{HT,t}$ respectively, are defined as follows:*

$$\hat{\tau}_{DM,t} := \frac{\sum_{n=1}^N Y_t^n W_t^n}{\sum_{n=1}^N W_t^n} - \frac{\sum_{n=1}^N Y_t^n (1 - W_t^n)}{\sum_{n=1}^N (1 - W_t^n)} \tag{13}$$

$$\hat{\tau}_{HT,t} := \frac{1}{N}\sum_{n=1}^N \left(\frac{Y_t^n W_t^n}{\mathbb{E}[W_t^n]} - \frac{Y_t^n(1 - W_t^n)}{\mathbb{E}[1 - W_t^n]}\right)\,. \tag{14}$$

Next, we show that we can utilize our theoretical results from §3 to recover a version of the known result in the network interference literature (1, 2), which states that the difference-in-means and Horvitz-Thompson estimators estimate the direct effect.

Theorem 3 (Direct effect via difference-in-means and Horvitz Thompson) *Consider the same assumptions as in Theorem 1, along with the additional assumption that treatment variables are binary with means strictly away from 0 or 1, i.e., $\mathbb{E}[W_t] \notin \{0, 1\}$ for any $t \geq 1$. Then, $\hat{\tau}_{DM,t}$ and $\hat{\tau}_{HT,t}$ are strongly consistent estimators for τ_{DIR}., i.e.,*

$$\lim_{N\to\infty} \hat{\tau}_{DM,t} \overset{\text{a.s.}}{=} \tau_{DIR} \quad \textit{and} \quad \lim_{N\to\infty} \hat{\tau}_{HT,t} \overset{\text{a.s.}}{=} \tau_{DIR}\,.$$

Proof. Let us define, for all t, a new outcome process $\{\vec{\tilde{Y}}_t\}_{t\geq 0}$ involving the same treatment matrix $\boldsymbol{W}$, covariates $\boldsymbol{X}$, and interference matrices $\mathbf{A} + \mathbf{B}_t$, defined by:

$$\vec{\tilde{Y}}_t := \vec{Y}_0, \qquad \vec{\tilde{Y}}_t := \vec{Y}_t - \tau_{\mathrm{DIR}}\vec{W}_t, \quad t = 1, \ldots, T\,,$$

but using the updated function $\tilde{g}$, defined as

$$\tilde{g}_t\left(\vec{\tilde{Y}}_t, \boldsymbol{W}, \boldsymbol{X}\right) := g_t\left(\vec{\tilde{Y}}_t + \tau_{\mathrm{DIR}}\vec{W}_t, \boldsymbol{W}, \boldsymbol{X}\right)\,.$$

It is easy to see that Eq. (12) is equivalent to

$$\vec{\tilde{Y}}_{t+1} = \big(\mathbf{A} + \mathbf{B}_t\big)\tilde{g}_t\left(\vec{\tilde{Y}}_t, \boldsymbol{W}, \boldsymbol{X}\right) + \vec{\epsilon}_t\,, \qquad t = 0, 1, \ldots, T-1\,, \tag{15}$$

which is in the same format as the original Eq. (1). Moreover, Assumptions 1-3 hold for this updated process which means we can apply Theorem 1 and the updated state evolution equations,

$$\tilde{\nu}_{t+1} := (\mu + \mu_t)\mathbb{E}\left[\tilde{g}_t\big(\tilde{\nu}_t + \tilde{\rho}_t Z, \vec{W}, \vec{X}\big)\right], \qquad \tilde{\rho}_{t+1}^2 := (\sigma^2 + \sigma_t^2)\mathbb{E}\left[\tilde{g}_t\big(\tilde{\nu}_t + \tilde{\rho}_t Z, \vec{W}, \vec{X}\big)^2\right] + \sigma_e^2\,, \tag{16}$$

where $\lim_{N\to\infty}\sum_{n=1}^N \tilde{Y}_t^n / N \overset{\text{a.s.}}{=} \tilde{\nu}_t$.

By setting the function ψ in Theorem 1(a), once equal to $(\tilde{Y}_t^n + \tau_{\mathrm{DIR}} W_t^n) W_t^n$, and once equal to W_t^n, we obtain

$$\lim_{N\to\infty} \frac{1}{N}\sum_{n=1}^{N} Y_t^n W_t^n \overset{\text{a.s.}}{=} (\tilde{\nu}_t + \tau_{\mathrm{DIR}})\mathbb{E}[W_t] \quad \text{and} \quad \lim_{N\to\infty} \frac{1}{N}\sum_{n=1}^{N} W_t^n \overset{\text{a.s.}}{=} \mathbb{E}[W_t]\,,$$

respectively, where $W_t \sim \pi_t$. Similarly, we can obtain,

$$\lim_{N\to\infty} \frac{1}{N}\sum_{n=1}^{N} Y_t^n (1-W_t^n) \overset{\text{a.s.}}{=} \tilde{\nu}_t \mathbb{E}[(1-W_t)] \quad \text{and} \quad \lim_{N\to\infty} \frac{1}{N}\sum_{n=1}^{N} (1-W_t^n) \overset{\text{a.s.}}{=} \mathbb{E}[(1-W_t)]\,.$$

Therefore, since $\mathbb{E}[W_t] \notin \{0,1\}$,

$$\lim_{N\to\infty} \hat{\tau}_{\mathrm{DM},t} \overset{\text{a.s.}}{=} \tilde{\nu}_t + \tau_{\mathrm{DIR}} - \tilde{\nu}_t = \tau_{\mathrm{DIR}}\,.$$

which finishes the proof for the difference-in-means estimator. Proof for the Horvitz-Thompson estimator uses the same argument, plus recalling that for all $n \in [N]$, $W_t^n \sim \pi_t$, which means $\mathbb{E}[W_t^n] = \mathbb{E}[W_t]$. □

6.1.1. Estimating the indirect effect. Here, we first define the indirect (or network) effect, in light of the specification Eq. (12), and utilize Theorem 3, combined with a more general version of Theorem 2, to estimate the indirect effect.

We define the indirect treatment effect (ITE) by

$$\mathrm{ITE}_t(1,0) := \mathrm{TTE}_t(1,0) - \tau_{\mathrm{DIR}}\,. \tag{17}$$

Next, we show that Theorem 2 remains valid under the specification Eq. (12) when the treatment assignment is re-randomized in each time period.

Theorem 4 *Under the assumptions of Theorem 2, consider a two-stage Bernoulli experiment as in Algorithm 2 with outcome specification* Eq. (12)*, with the added assumption that the treatment assignments at each time period are selected independently of the past treatments, i.e. the vectors $\vec{W}_t$ are independent for all $t \in [T]$. Then, for any $\check{\pi}$, the output $\widehat{TTE}_t(\check{\pi},0)$ of the algorithm is a strongly consistent estimator for the total treatment effect; that is, for any $t \in [T]_0$, we have*

$$\lim_{N\to\infty} \widehat{TTE}_t(\check{\pi},0) \overset{\text{a.s.}}{=} TTE_t(\check{\pi},0). \tag{18}$$

Proof. Following the same argument as in proof of Theorem 2, we can assume Algorithm 2 is applied to the limited objects $\tilde{\nu}_t(\mathcal{E})$, rather than their finite sample variants. Next, utilizing the same outcome process, $\tilde{Y}_t$, as in proof of Theorem 3 and expanding the state evolution equation Eq. (16) for the averages and the fact that treatment at time t is independent of the one at time $t+1$, we obtain

$$\begin{aligned} \tilde{\nu}_{t+1}(\mathcal{E}) &\overset{\text{a.s.}}{=} \delta + \xi[\tilde{\nu}_t(\mathcal{E}) + \tau_{\mathrm{DIR}}\pi_1] + \lambda\pi_1 + \gamma[\tilde{\nu}_t(\mathcal{E}) + \tau_{\mathrm{DIR}}\pi_1]\pi_1 + \vec{\theta}^\top \vec{x}, \qquad && t = 0,\ldots,T_1-1,\\ \tilde{\nu}_{t+1}(\mathcal{E}) &\overset{\text{a.s.}}{=} \delta + \xi[\tilde{\nu}_t(\mathcal{E}) + \tau_{\mathrm{DIR}}\pi_2] + \lambda\pi_2 + \gamma[\tilde{\nu}_t(\mathcal{E}) + \tau_{\mathrm{DIR}}\pi_2]\pi_2 + \vec{\theta}^\top \vec{x}, \qquad && t = T_1,\ldots,T_1+T_2-1. \end{aligned} \tag{19}$$

Moreover, by using the function ψ, equal to $\tilde{Y}_t^n + \tau_{\mathrm{DIR}} W_t^n$, in Theorem 1(a), we get

$$\nu_t(\mathcal{E}) := \lim_{N\to\infty} \frac{1}{N}\sum_{n=1}^{N} Y_t^n \overset{\text{a.s.}}{=} \begin{cases} \tilde{\nu}_t(\mathcal{E}) + \tau_{\mathrm{DIR}}\pi_1 & t = 0,\ldots,T_1\,,\\ \tilde{\nu}_t(\mathcal{E}) + \tau_{\mathrm{DIR}}\pi_2 & t = T_1,\ldots,T_1+T_2\,. \end{cases}$$

Combining this with Eq. (19), we obtain

$$\begin{aligned} \nu_{t+1}(\mathcal{E}) &\overset{\text{a.s.}}{=} \delta + \xi\nu_t(\mathcal{E}) + (\lambda + \tau_{\mathrm{DIR}})\pi_1 + \gamma\nu_t(\mathcal{E})\pi_1 + \vec{\theta}^\top\vec{x}, \qquad && t = 0,\ldots,T_1-1,\\ \nu_{t+1}(\mathcal{E}) &\overset{\text{a.s.}}{=} \delta + \xi\nu_t(\mathcal{E}) + (\lambda + \tau_{\mathrm{DIR}})\pi_2 + \gamma\nu_t(\mathcal{E})\pi_2 + \vec{\theta}^\top\vec{x}, \qquad && t = T_1,\ldots,T_1+T_2-1. \end{aligned} \tag{20}$$

This means that, using an induction argument on t, the limits of sample averages for the experiment under specification Eq. (12), i.e., $\nu_t(\mathcal{E})$, are equal to the corresponding values if the outcomes followed specification Eq. (1), but with the mean coefficient λ in function g replaced by $\lambda + \tau_{\mathrm{DIR}}$. This statement holds for any values of π_1 and π_2, which means $\mathrm{TTE}_t(\check{\pi},0)$ would be equal in both scenarios. This also implies that the inputs to Algorithm 2 are the same under both scenarios. Therefore, we can invoke Theorem 2 in the latter scenario to show that the output of Algorithm 2, under either scenario, is a strongly consistent estimator for $\mathrm{TTE}_t(\check{\pi},0)$. This completes the proof. □

Remark 5 *Proof of Theorem 4 reveals that the addition of the direct effect in the outcome specification, i.e., the outcome sequence $\vec{Y}_t$ with functions g_t, has the same impact on the state evolution for the average of outcomes $\nu_t(\mathcal{E})$ and $TTE_t(\check{\pi},0)$ as increasing the mean coefficient λ of the functions g_t by the direct effect, i.e., the outcome sequence $\vec{\tilde{Y}}_t$ with functions $\tilde{g}_t$. In other words, in outcome sequence $\vec{Y}_t$, the combined direct and indirect effects appear as an indirect effect, which does not affect the TTE.*

Now, we can revisit our initial aim of estimating the indirect effect. Specifically, we can first apply Algorithm 2 to obtain $\widehat{\mathrm{TTE}}_t(1,0)$, which is a strongly consistent estimator for $\mathrm{TTE}_t(1,0)$. Then, we can obtain an estimate $\hat{\tau}_{\mathrm{DIR}}$ for the direct effect τ_{DIR} using the difference-in-means or Horvitz-Thompson estimator, and use the following estimate for the indirect treatment effect (ITE).

$$\widehat{\mathrm{ITE}}_t(1,0) = \widehat{\mathrm{TTE}}_t(1,0) - \hat{\tau}_{\mathrm{DIR}}, . \qquad [21]$$

Combining Theorems 3–4, we obtain the following result.

Corollary 1 (Consistency of ITE estimate) *Under the conditions of Theorems 3–4, the estimate in* Eq. (21) *is strongly consistent for the indirect effect, defined in* Eq. (17).

6.2. Generalized randomized outcome specifications. To justify the empirical success of Algorithm 2 as shown in §4, in settings beyond those covered by specification Eq. (1), we consider a more general version of Eq. (1), where the right-hand side is further transformed through a random function. We then provide a formal theorem for the applicability of Algorithm 2 for consistent estimation of the TTE under certain conditions.

For all $t \geq 0$, consider random functions $\Upsilon_t : \mathbb{R} \to \mathbb{R}$ such that, for all $x \in \mathbb{R}$, $\mathbb{E}_{\Upsilon_t}[\Upsilon_t(x)] = x$, where, with a slight abuse of notation, $\mathbb{E}_{\Upsilon_t}$ refers to taking the expectation with respect to the randomness in Υ_t, and $\mathbb{E}_{\Upsilon_{\leq t}}$ refers to taking the expectation with respect to the randomness of $\Upsilon_0, \ldots, \Upsilon_t$.

Two special cases are:

1. *Additive noise:* When $\Upsilon_t(x) = x + \epsilon_t$ for a centered random variable ϵ_t, similar to the one used in Eq. (1).

2. *Bernoulli noise:* In cases where $x \in [0,1]$, $\Upsilon_t(x)$ is a Bernoulli random variable with mean x.

We also follow the same convention as in prior sections that for any vector $\vec{V}$, the function Υ_t operates coordinatewise. Now, we introduce the following general outcome specification.

$$\vec{Y}_{t+1} = \Upsilon_t\left(\left(\mathbf{A} + \mathbf{B}_t\right) g_t\left(\vec{Y}_t, \boldsymbol{W}, \boldsymbol{X}\right)\right) \qquad t = 0, 1, \ldots, T-1, \qquad [22]$$

where the randomness of Υ_t is independent across all t as well as independent of all other sources of randomness in Eq. (22).

It is easy to see that Eq. (1) is a special case of Eq. (22) where the function Υ_t is additive noise that is independent across all n as well. Similarly, the case where Υ_t is independent Bernoulli noise across all n, bears similarity to the setting studied in §4.2.

Theorem 5 *Under the conditions of Theorem 2, as well as assuming that for all t,* $\lim_{N\to\infty} \sum_{n=1}^{N} \left(Y_t^n - \mathbb{E}_{\Upsilon_{\leq t-1}}[Y_t^n]\right)/N \overset{\text{a.s.}}{=} 0$. *Then, for any desired $\check{\pi} \in [0,1]$, Algorithm 2 provides a strongly consistent estimator for $TTE_t(\check{\pi}, 0)$ for all t, when applied to data generated from the generalized outcome specification* Eq. (22).

Proof. Similar to the proofs in §6.1, we define a new outcome process $\tilde{Y}_t$ as follows:

$$\vec{\tilde{Y}}_t := \mathbb{E}_{\Upsilon_{\leq t-1}}[\vec{Y}_t] \, .$$

We obtain:

$$\begin{aligned}
\vec{\tilde{Y}}_{t+1} &= \mathbb{E}_{\Upsilon_{\leq t-1}}\left[\mathbb{E}_{\Upsilon_t}\left[\left(\mathbf{A} + \mathbf{B}_t\right) g_t\left(\vec{Y}_t, \boldsymbol{W}, \boldsymbol{X}\right)\right]\right] \\
&= \mathbb{E}_{\Upsilon_{\leq t-1}}\left[\left(\mathbf{A} + \mathbf{B}_t\right) g_t\left(\vec{Y}_t, \boldsymbol{W}, \boldsymbol{X}\right)\right] \\
&\overset{(a)}{=} \mathbb{E}_{\Upsilon_{\leq t-1}}\left[\left(\mathbf{A} + \mathbf{B}_t\right) g_t\left(\vec{\tilde{Y}}_t, \boldsymbol{W}, \boldsymbol{X}\right) + \left(\mathbf{A} + \mathbf{B}_t\right) g_t'\left(\vec{\tilde{Y}}_t, \boldsymbol{W}, \boldsymbol{X}\right)(\vec{Y}_t - \vec{\tilde{Y}}_t)\right] \\
&\overset{(b)}{=} \left(\mathbf{A} + \mathbf{B}_t\right) g_t\left(\vec{\tilde{Y}}_t, \boldsymbol{W}, \boldsymbol{X}\right) ,
\end{aligned}$$

where (a) uses the fact that family of functions g_t, studied in Theorem 2 and here, are linear with respect to their first coordinate, and (b) uses $\mathbb{E}_{\Upsilon_{\leq t-1}}[\vec{Y}_t - \vec{\tilde{Y}}_t] = 0$, that holds by definition, and the fact that all the terms $\mathbf{A} + \mathbf{B}_t$, $g_t\left(\vec{\tilde{Y}}_t, \boldsymbol{W}, \boldsymbol{X}\right)$, and $g_t'\left(\vec{\tilde{Y}}_t, \boldsymbol{W}, \boldsymbol{X}\right)$ are independent of all randomness in $\Upsilon_0, \ldots, \Upsilon_{t-1}$.

Therefore, the outcome process $\vec{\tilde{Y}}_t$ satisfies specification Eq. (1), allowing us to invoke Theorem 1 and obtain state evolution equations. Following the same steps as in the proof of Theorem 3, and using the assumption that yields $\lim_{N\to\infty} \sum_{n=1}^{N} Y_t^n/N \overset{\text{a.s.}}{=} \lim_{N\to\infty} \sum_{n=1}^{N} \tilde{Y}_t^n/N$, for all t, one can show that the output of Algorithm 2, when applied to data generated from a two-stage Bernoulli experiment on outcomes of specification Eq. (22), provides a strongly consistent estimator for $\mathrm{TTE}_t(\check{\pi}, 0)$ for all t. □

6.3. Neural network interpretation. An alternative approach to interpret the specification Eq. (1) is motivated by the neural networks (3) and may explain the empirical performance of Causal-MP in §4, particularly its flexibility in capturing a wide range of outcome and interference patterns. Precisely, for a fixed $t > 0$, the potential outcome of the nth unit at time t, Y_t^n, is equal to the result of applying non-linear transformations to the outcomes of all units during previous time steps, weighted by linear operators. Figure 8 illustrates this, in a simpler setting without covariates and static interference patterns. Mathematically, Eq. (1) captures this interpretation and Y_t^n is the output of a neural network with $t+1$ layers (consisting of 1 input layer, $t-1$ hidden layers, and 1 output layer), where the input data is $\vec{Y}_0$. In this context, the elements of the interference matrix correspond to the weights in the neural network.

The neural network depiction of Eq. (1), alongside the proven ability of neural networks to encapsulate complex and nonlinear interactions—especially as discussed in the context of randomly weighted neural networks (4)—offers a promising avenue to study the Causal-MP framework. However, a distinct aspect of our approach, as informed by Eq. (5), is that as the number of units grows, our emphasis shifts to the estimation of the neural network's sufficient statistics regarding its weights, rather than the estimation of each individual weight.

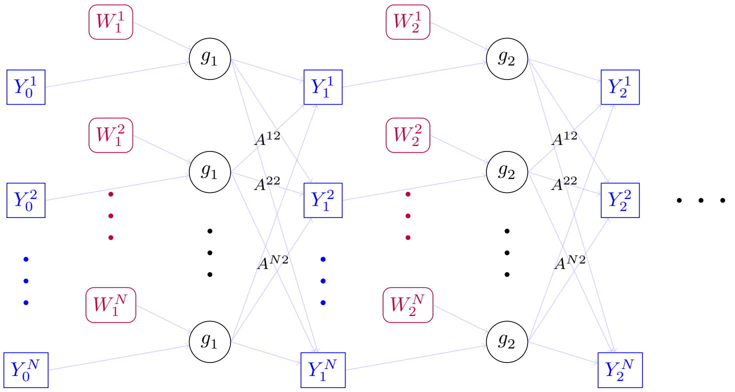

Fig. 8. Illustration of the potential outcome specification Eq. (1) as a neural network model.

6.4. Distribution of the interference matrix. The numerical simulations in §4 provide evidence that the predictions of Theorem 2 regarding the consistency of the estimates from Algorithm 2 are applicable in settings that do not necessarily satisfy the assumptions in our theoretical analysis. Focusing on Assumption 1 regarding the distribution of the interference matrix, in this section, we discuss why such iid Gaussian assumption can be relaxed to encompass a broader range of interference matrices.

As discussed in §1, a large body network interference literature adopts the neighborhood interference assumption; outcome of a unit i is obtained as a function of outcomes and treatments of all units in a neighborhood of i, where neighborhood is defined because of an explicit graph structure, e.g., (2, 5–15). First, we show that these settings can be mapped to our specification Eq. (1). Taking the linear-in-means model from §4.1, specifically Eq. (10), it can be writen as the following compact form

$$\vec{Y}_{t+1} = \alpha_1 + \mathbf{A}(\alpha_2\vec{Y}_t + \alpha_3\vec{W}_{t+1}) + \alpha_4\vec{W}_{t+1} + \varepsilon\,, \qquad [23]$$

where $\mathbf{A}$ is the row normalized adjacency matrix of the graph, representing the interference network, i.e., for units i and j, $\mathrm{A}^{ij} := E^{ij}/(\sum_{k=1}^{N} E^{ik})$. Following similar ideas as in §6.1, by a change of variable, $\vec{\tilde{Y}}_t := \vec{Y}_t - \alpha_1 - \alpha_4\vec{W}_t$, we can consider the specification

$$\vec{\tilde{Y}}_{t+1} = \mathbf{A}\left(\alpha_2[\vec{\tilde{Y}}_t + \alpha_1 + \alpha_4\vec{W}_t] + \alpha_3\vec{W}_{t+1}\right) + \varepsilon\,. \qquad [24]$$

Further, by defining $\tilde{g}_t(y, w) := \alpha_2(y + \alpha_1 + \alpha_4 w) + \alpha_3 w$, specification Eq. (24) becomes a special case of Eq. (1), except that the interference matrix $\mathbf{A}$ does not satisfy Assumption 1. However, when the interference network is a random graph or has a stochastic block structure (e.g., (2, 5, 16)), $\mathbf{A}$ becomes a random matrix. The literature on approximate message passing (AMP), in alignment with random matrix theory, provides evidence that one can adapt our analysis to obtain variants of Theorems 1-2 in settings when the entries of $\mathbf{A}$ are neither necessarily i.i.d. nor Gaussian.

Specifically, there is a substantial body of literature demonstrating that such random matrices exhibit a certain *universality property* in their asymptotic behavior. For instance, the literature on random matrix theory indicates that many asymptotic properties of the joint distribution of eigenvalues of these matrices are insensitive to the distribution of the matrix entries, under certain tail conditions, provided that the first two moments of the entries' distribution are preserved (17–19). Similarly, motivated by compressed sensing applications, the AMP literature highlights other asymptotic properties, such as state evolution of variants of Eq. (1), also exhibits a similar universality (20–23). In their context, the entries of $\mathbf{A}$ only need to be independent and are not required to follow the same distribution, and even allowing for a block structure in their moments. Further generalizations, such as when the interference matrix belongs to the family of rotationally invariant matrices (24), or other settings, have been studied by (25) and (26).

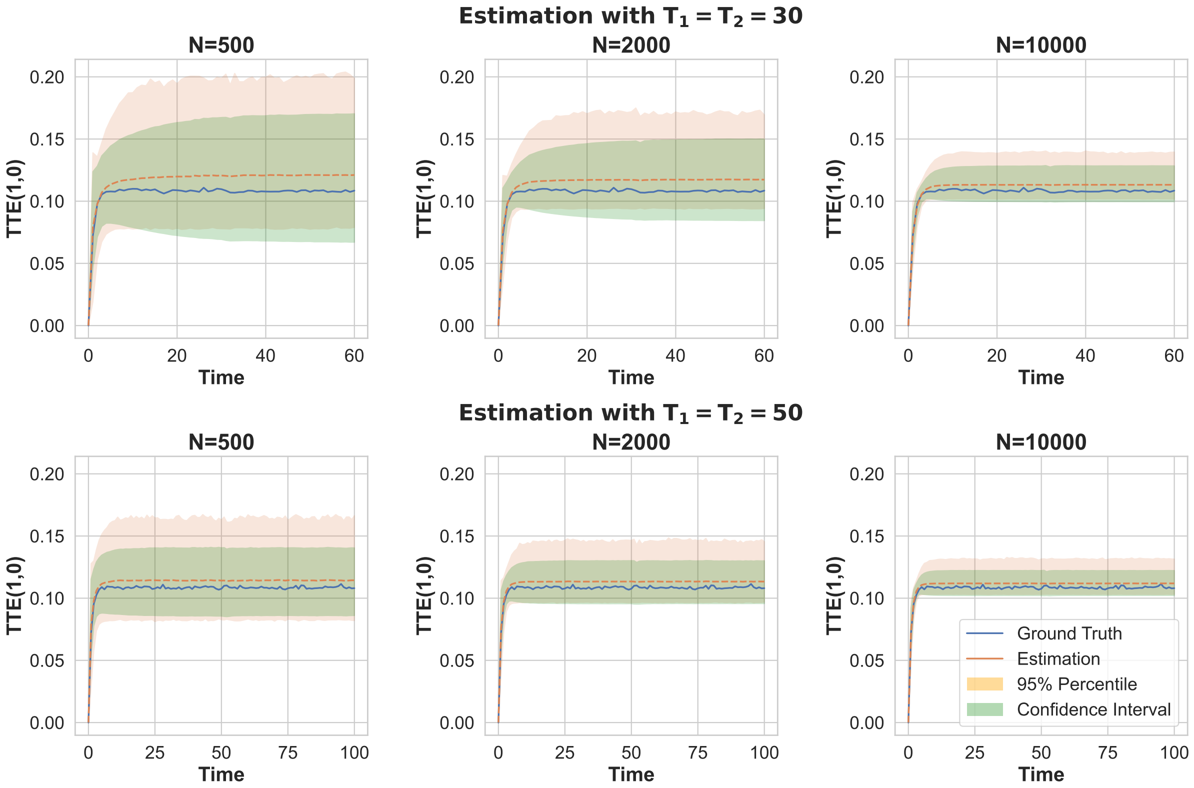

Fig. 9. Binary outcome model with Erdös-Rényi graph; 95% confidence interval for the total treatment effect estimation when $(\pi_1, \pi_2) = (0.25, 0.75)$.

Therefore, we expect generalizations of the state evolution and Theorems 1-2, to a broader class of interference matrices beyond those considered in Assumption 1 to be possible. It is also important to note that for cases where there is structure among the entries of $\mathbf{A}$, such as when the random graph potentially exhibits a clustered structure similar to the stochastic block model examined by (5) and used in §4, state evolution is expected to require modifications (20, 27–29). Exploring the implications of these modifications for estimating causal effects is an intriguing area for future research.

7. Additional Numerical Results

In this section, we provide more details on the experimental settings studied in §4, followed by additional numerical results that support the applicability and relevance of Algorithm 2 for estimating more general versions of the total treatment effect, defined in Eq. (2).

7.1. More details on linear-in-Means model with stochastic block network. To generate the stochastic block model with $N =$ 10,000, we consider the setting that units within each cluster are connected with a probability of 0.005, while units from different clusters have a probability of 0.0001 of being connected. These numbers for $N =$ 1,000 are 0.05 and 0.001, and for $N = 500$ are 0.1 and 0.002, respectively. Furthermore, we run Algorithm 2 in two stages with $T_1 = T_2 = 30$ with $(\pi_1, \pi_2) = (0.2, 0.5)$.

7.2. Binary outcome model with a burn-in period. Here, we consider another setup of the binary outcome model, in §4.2, with a burn-in period of length 10. Specifically, under Assumptions 3-5 in (16), which ensure the uniqueness of the stationary distribution of the underlying system, we set the parameter values as $(\alpha_1, \alpha_2, \alpha_3, \alpha_4, p_{\text{edge}}) = (0.5, 0.04, 0.04, 0.01, 3/N)$. Figure 9 presents the results of estimating the TTE. For the confidence interval estimation, we set $B = 500$ and $q = 0.7$ for all N. The results demonstrate a high level of accuracy in estimating the TTE, showcasing the robustness of the proposed framework.

7.3. TTE estimation at other treatment levels. Here, we present the results of estimating $\text{TTE}_t(0.9, 0)$ in two other settings: the linear-in-means model with a random geometric graph (Figure 10) and the binary outcome model (Figure 11), both highlighting the robustness of the proposed method.

7.4. TTE estimation with seasonal time trend. In the final setting, we consider the parallel server system and incorporate a weekly time trend, along with independent noise across time steps, in the demand rate for the servers. This means that the arrival rate to the queueing system varies randomly over time while following a specific trend. Figure 12 illustrates the results, showcasing the robustness of the proposed framework.

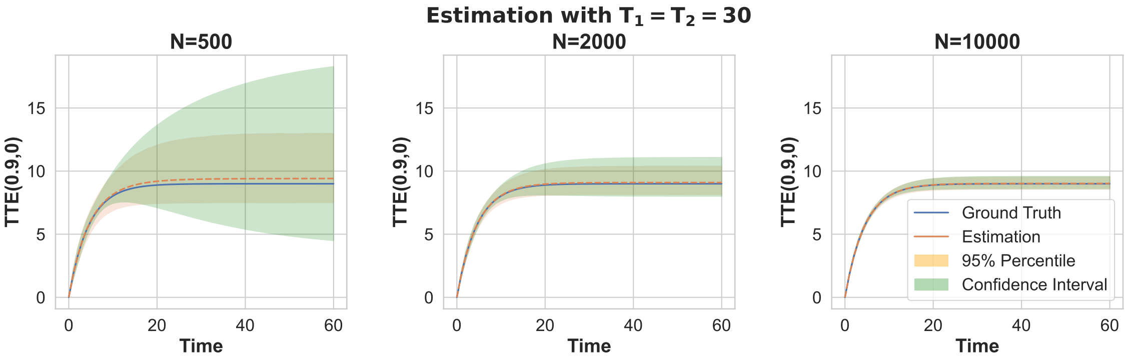

Fig. 10. Linear-in-means model with random geometric graph; 95% confidence intervals for $\text{TTE}_t(0.9, 0)$ estimation when $(\pi_1, \pi_2) = (0.2, 0.5)$.

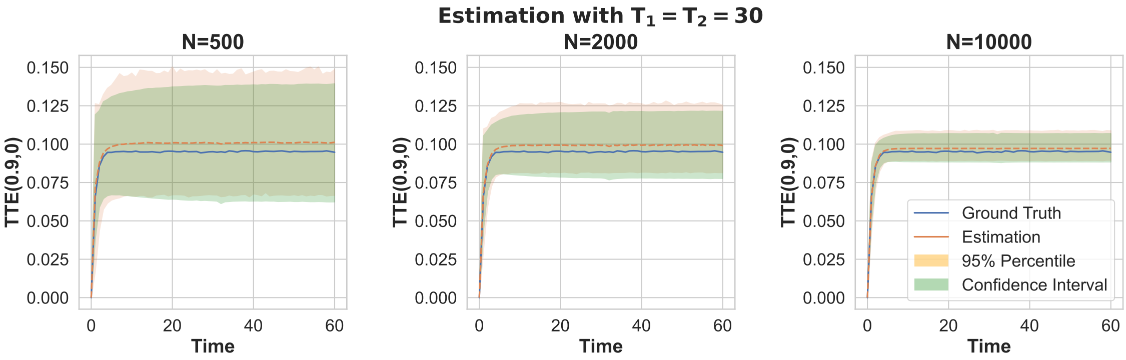

Fig. 11. Binary outcome model with micro-randomized trial: 95% confidence interval for $\text{TTE}_t(0.9, 0)$ estimation when $(\pi_1, \pi_2) = (0.25, 0.75)$.

The inclusion of a time-varying demand rate adds complexity to the model, simulating more realistic scenarios where demand fluctuates. Despite these variations, the framework consistently provides accurate and reliable estimates of the treatment effect. Specifically, Algorithm 2 yields a reliable estimate of the TTE even without accounting for heterogeneous variations over time. This further demonstrates the versatility and strength of our approach in handling dynamic and noisy environments, confirming its applicability to real-world situations where conditions are far from static.

8. Detailed Explanation of Treatment Effects

Direct Effect. The direct effect represents the immediate influence of the medication on the severity of symptoms for unit 1, following the first period of administration. This effect is specific to the treated units and stems directly from the treatment (30, 31).

Treatment Spillover Effect. Consider a hospital environment where the treatment for unit 1 involves the use of a highly effective air purifier designed to remove airborne pathogens. While the immediate goal is to alleviate unit 1's respiratory symptoms, the

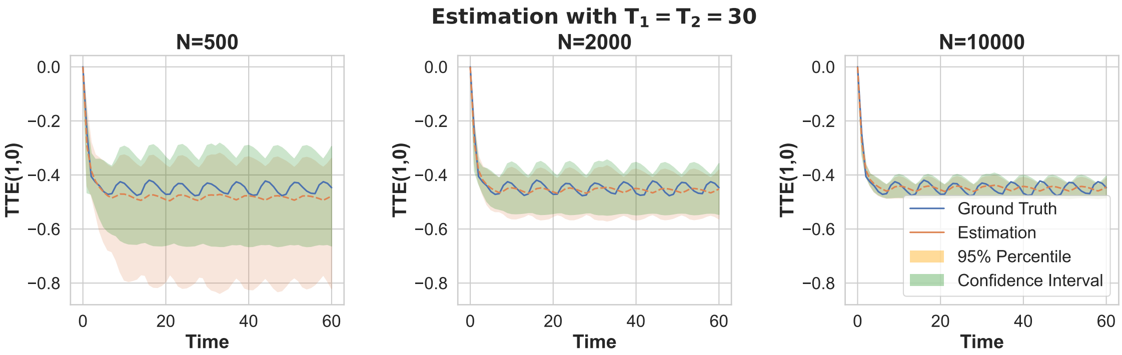

Fig. 12. Server speed-up problem with a seasonal time trend: 95% confidence interval for the TTE estimation when $(\pi_1, \pi_2) = (0.15, 0.5)$.

purifier also cleans the air in the shared space, indirectly benefiting unit 2. This is an example of how the treatment of one unit can influence the outcomes of another (30, 31).

Carryover Effect. A carryover effect implies that the effects of the medication continue to influence the health status of the units in subsequent periods, beyond the immediate treatment (32).

Unit Peer Effect. The unit peer effect is especially significant in contagious diseases, where the severity of symptoms in one unit directly impacts the severity experienced by other units (13, 33).

Autocorrelation. Autocorrelation refers to the temporal interdependence of outcomes within the same unit. For example, severe symptoms today increase the likelihood of continued illness tomorrow.

Anticipation Effect. The anticipation effect describes how units might adjust their current behavior based on expected future treatments or outcomes, adding a layer of complexity to the network.

9. Network Interference as a Massage Passing Model

Here we expand on the message-passing interpretation of the specification Eq. (1) from §2. Specifically, we note that in the literature on message-passing algorithms, the message from unit i to n differs slightly from the description in §2. Specifically, the message from each unit i to n is as described, but it omits the message received from n in the prior period. If we represent the message from unit i to n at time $t+1$ by $U_{t+1}^{i\to n}$, then $U_{t+1}^{i\to n} = g_t(\sum_{j\in[N]\setminus n} \mathrm{G}_t^{ij} U_t^{j\to i}, \vec{W}^j, \vec{X}^j)$. However, in the large N regime, this is approximately equivalent to the simpler specification

$$Y_{t+1}^n = \sum_{i\in[N]} \mathrm{G}_t^{ni} \underbrace{g_t(Y_t^i, \vec{W}^i, \vec{X}^i)}_{\textbf{Message of } i \textbf{ to } n} . \qquad [25]$$

This approximation underpins the AMP algorithm. For completeness, we provide this heuristic argument, that has also appeared in AMP literature, below. Additionally, in the AMP literature, this exercise often leads to a dynamic as in Eq. (1) that also incorporates a memory term involving $g_{t-1}(\vec{Y}_{t-1}, \boldsymbol{W}, \boldsymbol{X})$, also known as the Onsager term. However, in our context, this term disappears since the matrix $\mathbf{G}_t$ is not symmetric.

Next, we present a heuristic derivation of the potential outcome model. We proceed by writing the message-passing model as follows.

$$U_0^{n\to m} = g_0\left(Y_0^n, \vec{W}^n, \vec{X}^n\right), \quad U_{t+1}^{n\to m} = g_t\left(\sum_{i\in[N]\setminus m} \mathbf{G}_{ni} U_t^{i\to n}, \vec{W}^n, \vec{X}^n\right). \qquad [26]$$

Note that on the right-hand side of the second equation in Eq. (26), we encounter a summation of order N terms. Furthermore, its dependence on m is solely through the exclusion of the term $U_t^{m\to n}$. As a result, we can follow a similar line of reasoning as presented in Appendix A of (34), for $n, m \in [N]$, we write

$$U_t^{n\to m} = U_t^n + \delta U_t^{n\to m}, \qquad [27]$$

where $\delta U_t^{n\to m} = O(N^{-1/2})$ and U_t^n is a term independent of m. Considering Eq. (26) and Eq. (27) together, we get

$$U_{t+1}^n + \delta U_{t+1}^{n\to m} = g_t\left(\sum_{i\in[N]} \mathbf{G}_{ni}\left(U_t^i + \delta U_t^{i\to n}\right) - \mathbf{G}_{nm}\left(U_t^m + \delta U_t^{m\to n}\right), \vec{W}^n, \vec{X}^n\right).$$

Assuming that the function g_t is continuous and differentiable in the first argument as well as $\mathbf{G}_{nm} \sim \mathcal{N}\big((\mu+\mu_t)/N, (\sigma+\sigma_t)/N\big)$, for large values of N, we can write

$$U_{t+1}^n + \delta U_{t+1}^{n\to m} \approx g_t\left(\sum_{i\in[N]} \mathbf{G}_{ni}\left(U_t^i + \delta U_t^{i\to n}\right) - \mathbf{G}_{nm} U_t^m, \vec{W}^n, \vec{X}^n\right).$$

Then, writing the first-order approximation, we have

$$\begin{aligned} U_{t+1}^n + \delta U_{t+1}^{n\to m} \approx\ & g_t\left(\sum_{i\in[N]} \mathbf{G}_{ni}\left(U_t^i + \delta U_t^{i\to n}\right), \vec{W}^n, \vec{X}^n\right) \\ & - g_t^{'}\left(\sum_{i\in[N]} \mathbf{G}_{ni}\left(U_t^i + \delta U_t^{i\to n}\right), \vec{W}^n, \vec{X}^n\right) \mathbf{G}_{nm} U_t^m, \end{aligned} \qquad [28]$$

where $g_t^{'}$ denotes derivative with respect to the first argument. Note that the last term is the only term involved with m on the right-hand side; thus, we can argue that

$$\delta U_{t+1}^{n\to m} \approx -g_t^{'}\left(\sum_{i\in[N]} \mathbf{G}_{ni}\left(U_t^i + \delta U_t^{i\to n}\right), \vec{W}^n, \vec{X}^n\right) \mathbf{G}_{nm} U_t^m. \qquad [29]$$

Substituting the result of Eq. (29) in Eq. (28) implies that

$$U_{t+1}^n + \delta U_{t+1}^{n\to m} \approx g_t\Bigg(\sum_{i\in[N]} \mathbf{G}_{ni} U_t^i - \sum_{i\in[N]} g_t^{'}\Big(\sum_{j\in[N]} \mathbf{G}_{nj}\big(U_t^j + \delta U_t^{j\to i}\big)\Big)\mathbf{G}_{in}\mathbf{G}_{ni}U_t^n, \vec{W}^n, \vec{X}^n\Bigg)$$
$$- g_t^{'}\Bigg(\sum_{i\in[N]} \mathbf{G}_{ni}\big(U_t^i + \delta U_t^{i\to n}\big), \vec{W}^n, \vec{X}^n\Bigg)\mathbf{G}_{nm}U_t^m.$$

Note that $\mathbf{G}_{in}$ and $\mathbf{G}_{ni}$ are two independent Gaussian random variables. Therefore, taking limit as $N \to \infty$, we get

$$U_{t+1}^n = g_t\Bigg(\sum_{i\in[N]} \mathbf{G}_{ni} U_t^i, \vec{W}^n, \vec{X}^n\Bigg). \quad [30]$$

Letting $Y_{t+1}^n = \sum_{i\in[N]} \mathbf{G}_{ni} U_t^i$, we obtain the desired result.

10. Total treatment effect estimation at the equilibrium

Here, we use the state evolution equations Eq. (4) to establish an estimator for the TTE at equilibrium. This stands in contrast to Algorithm 2, which is tailored to estimate the total treatment effect over the entire time horizon. This equilibrium estimand, denoted as $\mathrm{TTE}(1,0)$, characterizes the TTE as the time horizon extends towards infinity, formally expressed as $\lim_{T\to\infty} \mathrm{TTE}_T(1,0)$. Subsequently, with access to two sets of observations of individuals' outcomes at the equilibrium, denoted as $\vec{Y}(\pi_1)$ and $\vec{Y}(\pi_2)$, we define the following estimator:

$$\widehat{\mathrm{TTE}}(1,0) = \frac{1}{\pi_2 - \pi_1} \sum_{n=1}^{N} \frac{Y^n(\pi_2) - Y^n(\pi_1)}{N}. \quad [31]$$

The estimator presented in Eq. (31) is inspired by the work of (13) and extends a version analyzed by them. They demonstrated that with access to average baseline outcomes before an experiment, a special case of this estimator is unbiased under the neighborhood interference assumption and a linear outcome model. Our adaptation, which utilizes the function family g from Eq. (6), enables the examination of non-linear outcomes. We find that the estimator Eq. (31) is subject to bias, with the degree of bias linked to the expected values of $\mathbb{E}[\Gamma^n]$ and $\mathbb{E}[\Xi^n]$. We formalize the statement below.

Note that we cannot let $T \to \infty$ as the results of Theorem 1 hold true for finite values of T. Therefore, we formally assume that for some sufficiently large value of T, the quantity $\mathrm{TTE}_T(1,0)$ stabilizes, and we denote its value as $\mathrm{TTE}(1,0)$. For any experimental design $\mathcal{E}$, we also denote $\hat{\nu}(\mathcal{E}) := \frac{1}{N}\sum_{n=1}^{N} Y_T^n$; that is the sample mean at the equilibrium state under design $\mathcal{E}$. We then have the following result.

Theorem 6 *Let $\widehat{TTE}(1,0)$ be the estimator defined by* Eq. (31)*. Then, for any two values of $\pi_2 \neq \pi_1$, we have:*

$$\lim_{N\to\infty} \widehat{TTE}(1,0) \overset{\text{a.s.}}{=} TTE(1,0) + \frac{\gamma}{1-\xi} \lim_{N\to\infty}\left(\frac{\pi_2\hat{\nu}(\pi_2) - \pi_1\hat{\nu}(\pi_1)}{\pi_2 - \pi_1} - \hat{\nu}(1)\right), \quad [32]$$

where $\xi = \mathbb{E}[\Xi^n]$ and $\gamma = \mathbb{E}[\Gamma^n]$.

The following corollary can be readily obtained as a special case of Theorem 6.

Corollary 2 *If $\gamma = 0$, then, $\widehat{TTE}(1,0)$ is a strongly consistent estimator for the total treatment effect at the equilibrium $TTE(1,0)$.*

Given access to historical data before the experiment, and by setting $\pi_1 = 0$ in Theorem 6, we can simplify the bias term to $\frac{\gamma}{1-\xi}\left(\hat{\nu}(\pi_2) - \hat{\nu}(1)\right)$. Consequently, if we can establish bounds on the values of γ, ξ, and $\hat{\nu}(1)$ based on system characteristics, we can place bounds on the bias of the estimation using the estimator in Eq. (31).

11. Detailed Proofs of Technical Results

Below, we first introduce the necessary notations required for the proofs. Then, we focus on proving Theorem 1 in multiple steps. We begin by stating Theorem 7 as a generalization of Theorem 1. Subsequently, we describe the conditioning technique and present related results for adopting this technique for our specific purpose. This includes deriving the conditional distribution of the fixed interference matrix given observations of outcomes and treatments up to a certain point.

Next, we utilize an induction argument to prove the main results in two major steps. Following that, we provide a detailed proof for the consistency statement of Algorithm 2 in Theorem 2. Finally, we conclude this section by presenting two versions of the law of larger numbers that are frequently used in the proofs.

11.1. Notations and Preliminaries. For any set S, the indicator function $\mathbb{1}_S(\omega)$ evaluates to 1 if ω belongs to S, and 0 otherwise. We define $\mathbb{R}^{n\times m}$ as the set of matrices with n rows and m columns. Given a matrix $\boldsymbol{M}$, we denote its transpose as $\boldsymbol{M}^\top$, its Frobenius norm as $\|\boldsymbol{M}\|_F$, and its trace as $\mathrm{Tr}(\boldsymbol{M})$. Additionally, we represent a matrix of ones with dimensions $n \times m$ as $\mathbf{1}_{n\times m} \in \mathbb{R}^{n\times m}$. The symbol $\overset{\mathrm{d}}{=}$ is used to denote equality in distribution, while $\overset{\text{a.s.}}{=}$ is used for equalities that hold almost surely. For $t \geq 1$, we define

$$\bar{\nu}_{t+1} := \mu\mathbb{E}\left[g_t\big(\nu_t + \rho_t Z, \vec{W}, \vec{X}\big)\right], \qquad \bar{\rho}^2_{t+1} := \sigma^2\mathbb{E}\left[g_t\big(\nu_t + \rho_t Z, \vec{W}, \vec{X}\big)^2\right], \tag{33}$$

where $Z \sim \mathcal{N}(0,1)$ independent from $(\vec{W}, \vec{X}) \sim \Pi \times p_{\boldsymbol{X}}$ and ν_t as well as ρ_t are defined in Eq. (4). Further, letting $\vec{U}_t = g_t\big(\vec{Y}_t, \boldsymbol{W}, \boldsymbol{X}\big)$ as well as $\vec{U}_{tn} = g_t\big(Y_t^n, \vec{W}^n, \vec{X}^n\big)$, we denote

$$\boldsymbol{Q}_t := \left[\vec{U}_0\middle|\vec{U}_1\middle|\dots\middle|\vec{U}_{t-1}\right], \qquad \boldsymbol{R}_t := \left[\vec{Y}_1 - \mathbf{B}_0\vec{U}_0 - \vec{\epsilon}_0\middle|\dots\middle|\vec{Y}_t - \mathbf{B}_{t-1}\vec{U}_{t-1} - \vec{\epsilon}_{t-1}\right]. \tag{34}$$

That is, $\boldsymbol{Q}_t$ and $\boldsymbol{R}_t$ are matrices with columns of $\vec{U}_{s-1}$ and $\vec{Y}_s - \mathbf{B}_{s-1}\vec{U}_{s-1} - \vec{\epsilon}_{s-1}$, when $s = 1, \dots, t$, respectively. Then, we use $\vec{U}_t^{\|}$ to denote the projection of $\vec{U}_t$ onto the column space of $\boldsymbol{Q}_t$ and accordingly define $\vec{U}_t^{\perp} = \vec{U}_t - \vec{U}_t^{\|}$. Further, let $\vec{\alpha}_t = (\alpha_0, \alpha_1, \dots, \alpha_{t-1})^\top$ be such that

$$\vec{U}_t^{\|} = \sum_{s=0}^{t-1}\alpha_s\vec{U}_s = \sum_{s=0}^{t-1}\alpha_s g_s\big(\vec{Y}_s, \boldsymbol{W}, \boldsymbol{X}\big). \tag{35}$$

For vectors $\vec{u}, \vec{v} \in \mathbb{R}^m$, we define the scalar product $\langle\vec{u}, \vec{v}\rangle := \frac{1}{m}\sum_{i=1}^m u_i v_i$. Then, in Eq. (35), we have

$$\vec{\alpha}_t = \big(\boldsymbol{Q}_t^\top\boldsymbol{Q}_t\big)^{-1}\boldsymbol{Q}_t^\top\vec{U}_t. \tag{36}$$

11.2. General Result: Proof of Theorem 1. Here, we state Theorem 7 which is an expanded version of Theorem 1.

Theorem 7 *Fixing $k \geq 2$, assume the sequence of initial outcomes $\vec{Y}_0$, the treatment assignments $\boldsymbol{W}$, as well as the covariates $\boldsymbol{X}$ are given and suppose Assumption 3 holds. Then, we have the following statements for all $t \geq 0$.*

(a) For any function $\psi : \mathbb{R}^{t+1+T+M} \mapsto \mathbb{R}$ that $\psi \in \mathcal{CP}(k)$, we have

$$\lim_{N\to\infty}\frac{1}{N}\sum_{n=1}^N\psi\big(Y_1^n, \dots, Y_{t+1}^n, \vec{W}^n, \vec{X}^n\big) \overset{\text{a.s.}}{=} \mathbb{E}\Big[\psi\Big(\nu_1 + \rho_1 Z_1, \dots, \nu_{t+1} + \rho_{t+1}Z_{t+1}, \vec{W}, \vec{X}\Big)\Big], \tag{37}$$

where $Z_s \sim \mathcal{N}(0,1)$, $s = 1, \dots, t+1$, independent of $(\vec{W}, \vec{X}) \sim \Pi \times p_{\boldsymbol{X}}$.

(b) For all $0 \leq r \neq s \leq t$, the following equations hold and all limits exist, are bounded, and have degenerate distribution (i.e. they are constant random variables)

$$\lim_{N\to\infty}\frac{1}{N}\sum_{n=1}^N Y_{s+1}^n \overset{\text{a.s.}}{=} \lim_{N\to\infty}\frac{\mu+\mu_s}{N}\sum_{n=1}^N U_s^n \overset{\text{a.s.}}{=} \nu_{s+1}, \tag{38a}$$

$$\lim_{N\to\infty}\frac{1}{N}\sum_{n=1}^N\Big(Y_{s+1}^n - B_s^{n\cdot}\vec{U}_s - \epsilon_s^n\Big) \overset{\text{a.s.}}{=} \lim_{N\to\infty}\frac{\mu}{N}\sum_{n=1}^N U_s^n \overset{\text{a.s.}}{=} \bar{\nu}_{s+1}, \tag{38b}$$

$$\lim_{N\to\infty}\frac{1}{N}\sum_{n=1}^N(Y_{s+1}^n)^2 \overset{\text{a.s.}}{=} \nu_{s+1}^2 + \lim_{N\to\infty}\frac{\sigma^2+\sigma_s^2}{N}\sum_{n=1}^N(U_s^n)^2 + \sigma_e^2 \overset{\text{a.s.}}{=} \nu_{s+1}^2 + \rho_{s+1}^2, \tag{38c}$$

$$\lim_{N\to\infty}\frac{1}{N}\sum_{n=1}^N Y_{s+1}^n Y_{r+1}^n \overset{\text{a.s.}}{=} \nu_{s+1}\nu_{r+1} + \lim_{N\to\infty}\frac{\sigma^2}{N}\sum_{n=1}^N U_s^n U_r^n, \tag{38d}$$

$$\lim_{N\to\infty}\frac{1}{N}\sum_{n=1}^N\Big(Y_{s+1}^n - B_s^{n\cdot}\vec{U}_s - \epsilon_s^n\Big)Y_{r+1}^n \overset{\text{a.s.}}{=} \lim_{N\to\infty}\frac{\mu(\mu+\mu_r)}{N^2}\left(\sum_{n=1}^N U_s^n\right)\left(\sum_{n=1}^N U_r^n\right) + \lim_{N\to\infty}\frac{\sigma^2}{N}\sum_{n=1}^N U_s^n U_r^n, \tag{38e}$$

$$\lim_{N\to\infty}\frac{1}{N}\sum_{n=1}^N\Big(Y_{s+1}^n - B_s^{n\cdot}\vec{U}_s - \epsilon_s^n\Big)\Big(Y_{r+1}^n - B_r^{n\cdot}\vec{U}_r - \epsilon_r^n\Big) \overset{\text{a.s.}}{=} \lim_{N\to\infty}\frac{\mu^2}{N^2}\left(\sum_{n=1}^N U_s^n\right)\left(\sum_{n=1}^N U_r^n\right) + \lim_{N\to\infty}\frac{\sigma^2}{N}\sum_{n=1}^N U_s^n U_r^n. \tag{38f}$$

(c) For all $s = 1, \dots, t$, the following matrices are positive definite almost surely:

$$\lim_{N\to\infty} \frac{\boldsymbol{Q}_s^\top \boldsymbol{Q}_s}{N} \succ 0, \qquad \lim_{N\to\infty} \frac{\boldsymbol{V}_s^\top \boldsymbol{V}_s}{N} - \lim_{N\to\infty} \frac{\boldsymbol{V}_s^\top \mathbf{1}_{N\times 1}}{N} \lim_{N\to\infty} \frac{\mathbf{1}_{1\times N} \boldsymbol{V}_s}{N} \succ 0. \qquad [39]$$

In the following section, we will provide a comprehensive explanation of the conditioning technique, which will be employed to establish the results presented in Theorem 7.

11.3. Conditioning Technique. Let $\mathcal{G}_t$ denote the σ-algebra generated by $\vec{Y}_0, \vec{Y}_1, \dots, \vec{Y}_t$, $\boldsymbol{W}$, $\boldsymbol{X}$, $\mathbf{B}_0, \dots, \mathbf{B}_{t-1}$, as well as $\vec{\epsilon}_0, \vec{\epsilon}_1, \dots, \vec{\epsilon}_{t-1}$. We calculate the conditional distribution of $\mathbf{A}$ given $\mathcal{G}_t$. On the other hand, conditioning on $\mathcal{G}_t$ is equivalent to conditioning on the event

$$\mathbf{A}\boldsymbol{Q}_t = \boldsymbol{R}_t. \qquad [40]$$

Note that, given $\mathcal{G}_t$, entries of both $\boldsymbol{Q}_t$ and $\boldsymbol{R}_t$ are deterministic known real numbers. Then, we need a generalization of Lemma 11 in (34) which is based on the invariance property of the Gaussian distribution under rotations.

Lemma 1 *Let $\boldsymbol{D} \in \mathbb{R}^{m\times n}$ be a full-row rank matrix and $\vec{V} \in \mathbb{R}^n$ a vector with i.i.d. Gaussian entries with mean γ and variance χ^2. Then, for any vector $\vec{b} \in \mathbb{R}^m$, we have*

$$\vec{V}|_{\boldsymbol{D}\vec{V}=\vec{b}} \overset{\mathrm{d}}{=} \gamma \mathbf{1}_n + \boldsymbol{D}^\top \left(\boldsymbol{D}\boldsymbol{D}^\top\right)^{-1} \vec{d} + P_{\{\boldsymbol{D}\vec{J}=0\}}(\vec{S}), \qquad [41]$$

where $\vec{d} = \vec{b} - \gamma \boldsymbol{D}\mathbf{1}_n$ and $\vec{J} = \vec{V} - \gamma \mathbf{1}_n$. Further, $\vec{S}$ is a random vector independent of $\vec{J}$ with the same distribution, $P_{\{\boldsymbol{D}\vec{J}=0\}}$ is the orthogonal projection onto the subspace $\{\boldsymbol{D}\vec{J}=0\}$, and $\boldsymbol{D}^\top \left(\boldsymbol{D}\boldsymbol{D}^\top\right)^{-1} \vec{d} = \mathrm{argmin}_{\vec{J}} \left\{ \left\|\vec{J}\right\|^2 : \boldsymbol{D}\vec{J} = \vec{d} \right\}$.

Proof. By definition, $\vec{J} \in \mathbb{R}^n$ is a random vector with i.i.d. Gaussian entries with zero mean and variance χ^2. We have

$$\vec{V}|_{\boldsymbol{D}\vec{V}=\vec{b}} \overset{\mathrm{d}}{=} \gamma \mathbf{1}_n + \vec{J}|_{\boldsymbol{D}\vec{V}=\vec{b}} \overset{\mathrm{d}}{=} \gamma \mathbf{1}_n + \vec{J}|_{\boldsymbol{D}\vec{J}=\vec{d}}.$$

Then, we get the desired result by applying Lemma 11 in (34) on the second term on the right-hand side. □

The next Lemma applies the result of Lemma 1 to obtain the conditional distribution of the fixed interference matrix $\mathbf{A}$ given $\mathcal{G}_t$.

Lemma 2 *Fix t and assume that $\boldsymbol{Q}_t$ is a full-row rank matrix. Then, for the conditional distribution of the fixed interference matrix $\mathbf{A}$ given $\mathbf{A}\boldsymbol{Q}_t = \boldsymbol{R}_t$, we have*

$$\mathbf{A}|_{\mathbf{A}\boldsymbol{Q}_t=\boldsymbol{R}_t} \overset{\mathrm{d}}{=} \frac{\mu}{N}\mathbf{1}_{N\times N} + \bar{\boldsymbol{R}}_t \left(\boldsymbol{Q}_t^\top \boldsymbol{Q}_t\right)^{-1} \boldsymbol{Q}_t^\top + \widetilde{\mathbf{A}}_0 P^\perp. \qquad [42]$$

where $\widetilde{\mathbf{A}}_0 \overset{\mathrm{d}}{=} \mathbf{A} - \frac{\mu}{N}\mathbf{1}_{N\times N}$ independent of $\mathbf{A}$ and $P^\perp = (\mathbf{I} - P)$ that P denotes the orthogonal projector onto the column space of $\boldsymbol{Q}_t$ and

$$\bar{\boldsymbol{R}}_t = \boldsymbol{R}_t - \frac{\mu}{N}\mathbf{1}_{N\times N}\boldsymbol{Q}_t \qquad [43]$$

Proof. To calculate the conditional distribution of $\mathbf{A}$ given $\mathbf{A}\boldsymbol{Q}_t = \boldsymbol{R}_t$, we proceed by rewriting the interference matrix $\mathbf{A}$ as follows:

$$\mathbf{A} \overset{\mathrm{d}}{=} \frac{\mu}{N}\mathbf{1}_{N\times N} + \bar{\mathbf{A}},$$

where $\bar{\mathbf{A}}$ is a matrix of i.i.d. Gaussian entries with zero mean and variance σ^2. Therefore, we rewrite Eq. (40) as:

$$\bar{\mathbf{A}}\boldsymbol{Q}_t = \bar{\boldsymbol{R}}_t.$$

Following Lemma 1, we first solve the least square problem below:

$$\boldsymbol{E}_t = \mathrm{argmin}_{\bar{\mathbf{A}}} \left\{ \left\|\bar{\mathbf{A}}\right\|_F^2 : \bar{\mathbf{A}}\boldsymbol{Q}_t = \bar{\boldsymbol{R}}_t \right\}.$$

We write the Lagrangian

$$\left\|\bar{\mathbf{A}}\right\|_F^2 + \mathrm{Tr}\left(\boldsymbol{\Lambda}\left(\bar{\boldsymbol{R}}_t - \bar{\mathbf{A}}\boldsymbol{Q}_t\right)^\top\right),$$

where $\boldsymbol{\Lambda} \in \mathbb{R}^{N\times t}$ is the Lagrange multiplier. We get $2\bar{\mathbf{A}} = \boldsymbol{\Lambda}\boldsymbol{Q}_t^\top$, that implies

$$\boldsymbol{E}_t = \bar{\boldsymbol{R}}_t \left(\boldsymbol{Q}_t^\top \boldsymbol{Q}_t\right)^{-1} \boldsymbol{Q}_t^\top.$$

Next, we show that the orthogonal projection of $\widetilde{\mathbf{A}}_0$ onto the subspace $\mathcal{A} := \{\tilde{\mathbf{A}} : \tilde{\mathbf{A}}\boldsymbol{Q}_t = 0\}$ is equal to $\widetilde{\mathbf{A}}_0\,(\mathbf{I} - P)$. For that purpose, we follow the same steps as the proof of Lemma 10 in (34). First, note that by definition $\widetilde{\mathbf{A}}_0\,(\mathbf{I} - P)\,\boldsymbol{Q}_t = 0$; that is, $\widetilde{\mathbf{A}}_0\,(\mathbf{I} - P) \in \mathcal{A}$. Second, the orthogonal projection of $\widetilde{\mathbf{A}}_0\,(\mathbf{I} - P)$ onto the subspace $\mathcal{A}$ is equal to itself. That is,

$$\widetilde{\mathbf{A}}_0\,(\mathbf{I} - P)\,(\mathbf{I} - P) = \widetilde{\mathbf{A}}_0\,(\mathbf{I} - P) - \bar{\mathbf{A}}P + \bar{\mathbf{A}}PP = \widetilde{\mathbf{A}}_0\,(\mathbf{I} - P)\,.$$

Third, we show that if $\mathbf{A}' \in \mathcal{A}$, then $\mathbf{A}'(\mathbf{I} - P) = \mathbf{A}'$. To this end, note that if $\mathbf{A}' \in \mathcal{A}$, we have $\mathbf{A}'\boldsymbol{Q}_t = 0$; then, all the rows of the matrix $\mathbf{A}'$ are perpendicular to the columns of $\boldsymbol{Q}_t$. This implies that $\mathbf{A}'P = 0$ and so $\mathbf{A}'\,(\mathbf{I} - P) = \mathbf{A}'$. Finally, we need to show that the operator corresponding to this projection is symmetric. That is, for all matrices $\boldsymbol{C}$ and $\boldsymbol{D}$ it holds that $\mathrm{Tr}\left(\boldsymbol{C}\,(\mathbf{I} - P)\,\boldsymbol{D}^\top\right) = \mathrm{Tr}\left(\boldsymbol{D}\,(\mathbf{I} - P)\,\boldsymbol{C}^\top\right)$. We have,

$$\mathrm{Tr}\left(\boldsymbol{C}\,(\mathbf{I} - P)\,\boldsymbol{D}^\top\right) = \mathrm{Tr}\left(\left(\boldsymbol{C}\,(\mathbf{I} - P)\,\boldsymbol{D}^\top\right)^\top\right) = \mathrm{Tr}\left(\boldsymbol{D}\,(\mathbf{I} - P)\,\boldsymbol{C}^\top\right).$$

Applying Lemma 1 concludes the proof. □

Next lemma expresses the distribution of $\vec{Y}_{t+1}$ conditioning on the σ-algebra $\mathcal{G}_t$ or equivalently on the event $\mathbf{A}\boldsymbol{Q}_t = \boldsymbol{R}_t$.

Lemma 3 *Fix t and assume that $\boldsymbol{Q}_t$ is a full-row rank matrix. The following holds for the conditional distribution of the outcome vector $\vec{Y}_{t+1}$:*

$$\vec{Y}_{t+1}\Big|_{\mathcal{G}_t} \overset{\mathrm{d}}{=} \widetilde{\mathbf{A}}\vec{U}_t^{\perp} + \boldsymbol{R}_t\vec{\alpha}_t + \mathbf{B}_t\vec{U}_t + \vec{\epsilon}_t, \tag{44}$$

where the matrix $\widetilde{\mathbf{A}}$ is independent of $\mathbf{A}$ and has the same distribution.

Proof. By Eq. (1), we have

$$\vec{Y}_{t+1}\Big|_{\mathcal{G}_t} \overset{\mathrm{d}}{=} \left(\mathbf{A}\vec{U}_t + \mathbf{B}_t\vec{U}_t + \vec{\epsilon}_t\right)\Big|_{\mathcal{G}_t} \overset{\mathrm{d}}{=} \mathbf{A}\Big|_{\mathcal{G}_t}\vec{U}_t + \mathbf{B}_t\vec{U}_t + \vec{\epsilon}_t, \tag{45}$$

where we used the fact that $\mathbf{B}_t$ and $\vec{\epsilon}_t$ are independent of $\mathcal{G}_t$. Further, note that in the right-hand side of Eq. (45), the matrix $\mathbf{B}_t$ and the vector $\vec{\epsilon}_t$ are random objects while $\vec{U}_t$ is a deterministic known vector according to $\mathcal{G}_t$. Now, we use the result of Lemma 2. We get

$$\vec{Y}_{t+1}\Big|_{\mathcal{G}_t} \overset{\mathrm{d}}{=} \frac{\mu}{N}\mathbf{1}_{N\times N}\vec{U}_t + \bar{\boldsymbol{R}}_t\left(\boldsymbol{Q}_t^\top\boldsymbol{Q}_t\right)^{-1}\boldsymbol{Q}_t^\top\vec{U}_t + \widetilde{\mathbf{A}}_0P^{\perp}\vec{U}_t + \mathbf{B}_t\vec{U}_t + \vec{\epsilon}_t. \tag{46}$$

By $\vec{U}_t = \vec{U}_t^{\parallel} + \vec{U}_t^{\perp}$ and the fact that $\boldsymbol{Q}_t^\top\vec{U}_t^{\perp} = 0$ and using Eq. (43), we can write

$$\bar{\boldsymbol{R}}_t\left(\boldsymbol{Q}_t^\top\boldsymbol{Q}_t\right)^{-1}\boldsymbol{Q}_t^\top\vec{U}_t = \left(\boldsymbol{R}_t - \frac{\mu}{N}\mathbf{1}_{N\times N}\boldsymbol{Q}_t\right)\left(\boldsymbol{Q}_t^\top\boldsymbol{Q}_t\right)^{-1}\boldsymbol{Q}_t^\top\vec{U}_t^{\parallel} = \boldsymbol{R}_t\vec{\alpha}_t - \frac{\mu}{N}\mathbf{1}_{N\times N}\boldsymbol{Q}_t\vec{\alpha}_t. \tag{47}$$

where in the last equality we used $\vec{U}_t^{\parallel} = \boldsymbol{Q}_t\vec{\alpha}_t$. Considering Eq. (46) and Eq. (47) together, we have

$$\begin{aligned}\vec{Y}_{t+1}\Big|_{\mathcal{G}_t} &\overset{\mathrm{d}}{=} \frac{\mu}{N}\mathbf{1}_{N\times N}\vec{U}_t^{\parallel} + \frac{\mu}{N}\mathbf{1}_{N\times N}\vec{U}_t^{\perp} + \boldsymbol{R}_t\vec{\alpha}_t - \frac{\mu}{N}\mathbf{1}_{N\times N}\boldsymbol{Q}_t\vec{\alpha}_t + \widetilde{\mathbf{A}}_0P^{\perp}\vec{U}_t + \mathbf{B}_t\vec{U}_t + \vec{\epsilon}_t\\ &\overset{\mathrm{d}}{=} \frac{\mu}{N}\mathbf{1}_{N\times N}\vec{U}_t^{\perp} + \boldsymbol{R}_t\vec{\alpha}_t + \widetilde{\mathbf{A}}_0\vec{U}_t^{\perp} + \mathbf{B}_t\vec{U}_t + \vec{\epsilon}_t\\ &\overset{\mathrm{d}}{=} \widetilde{\mathbf{A}}\vec{U}_t^{\perp} + \boldsymbol{R}_t\vec{\alpha}_t + \mathbf{B}_t\vec{U}_t + \vec{\epsilon}_t,\end{aligned}$$

where in the last equality we used $\widetilde{\mathbf{A}} \overset{\mathrm{d}}{=} \frac{\mu}{N}\mathbf{1}_{N\times N} + \widetilde{\mathbf{A}}_0$ that concludes the proof. □

11.4. Proof of Theorem 7. For each t, we can assume, without loss of generality, that the function $Y \mapsto g_t(Y, \vec{W}, \vec{X})$ is non-constant with a positive probability with respect to $(\vec{W}, \vec{X}) \sim \Pi \times p_{\boldsymbol{X}}$. The case where $Y \mapsto g_t(Y, \vec{W}, \vec{X})$ is almost surely constant is trivial and does not require further analysis. We use induction on t.

Step 1. Let $t = 0$ and note that $\boldsymbol{Q}_0$ and $\boldsymbol{R}_0$ are empty matrices and the σ-algebra $\mathcal{G}_0$ is generated by $\vec{Y}_0$, $\boldsymbol{W}$, and $\boldsymbol{X}$. We prove Parts (a) and (b) for $t = 0$ and Part (c) for $t = 1$ as the base case.

(a) By Assumption 1 and Eq. (44), conditioning on the values of $\vec{Y}_0$, $\boldsymbol{W}$, as well as $\boldsymbol{X}$ and so on the value of $\vec{U}_0 = g_0\left(\vec{Y}_0, \boldsymbol{W}, \boldsymbol{X}\right)$, the elements of $\vec{Y}_1$ are i.i.d. Gaussian random variables with mean ν_{1N} and variance ρ_{1N}^2:

$$\begin{aligned}\nu_{1N} &:= \mathbb{E}\left[Y_1^n\Big|\vec{U}_0\right] = \mathbb{E}\left[[\mathbf{A}\vec{U}_0 + \mathbf{B}_0\vec{U}_0 + \vec{\epsilon}_0]_n\Big|\vec{U}_0\right] = \frac{\mu + \mu_0}{N}\sum_{n=1}^{N}U_0^n,\\ \rho_{1N}^2 &:= \mathrm{Var}\left[Y_1^n\Big|\vec{U}_0\right] = \frac{\sigma^2 + \sigma_0^2}{N}\sum_{n=1}^{N}(U_0^n)^2 + \sigma_e^2,\end{aligned} \tag{48}$$

where $U_0^n = g_0\big(Y_0^n, \vec{W}^n, \vec{X}^n\big)$ is the nth element of the column vector $\vec{U}_0$. By Assumption 3-(iv), both ν_{1N} and ρ_{1N}^2 are bounded. Now, if we let Z be a standard Normal random variable, by inequality $(a+b)^l \le 2^{l-1}(a^l + b^l)$, for $l \ge 1$, that is a straightforward result of Jensen's inequality, we get

$$\mathbb{E}\left[\left|Y_1^n\right|^l \middle| \vec{U}_0\right] = \mathbb{E}\left[\left|\nu_{1N} + \rho_{1N} Z\right|^l \middle| \vec{U}_0\right] \le 2^{l-1}\mathbb{E}\left[\left|\nu_{1N}\right|^l + \left|\rho_{1N}\right|^l \left|Z\right|^l \middle| \vec{U}_0\right] \le c. \tag{49}$$

Here, c is a constant independent of N and might alter in different lines. Reusing Jensen's inequality multiple times and the fact that $\psi \in \mathcal{CP}(k)$, for $\kappa > 0$, we get

$$\begin{aligned}
&\mathbb{E}\left[\left|\psi\big(Y_1^n, \vec{W}^n, \vec{X}^n\big) - \mathbb{E}_{\mathbf{G}_0,\vec{\epsilon}_0}\left[\psi\big(Y_1^n, \vec{W}^n, \vec{X}^n\big)\right]\right|^{2+\kappa}\right] \\
&\le \mathbb{E}\left[\left(\left|\psi\big(Y_1^n, \vec{W}^n, \vec{X}^n\big)\right| + \mathbb{E}_{\mathbf{G}_0,\vec{\epsilon}_0}\left[\left|\psi\big(Y_1^n, \vec{W}^n, \vec{X}^n\big)\right|\right]\right)^{2+\kappa}\right] \\
&\le \mathbb{E}\left[2^{1+\kappa}\left(\left|\psi\big(Y_1^n, \vec{W}^n, \vec{X}^n\big)\right|^{2+\kappa} + \mathbb{E}_{\mathbf{G}_0,\vec{\epsilon}_0}\left[\left|\psi\big(Y_1^n, \vec{W}^n, \vec{X}^n\big)\right|^{2+\kappa}\right]\right)\right] \\
&\le c\mathbb{E}\left[\left(1 + \left\|\big(Y_1^n, \vec{W}^n, \vec{X}^n\big)\right\|^k\right)^{2+\kappa} + \mathbb{E}_{\mathbf{G}_0,\vec{\epsilon}_0}\left[\left(1 + \left\|\big(Y_1^n, \vec{W}^n, \vec{X}^n\big)\right\|^k\right)^{2+\kappa}\right]\right] \\
&\le c\mathbb{E}\left[2 + \left(\big(Y_1^n\big)^2 + \left\|\big(\vec{W}^n, \vec{X}^n\big)\right\|^2\right)^{k+\frac{k\kappa}{2}} + \mathbb{E}_{\mathbf{G}_0,\vec{\epsilon}_0}\left[\left(\big(Y_1^n\big)^2 + \left\|\big(\vec{W}^n, \vec{X}^n\big)\right\|^2\right)^{k+\frac{k\kappa}{2}}\right]\right] \\
&\le c\mathbb{E}\left[2 + \big(Y_1^n\big)^{2k+k\kappa} + \left\|\big(\vec{W}^n, \vec{X}^n\big)\right\|^{2k+k\kappa} + \mathbb{E}_{\mathbf{G}_0,\vec{\epsilon}_0}\left[\big(Y_1^n\big)^{2k+k\kappa} + \left\|\big(\vec{W}^n, \vec{X}^n\big)\right\|^{2k+k\kappa}\right]\right],
\end{aligned} \tag{50}$$

where $\mathbb{E}_{\mathbf{G}_0,\vec{\epsilon}_0}$ is the expectation with respect to the randomness of the interference matrix $\mathbf{G}_0 = \mathbf{A} + \mathbf{B}_0$ and observation noise $\vec{\epsilon}_0$. In Eq. (50), because $k \ge 2$ and $\kappa > 0$, we get $2 + \kappa \ge 1$ and $k + \frac{k\kappa}{2} \ge 1$, and we are allowed to use the inequality $(v_1 + v_2)^l \le 2^{l-1}(v_1^l + v_2^l)$, $v_1, v_2 \ge 0$. Therefore, by Eq. (49), we have

$$\frac{1}{N}\sum_{n=1}^{N}\mathbb{E}\left[\left|\psi\big(Y_1^n, \vec{W}^n, \vec{X}^n\big) - \mathbb{E}_{\mathbf{G}_0,\vec{\epsilon}_0}\left[\psi\big(Y_1^n, \vec{W}^n, \vec{X}^n\big)\right]\right|^{2+\kappa}\right] \le cN^{\kappa/2},$$

where c is a constant independent of N. Applying the Strong Law of Large Numbers (SLLN) for triangular arrays in Theorem 8, we obtain

$$\lim_{N\to\infty}\frac{1}{N}\sum_{n=1}^{N}\left(\psi\big(Y_1^n, \vec{W}^n, \vec{X}^n\big) - \mathbb{E}_{\mathbf{G}_0,\vec{\epsilon}_0}\left[\psi\big(Y_1^n, \vec{W}^n, \vec{X}^n\big)\right]\right) \overset{\text{a.s.}}{=} 0. \tag{51}$$

By Assumption 3-(iv) and Eq. (48), we can write

$$\begin{aligned}
\lim_{N\to\infty}\nu_{1N} &= \lim_{N\to\infty}\frac{\mu + \mu_0}{N}\sum_{n=1}^{N} g_0\big(Y_0^n, \vec{W}^n, \vec{X}^n\big) = \nu_1 \\
\lim_{N\to\infty}\rho_{1N}^2 &= \lim_{N\to\infty}\frac{\sigma^2 + \sigma_0^2}{N}\sum_{n=1}^{N} g_0\big(Y_0^n, \vec{W}^n, \vec{X}^n\big)^2 + \sigma_e^2 = \rho_1^2.
\end{aligned}$$

Now, we use Theorem 9 for $f(\vec{W}^n, \vec{X}^n) = \mathbb{E}_{\mathbf{G}_0,\vec{\epsilon}_0}\left[\psi\big(Y_1^n, \vec{W}^n, \vec{X}^n\big)\right]$. By Eq. (51), we can write

$$\begin{aligned}
\lim_{N\to\infty}\frac{1}{N}\sum_{n=1}^{N}\psi\big(Y_1^n, \vec{W}^n, \vec{X}^n\big) &\overset{\text{a.s.}}{=} \lim_{N\to\infty}\frac{1}{N}\sum_{n=1}^{N}\mathbb{E}_{\mathbf{G}_0,\vec{\epsilon}_0}\left[\psi\big(Y_1^n, \vec{W}^n, \vec{X}^n\big)\right] \\
&\overset{\text{a.s.}}{=} \lim_{N\to\infty}\mathbb{E}\left[\psi\big(\nu_{1N} + \rho_{1N}Z, \vec{W}, \vec{X}\big)\right] \overset{\text{a.s.}}{=} \mathbb{E}\left[\psi\big(\nu_1 + \rho_1 Z, \vec{W}, \vec{X}\big)\right].
\end{aligned} \tag{52}$$

Note that $\mathbb{E}_{\mathbf{G}_0,\vec{\epsilon}_0}\left[\psi\big(Y_1^n, \vec{W}^n, \vec{X}^n\big)\right] \in \mathcal{CP}(k)$, since $\psi \in \mathcal{CP}(k)$. In the last equality in Eq. (52), we used the Dominated Convergence Theorem (DCT), see e.g., Theorem 16.4 in (35), which allows us to interchange the limit and the expectation. Additionally, we utilized the continuous mapping theorem, stated in Theorem 2.3 in (36), to pass the limit through the function. It is important to note that Z is independent of both $\vec{W}$ and $\vec{X}$ since its randomness arises from the interference matrix $\mathbf{G}_0$ and observation noise $\vec{\epsilon}_0$, which are independent of $\vec{W}$ and $\vec{X}$.

In the second step of the induction, we need two more results. The first result is given in Eq. (53) and we can derive it by following the same procedure as above.

$$\lim_{N\to\infty}\frac{1}{N}\sum_{n=1}^{N}\psi\big(Y_1^n, Y_1^n - \mathrm{B}_0^{n:}\vec{U}_0 - \epsilon_0^n, \vec{W}^n, \vec{X}^n\big) \overset{\text{a.s.}}{=} \mathbb{E}\left[\psi\big(\nu_1 + \rho_1 Z, \bar{\nu}_1 + \bar{\rho}_1 Z', \vec{W}, \vec{X}\big)\right], \tag{53}$$

where we assume that $\psi : \mathbb{R}^{2+T+M} \mapsto \mathbb{R}$ is a $\mathcal{CP}(k)$ function and $\mathrm{B}_0^{n\cdot}$ is the nth row the matrix $\mathbf{B}_0$. For the second result, consider the function $g_0\big(Y_0^n, \vec{W}^n, \vec{X}^n\big)\phi\big(Y_1^n, Y_1^n - \mathrm{B}_0^{n\cdot}\vec{U}_0 - \epsilon_0^n, \vec{W}^n, \vec{X}^n\big)$ that $\phi \in \mathcal{CP}(\frac{k}{2})$ is arbitrary. By Assumption 3-(i), we know that this function lies within $\mathcal{CP}(k)$. Hence, following the same procedure as in Eq. (50), we can check the conditions of Theorem 8. This implies,

$$\begin{aligned}&\lim_{N\to\infty}\frac{1}{N}\sum_{n=1}^{N}\Big(g_0\big(Y_0^n, \vec{W}^n, \vec{X}^n\big)\phi\big(Y_1^n, Y_1^n - \mathrm{B}_0^{n\cdot}\vec{U}_0 - \epsilon_0^n, \vec{W}^n, \vec{X}^n\big)\Big)\\ &\overset{\text{a.s.}}{=}\lim_{N\to\infty}\frac{1}{N}\sum_{n=1}^{N}\Big(\mathbb{E}_{\mathbf{G}_0,\vec{\epsilon}_0}\left[g_0\big(Y_0^n, \vec{W}^n, \vec{X}^n\big)\phi\big(Y_1^n, Y_1^n - \mathrm{B}_0^{n\cdot}\vec{U}_0 - \epsilon_0^n, \vec{W}^n, \vec{X}^n\big)\right]\Big).\end{aligned} \quad [54]$$

Note that in Eq. (54), the expectation is with respect to the randomness of the interference matrix and observation noise. Thus, letting $\varphi(\vec{W}^n, \vec{X}^n) = \mathbb{E}_{\mathbf{G}_0,\vec{\epsilon}_0}\left[\phi\big(Y_1^n, Y_1^n - \mathrm{B}_0^{n\cdot}\vec{U}_0 - \epsilon_0^n, \vec{W}^n, \vec{X}^n\big)\right]$, by Assumption 3-(v) and the DCT, we have

$$\begin{aligned}&\lim_{N\to\infty}\frac{1}{N}\sum_{n=1}^{N}\Big(g_0\big(Y_0^n, \vec{W}^n, \vec{X}^n\big)\phi\big(Y_1^n, Y_1^n - \mathrm{B}_0^{n\cdot}\vec{U}_0 - \epsilon_0^n, \vec{W}^n, \vec{X}^n\big)\Big)\\ &\overset{\text{a.s.}}{=}\lim_{N\to\infty}\frac{1}{N}\sum_{n=1}^{N}\Big(\mathbb{E}_{\mathbf{G}_0,\vec{\epsilon}_0}\left[g_0\big(Y_0^n, \vec{W}^n, \vec{X}^n\big)\phi\big(Y_1^n, Y_1^n - \mathrm{B}_0^{n\cdot}\vec{U}_0 - \epsilon_0^n, \vec{W}^n, \vec{X}^n\big)\right]\Big)\\ &\overset{\text{a.s.}}{=}\lim_{N\to\infty}\mathbb{E}\big[\bar{g}_0(\vec{W}, \vec{X})\phi(\nu_{1N} + \rho_{1N}Z, \bar{\nu}_{1N} + \bar{\rho}_{1N}Z', \vec{W}, \vec{X})\big].\end{aligned}$$

Similar to Eq. (52), we obtain the desired result as follows

$$\begin{aligned}&\lim_{N\to\infty}\frac{1}{N}\sum_{n=1}^{N}\Big(g_0\big(Y_0^n, \vec{W}^n, \vec{X}^n\big)\phi\big(Y_1^n, Y_1^n - \mathrm{B}_0^{n\cdot}\vec{U}_0 - \epsilon_0^n, \vec{W}^n, \vec{X}^n\big)\Big)\\ &\overset{\text{a.s.}}{=}\mathbb{E}\big[\bar{g}_0(\vec{W}, \vec{X})\phi\big(\nu_1 + \rho_1 Z, \bar{\nu}_1 + \bar{\rho}_1 Z', \vec{W}, \vec{X}\big)\big].\end{aligned}$$

(b) By Assumption 3-(iv) as well as Eq. (52) for $\psi(y, \cdot, \cdot) = y$ and $\psi(y, \cdot, \cdot) = y^2$, we have

$$\begin{aligned}\lim_{N\to\infty}\frac{1}{N}\sum_{n=1}^{N}Y_1^n &\overset{\text{a.s.}}{=} \nu_1 = \lim_{N\to\infty}\frac{\mu+\mu_0}{N}\sum_{n=1}^{N}U_0^n,\\ \lim_{N\to\infty}\frac{1}{N}\sum_{n=1}^{N}\big(Y_1^n\big)^2 &\overset{\text{a.s.}}{=} \nu_1^2 + \rho_1^2 = \nu_1^2 + \lim_{N\to\infty}\frac{\sigma^2+\sigma_0^2}{N}\sum_{n=1}^{N}(U_0^n)^2 + \sigma_e^2.\end{aligned} \quad [55]$$

Likewise, by Assumption 3-(iv) as well as Eq. (53) for functions $\psi(y, \bar{y}, \cdot, \cdot) = \bar{y}$ and $\psi(y, \bar{y}, \cdot, \cdot) = \bar{y}^2$, we have

$$\begin{aligned}\lim_{N\to\infty}\frac{1}{N}\sum_{n=1}^{N}\big(Y_1^n - \mathrm{B}_0^{n\cdot}\vec{U}_0 - \epsilon_0^n\big) &\overset{\text{a.s.}}{=} \bar{\nu}_1 = \lim_{N\to\infty}\frac{\mu}{N}\sum_{n=1}^{N}U_0^n,\\ \lim_{N\to\infty}\frac{1}{N}\sum_{n=1}^{N}\big(Y_1^n - \mathrm{B}_0^{n\cdot}\vec{U}_0 - \epsilon_0^n\big)\big(Y_1^n - \mathrm{B}_0^{n\cdot}\vec{U}_0 - \epsilon_0^n\big) &\overset{\text{a.s.}}{=} \bar{\nu}_1^2 + \lim_{N\to\infty}\frac{\sigma^2}{N}\sum_{n=1}^{N}U_0^{n\,2}.\end{aligned}$$

Finally, by applying Theorem 8 and considering the fact that elements of $\vec{\epsilon}_0$ are zero-mean random variables independent of everything, we can write

$$\begin{aligned}\lim_{N\to\infty}\frac{1}{N}\sum_{n=1}^{N}Y_1^n\big(Y_1^n - \mathrm{B}_0^{n\cdot}\vec{U}_0 - \epsilon_0^n\big) &= \lim_{N\to\infty}\frac{1}{N}\sum_{n=1}^{N}\Big(\big(\mathrm{A}^{n\cdot} + \mathrm{B}_0^{n\cdot}\big)\vec{U}_0 + \epsilon_0^n\Big)(\mathrm{A}^{n\cdot}\vec{U}_0)\\ &\overset{\text{a.s.}}{=} \lim_{N\to\infty}\frac{1}{N}\sum_{n=1}^{N}(\mathrm{A}^{n\cdot}\vec{U}_0)^2 + \lim_{N\to\infty}\frac{1}{N}\sum_{n=1}^{N}(\mathrm{A}^{n\cdot}\vec{U}_0)(\mathrm{B}_0^{n\cdot}\vec{U}_0),\end{aligned}$$

where $\mathrm{A}^{n\cdot}$ and $\mathrm{B}_0^{n\cdot}$ denote row n of $\mathbf{A}$ and $\mathbf{B}_0$, respectively. Note that for all $n \in [N]$, random variables $\mathrm{A}^{n\cdot}\vec{U}_0$ and $\mathrm{B}_0^{n\cdot}\vec{U}_0$ are i.i.d. and

$$\mathrm{A}^{n\cdot}\vec{U}_0 \sim \mathcal{N}\left(\frac{\mu}{N}\sum_{i=1}^{N}U_0^i, \frac{\sigma^2}{N}\sum_{i=1}^{N}(U_0^i)^2\right), \qquad \mathrm{B}_0^{n\cdot}\vec{U}_0 \sim \mathcal{N}\left(\frac{\mu_0}{N}\sum_{i=1}^{N}U_0^i, \frac{\sigma_0^2}{N}\sum_{i=1}^{N}(U_0^i)^2\right).$$

Therefore, we get

$$\lim_{N\to\infty}\frac{1}{N}\sum_{n=1}^{N} Y_1^n\left(Y_1^n - \mathrm{B}_0^{n\cdot}\vec{U}_0 - \epsilon_0^n\right) \overset{\text{a.s.}}{=} \lim_{N\to\infty}\frac{\mu(\mu+\mu_0)}{N}\left(\sum_{i=1}^{N} U_0^i\right)^2 + \lim_{N\to\infty}\frac{\sigma^2}{N}\sum_{i=1}^{N}(U_0^i)^2,$$

where the limits exist based on Assumption 3-(iv).

(c) For $t=1$, the matrix $\boldsymbol{Q}_1$ is equal to the vector $\vec{U}_0$ and $\boldsymbol{V}_1$ is equal to the vector $\vec{Y}_1$. By Assumption 3-(iv), we have

$$\lim_{N\to\infty}\frac{\boldsymbol{Q}_1^\top\boldsymbol{Q}_1}{N} = \lim_{N\to\infty}\left\langle\vec{U}_0,\vec{U}_0\right\rangle = \lim_{N\to\infty}\frac{1}{N}\sum_{i=1}^{N}(U_0^n)^2 > 0,$$

as well as

$$\lim_{N\to\infty}\frac{\boldsymbol{V}_1^\top\boldsymbol{V}_1}{N} - \lim_{N\to\infty}\frac{\boldsymbol{V}_1^\top\mathbf{1}_{N\times 1}}{N}\lim_{N\to\infty}\frac{\mathbf{1}_{1\times N}\boldsymbol{V}_1}{N} =$$
$$\lim_{N\to\infty}\left\langle\vec{Y}_1,\vec{Y}_1\right\rangle - \lim_{N\to\infty}\frac{\vec{Y}_1^\top\mathbf{1}_{N\times 1}}{N}\lim_{N\to\infty}\frac{\mathbf{1}_{1\times N}\vec{Y}_1}{N} \overset{\text{a.s.}}{=} \rho_1^2 > 0,$$

where we also used the result of Step 1-(b).

Step 2. Assuming that Eq. (37), Eq. (56), and Eq. (57) hold for $s=0,1,\dots,t-1$, Eq. (38) for $0\le r,s<t$, and Eq. (39) for $s=1,\dots,t-1$, we show that they also hold for t.

$$\begin{aligned}&\lim_{N\to\infty}\frac{1}{N}\sum_{n=1}^{N}\psi\left(Y_1^n, Y_1^n - \mathrm{B}_0^{n\cdot}\vec{U}_0 - \epsilon_0^n,\dots,Y_{s+1}^n, Y_{s+1}^n - \mathrm{B}_s^{n\cdot}\vec{U}_s - \epsilon_s^n, \vec{W}^n, \vec{X}^n\right)\\ &\overset{\text{a.s.}}{=} \mathbb{E}\Big[\psi\big(\nu_1+\rho_1 Z_1, \bar{\nu}_1+\bar{\rho}_1 Z_1', \dots, \nu_{s+1}+\rho_{s+1}Z_{s+1}, \bar{\nu}_{s+1}+\bar{\rho}_{s+1}Z_{s+1}', \vec{W}, \vec{X}\big)\Big],\end{aligned} \tag{56}$$

where $\psi:\mathbb{R}^{2(s+1)+T+M}\mapsto\mathbb{R}$ is within $\mathcal{CP}(k)$. Further, we write

$$\begin{aligned}&\lim_{N\to\infty}\frac{1}{N}\sum_{n=1}^{N}\Big(g_0\left(Y_0^n,\vec{W}^n,\vec{X}^n\right)\phi\big(Y_1^n, Y_1^n - \mathrm{B}_0^{n\cdot}\vec{U}_0 - \epsilon_0^n,\dots,\\ &\qquad\qquad Y_{s+1}^n, Y_{s+1}^n - \mathrm{B}_s^{n\cdot}\vec{U}_s - \epsilon_s^n, \vec{W}^n, \vec{X}^n\big)\Big)\\ &\overset{\text{a.s.}}{=} \mathbb{E}\Big[\bar{g}_0(\vec{W},\vec{X})\phi\big(\nu_1+\rho_1 Z_1, \bar{\nu}_1+\bar{\rho}_1 Z_1', \dots, \nu_{s+1}+\rho_{s+1}Z_{s+1}, \bar{\nu}_{s+1}+\bar{\rho}_{s+1}Z_{s+1}', \vec{W}, \vec{X}\big)\Big],\end{aligned} \tag{57}$$

where $\phi:\mathbb{R}^{2(s+1)+T+M}\mapsto\mathbb{R}$ is within $\mathcal{CP}(\frac{k}{2})$. Below, we first prove Eq. (39).

(c) Defining the function $\psi = g_s\left(Y_s^n,\vec{W}^n,\vec{X}^n\right)g_r\left(Y_r^n,\vec{W}^n,\vec{X}^n\right)$, by the induction hypothesis (a), for $1\le r,s\le t$, we have

$$\begin{aligned}\lim_{N\to\infty}\frac{1}{N}\sum_{n=1}^{N}U_0^nU_s^n &= \lim_{N\to\infty}\left\langle\vec{U}_0,\vec{U}_s\right\rangle \overset{\text{a.s.}}{=} \mathbb{E}\left[\bar{g}_0(\vec{W},\vec{X})g_s\left(\nu_s+\rho_s Z_s,\vec{W},\vec{X}\right)\right]\\ \lim_{N\to\infty}\frac{1}{N}\sum_{n=1}^{N}U_s^nU_r^n &= \lim_{N\to\infty}\left\langle\vec{U}_s,\vec{U}_r\right\rangle \overset{\text{a.s.}}{=} \mathbb{E}\left[g_s\left(\nu_s+\rho_s Z_s,\vec{W},\vec{X}\right)g_r\left(\nu_r+\rho_r Z_r,\vec{W},\vec{X}\right)\right].\end{aligned} \tag{58}$$

Now, let $\vec{u}=(u_1,\dots,u_t)^\top\in\mathbb{R}^t$ be a non-zero vector. By Eq. (58), we have

$$\begin{aligned}\vec{u}^\top\left(\lim_{N\to\infty}\frac{\boldsymbol{Q}_t^\top\boldsymbol{Q}_t}{N}\right)\vec{u} &= \lim_{N\to\infty}\vec{u}^\top\frac{\boldsymbol{Q}_t^\top\boldsymbol{Q}_t}{N}\vec{u}\\ &\overset{\text{a.s.}}{=} \mathbb{E}\left[\left(u_1\bar{g}_0(\vec{W},\vec{X}) + \sum_{s=2}^{t}u_s g_{s-1}\left(\nu_{s-1}+\rho_{s-1}Z_{s-1},\vec{W},\vec{X}\right)\right)^2\right]\\ &\quad + u_1^2\left(\frac{\rho_1^2-\sigma_e^2}{\sigma^2+\sigma_0^2} - \mathbb{E}\left[\bar{g}_0(\vec{W},\vec{X})^2\right]\right).\end{aligned} \tag{59}$$

By Assumption 3-(v), the last term in Eq. (59) is non-negative. Now, if $u_1\neq 0 = u_2=\ldots=u_t$, then the result is immediate by Assumption 3-(iv). Otherwise, there is some $2\le i\le t$ such that $u_i\neq 0$. Recalling that $y\mapsto g_{i-1}(y,\vec{W},\vec{X})$ is a non-constant function with a positive probability with respect to $(\vec{W},\vec{X})\sim\pi\times p$, the mapping $(y_1,\dots,y_t)\mapsto$

$\sum_{s=1}^{t} u_s g_{s-1}\big(y_s, \vec{W}, \vec{X}\big)$ is a non-constant function. But, by the induction hypothesis (b), it is straightforward to show that

$$\mathrm{Cov}\left[(\nu_1+\rho_1 Z_1,\dots,\nu_{t-1}+\rho_{t-1}Z_{t-1})\right] \overset{\text{a.s.}}{=} \lim_{N\to\infty} \frac{V_{t-1}^\top V_{t-1}}{N} - \lim_{N\to\infty} \frac{V_{t-1}^\top \mathbf{1}_{N\times 1}}{N} \lim_{N\to\infty} \frac{\mathbf{1}_{1\times N} V_{t-1}}{N},$$

which is by the induction hypothesis positive definite. This implies that the random variable $u_1 \bar{g}_0(\vec{W},\vec{X}) + \sum_{s=2}^{t} u_s g_{s-1}\big(\nu_{s-1}+\rho_{s-1}Z_{s-1}, \vec{W}, \vec{X}\big)$ has a non-degenerate distribution. That means,

$$\forall \vec{u}\neq 0,\ \ \vec{u}^\top \left(\lim_{N\to\infty} \frac{\boldsymbol{Q}_t^\top \boldsymbol{Q}_t}{N}\right) \vec{u} > 0 \qquad \Longrightarrow \qquad \lim_{N\to\infty} \frac{\boldsymbol{Q}_t^\top \boldsymbol{Q}_t}{N} \succ 0.$$

For the second part, for $1 \le r, s \le t$, let us denote

$$v_{r,s} := \left[\frac{\boldsymbol{V}_t{}^\top \boldsymbol{V}_t}{N} - \frac{\boldsymbol{V}_t{}^\top \mathbf{1}_{N\times 1}}{N} \frac{\mathbf{1}_{1\times N}\boldsymbol{V}_t}{N}\right]_{r,s} = \frac{\vec{Y}_r{}^\top \vec{Y}_s}{N} - \frac{\vec{Y}_r{}^\top \mathbf{1}_{N\times 1}}{N} \frac{\mathbf{1}_{1\times N}\vec{Y}_s}{N}.$$

By the induction hypothesis (b), if $r \neq s$, we have

$$\lim_{N\to\infty} v_{r,s} \overset{\text{a.s.}}{=} \nu_r \nu_s + \lim_{N\to\infty} \frac{\sigma^2}{N} \sum_{n=1}^{N} U_{(r-1)n} U_{(s-1)n} - \nu_r\nu_s = \lim_{N\to\infty} \frac{\sigma^2}{N} \sum_{n=1}^{N} U_{(r-1)n} U_{(s-1)n},$$

and if $r = s$, we have

$$\lim_{N\to\infty} v_{r,r} \overset{\text{a.s.}}{=} \nu_r^2 + \lim_{N\to\infty} \frac{\sigma^2+\sigma_r^2}{N} \sum_{n=1}^{N} U_{(r-1)n} U_{(r-1)n} + \sigma_e^2 - \nu_r^2 = \lim_{N\to\infty} \frac{\sigma^2+\sigma_r^2}{N} \sum_{n=1}^{N} U_{(r-1)n}^2 + \sigma_e^2.$$

Then, the result is straightforward as $\boldsymbol{Q}_t$ is positive definite.

Corollary 3 *The vector $\vec{\alpha}$ defined in* Eq. (36) *has a finite limit as $N \to \infty$.*

Proof. By Eq. (36), we can write

$$\lim_{N\to\infty} \vec{\alpha}_t = \lim_{N\to\infty} \left(\boldsymbol{Q}_t^\top \boldsymbol{Q}_t\right)^{-1} \boldsymbol{Q}_t^\top \vec{U}_t = \lim_{N\to\infty} \left(\frac{\boldsymbol{Q}_t^\top \boldsymbol{Q}_t}{N}\right)^{-1} \lim_{N\to\infty} \frac{\boldsymbol{Q}_t^\top \vec{U}_t}{N}. \tag{60}$$

Using the result of part (c), for large values of N, the matrix $\frac{\boldsymbol{Q}_t^\top \boldsymbol{Q}_t}{N} \in \mathbb{R}^{t\times t}$ is positive definite (this is true because the eigenvalues of a matrix vary continuously with respect to its entries). Then, note that the mapping $\boldsymbol{M} \mapsto \boldsymbol{M}^{-1}$ is continuous for invertible matrices $\boldsymbol{M}$. As a result, we get

$$\lim_{N\to\infty} \left(\frac{\boldsymbol{Q}_t^\top \boldsymbol{Q}_t}{N}\right)^{-1} = \left(\lim_{N\to\infty} \frac{\boldsymbol{Q}_t^\top \boldsymbol{Q}_t}{N}\right)^{-1}.$$

Since the matrix $\lim_{N\to\infty} \frac{\boldsymbol{Q}_t^\top \boldsymbol{Q}_t}{N}$ is positive definite, the first term in the RHS of Eq. (60) is well-defined and finite. The finiteness of the other term is the consequence of Eq. (58). □

(b) Below, we first derive several minor results; then, we use them to show Eq. (38) holds true for $0 \le r, s \le t$. Here, we use the SSLN given in Theorem 8 multiple times without checking the conditions as they are straightforward.

Denote by $\widetilde{\mathrm{A}}^{n\cdot}$ and $\mathrm{B}_t^{n\cdot}$ the nth rows of $\widetilde{\mathbf{A}}$ and $\mathbf{B}_t$. Recalling Lemma 3, note that $\widetilde{\mathrm{A}}^{n\cdot}\vec{U}_t^\perp$ and $\mathrm{B}_t^{n\cdot}\vec{U}_t$ are Gaussian i.i.d. random variables:

$$\widetilde{\mathrm{A}}^{n\cdot}\vec{U}_t^\perp \sim \mathcal{N}\left(\frac{\mu}{N}\sum_{i=1}^{N} U_t^{\perp i}, \frac{\sigma^2}{N}\sum_{i=1}^{N} (U_t^{\perp i})^2\right), \qquad \mathrm{B}_t^{n\cdot}\vec{U}_t \sim \mathcal{N}\left(\frac{\mu_t}{N}\sum_{i=1}^{N} U_t^{i}, \frac{\sigma_t^2}{N}\sum_{i=1}^{N} (U_t^{i})^2\right), \tag{61}$$

where $U_t^n = g_t\big(Y_t^n, \vec{W}^n, \vec{X}^n\big)$ is the nth element of the vector $\vec{U}_t$ and $U_t^{\perp n}$ is the nth element of the vector $\vec{U}_t^\perp$. Applying Theorem 8, we get

$$\lim_{N\to\infty} \frac{1}{N}\sum_{n=1}^{N} \widetilde{\mathrm{A}}^{n\cdot}\vec{U}_t^\perp \overset{\text{a.s.}}{=} \lim_{N\to\infty} \frac{\mu}{N}\sum_{n=1}^{N} U_t^{\perp n}, \tag{62}$$

and similarly

$$\lim_{N\to\infty} \frac{1}{N}\sum_{n=1}^{N} \mathrm{B}_t^{n\cdot}\vec{U}_t \overset{\text{a.s.}}{=} \lim_{N\to\infty} \frac{\mu_t}{N}\sum_{n=1}^{N} U_t^{n}. \tag{63}$$

Similarly, Eq. (61) implies that:

$$\lim_{N\to\infty}\frac{1}{N}\sum_{n=1}^{N}\left(\widetilde{\mathrm{A}}^{n\cdot}\vec{U}_t^{\perp}\right)^2 \overset{\text{a.s.}}{=} \lim_{N\to\infty}\left(\frac{\mu}{N}\sum_{n=1}^{N}U_t^{\perp n}\right)^2 + \lim_{N\to\infty}\frac{\sigma^2}{N}\sum_{n=1}^{N}\left(U_t^{\perp n}\right)^2, \tag{64}$$

as well as

$$\lim_{N\to\infty}\frac{1}{N}\sum_{n=1}^{N}\left(\mathrm{B}_t^{n\cdot}\vec{U}_t\right)^2 \overset{\text{a.s.}}{=} \lim_{N\to\infty}\left(\frac{\mu_t}{N}\sum_{n=1}^{N}U_t^{n}\right)^2 + \lim_{N\to\infty}\frac{\sigma_t^2}{N}\sum_{n=1}^{N}\left(U_t^{n}\right)^2. \tag{65}$$

Next, by Eq. (34), the induction hypothesis and Theorem 8, we have

$$\begin{aligned}\lim_{N\to\infty}\frac{1}{N}\sum_{n=1}^{N}[\boldsymbol{R}_t\vec{\alpha}_t]_n &= \lim_{N\to\infty}\frac{1}{N}\sum_{n=1}^{N}\sum_{s=0}^{t-1}\alpha_s\left(Y_{s+1}^n - \mathrm{B}_s^{n\cdot}U_s^n - \epsilon_s^n\right)\\ &= \sum_{s=0}^{t-1}\alpha_s\left(\lim_{N\to\infty}\frac{1}{N}\sum_{n=1}^{N}\left(Y_{s+1}^n - \mathrm{B}_s^{n\cdot}U_s^n - \epsilon_s^n\right)\right)\\ &\overset{\text{a.s.}}{=} \sum_{s=0}^{t-1}\alpha_s\left(\lim_{N\to\infty}\frac{\mu}{N}\sum_{n=1}^{N}U_s^n\right)\\ &= \lim_{N\to\infty}\frac{\mu}{N}\sum_{n=1}^{N}U_t^{\parallel n},\end{aligned} \tag{66}$$

where in the last equality we used Eq. (35). Considering Eq. (34) and the induction hypothesis Eq. (38f), it yields

$$\begin{aligned}&\lim_{N\to\infty}\frac{1}{N}\sum_{n=1}^{N}\left([\boldsymbol{R}_t\vec{\alpha}_t]_n\right)^2\\ &= \lim_{N\to\infty}\frac{1}{N}\sum_{n=1}^{N}\left(\sum_{s=0}^{t-1}\alpha_s\left(Y_{s+1}^n - \mathrm{B}_s^{n\cdot}\vec{U}_s - \epsilon_s^n\right)\right)^2\\ &= \lim_{N\to\infty}\frac{1}{N}\sum_{n=1}^{N}\sum_{0\le s,r<t}\alpha_s\alpha_r\left(Y_{s+1}^n - \mathrm{B}_s^{n\cdot}\vec{U}_s - \epsilon_s^n\right)\left(Y_{r+1}^n - \mathrm{B}_r^{n\cdot}\vec{U}_r - \epsilon_r^n\right)\\ &= \sum_{0\le s,r<t}\alpha_s\alpha_r\left(\lim_{N\to\infty}\frac{1}{N}\sum_{n=1}^{N}\left(Y_{s+1}^n - \mathrm{B}_s^{n\cdot}\vec{U}_s - \epsilon_s^n\right)\left(Y_{r+1}^n - \mathrm{B}_r^{n\cdot}\vec{U}_r - \epsilon_r^n\right)\right)\\ &\overset{\text{a.s.}}{=} \lim_{N\to\infty}\frac{\mu^2}{N^2}\left(\sum_{n=1}^{N}\sum_{0\le s<t}\alpha_sU_s^n\right)\left(\sum_{n=1}^{N}\sum_{0\le r<t}\alpha_rU_r^n\right) + \lim_{N\to\infty}\frac{\sigma^2}{N}\sum_{n=1}^{N}\sum_{0\le s,r<t}\alpha_s\alpha_rU_s^nU_r^n\\ &= \lim_{N\to\infty}\left(\frac{\mu}{N}\sum_{n=1}^{N}U_t^{\parallel n}\right)^2 + \lim_{N\to\infty}\frac{\sigma^2}{N}\sum_{n=1}^{N}\left(U_t^{\parallel n}\right)^2,\end{aligned} \tag{67}$$

where in the last line we used Eq. (35). Further, because the elements of $\vec{\epsilon}_t^{\,n}$ are independent zero mean Gaussian random variables, a straightforward application of Theorem 8 implies that for any term δ in this proof, we have:

$$\lim_{N\to\infty}\frac{1}{N}\sum_{n=1}^{N}\delta\epsilon_t^n \overset{\text{a.s.}}{=} 0, \tag{68}$$

and

$$\lim_{N\to\infty}\frac{1}{N}\sum_{n=1}^{N}\left(\epsilon_t^n\right)^2 \overset{\text{a.s.}}{=} \sigma_e^2. \tag{69}$$

Now, we first obtain Eq. (38a) for $s=t$. Based on Eq. (44), we can write

$$\begin{aligned}\lim_{N\to\infty}\frac{1}{N}\sum_{n=1}^{N}Y_{t+1}^n &\overset{\text{a.s.}}{=} \lim_{N\to\infty}\frac{1}{N}\sum_{n=1}^{N}\left(\widetilde{\mathrm{A}}^{n\cdot}\vec{U}_t^{\perp} + [\boldsymbol{R}_t\vec{\alpha}_t]_n + \mathrm{B}_t^{n\cdot}\vec{U}_t + \epsilon_t^n\right)\\ &\overset{\text{a.s.}}{=} \lim_{N\to\infty}\frac{\mu+\mu_t}{N}\sum_{n=1}^{N}g_t\left(Y_t^n,\vec{W}^n,\vec{X}^n\right)\\ &\overset{\text{a.s.}}{=} (\mu+\mu_t)\mathbb{E}\left[g_t\left(\nu_t+\rho_tZ,\vec{W},\vec{X}\right)\right] = \nu_{t+1},\end{aligned} \tag{70}$$

where we used the induction hypothesis (a), Eq. (62), Eq. (63), Eq. (66), and Eq. (68). The derivation of Eq. (38b) is similar and is removed to avoid repetition.

We next obtain Eq. (38c) for $s = r = t$. By Eq. (44), Eq. (64), Eq. (65), Eq. (67), Eq. (68), and Eq. (69), we can write

$$
\begin{aligned}
\lim_{N\to\infty} \frac{1}{N}\sum_{n=1}^{N}\left(Y_{t+1}^{n}\right)^2 &\overset{\text{a.s.}}{=} \lim_{N\to\infty} \frac{1}{N}\sum_{n=1}^{N}\left(\widetilde{\mathrm{A}}^{n\cdot}\vec{U}_t^{\perp} + [\boldsymbol{R}_t\vec{\alpha}_t]_n + \mathrm{B}_t^{n\cdot}\vec{U}_t + \epsilon_t^n\right)^2 \\
&\overset{\text{a.s.}}{=} \lim_{N\to\infty}\left(\frac{\mu}{N}\sum_{n=1}^{N} U_t^{\perp n}\right)^2 + \lim_{N\to\infty}\frac{\sigma^2}{N}\sum_{n=1}^{N}\left(U_t^{\perp n}\right)^2, \\
&\quad + \lim_{N\to\infty}\left(\frac{\mu}{N}\sum_{n=1}^{N} U_t^{\parallel n}\right)^2 + \lim_{N\to\infty}\frac{\sigma^2}{N}\sum_{n=1}^{N}\left(U_t^{\parallel n}\right)^2 \\
&\quad + \lim_{N\to\infty}\left(\frac{\mu_t}{N}\sum_{n=1}^{N} U_t^{n}\right)^2 + \lim_{N\to\infty}\frac{\sigma_t^2}{N}\sum_{n=1}^{N}\left(U_t^{n}\right)^2 + \sigma_e^2 \\
&\quad + \lim_{N\to\infty}\frac{2}{N}\sum_{n=1}^{N}\left(\widetilde{\mathrm{A}}^{n\cdot}\vec{U}_t^{\perp}\,[\boldsymbol{R}_t\vec{\alpha}_t]_n\right) + \lim_{N\to\infty}\frac{2}{N}\sum_{n=1}^{N}\left(\widetilde{\mathrm{A}}^{n\cdot}\vec{U}_t^{\perp}\mathrm{B}_t^{n\cdot}\vec{U}_t\right) \\
&\quad + \lim_{N\to\infty}\frac{2}{N}\sum_{n=1}^{N}\left([\boldsymbol{R}_t\vec{\alpha}_t]_n\,\mathrm{B}_t^{n\cdot}\vec{U}_t\right).
\end{aligned}
\tag{71}
$$

Note that the only random objects in the right-hand side of Eq. (71) are $\widetilde{\mathrm{A}}^{n\cdot}$ and $\mathrm{B}_t^{n\cdot}$. Thus, by Eq. (34), an argument similar to Eq. (61), and applying Theorem 8, we can write

$$
\begin{aligned}
\lim_{N\to\infty}\frac{2}{N}\sum_{n=1}^{N}\left(\widetilde{\mathrm{A}}^{n\cdot}\vec{U}_t^{\perp}\,[\boldsymbol{R}_t\vec{\alpha}_t]_n\right) &= \lim_{N\to\infty}\frac{2}{N}\sum_{n=1}^{N}\sum_{s=0}^{t-1}\alpha_s\widetilde{\mathrm{A}}^{n\cdot}\vec{U}_t^{\perp}\left(Y_{s+1}^n - \mathrm{B}_s^{n\cdot}\vec{U}_s - \epsilon_s^n\right) \\
&\overset{\text{a.s.}}{=} \lim_{N\to\infty}\frac{2}{N}\sum_{n=1}^{N}\frac{\mu}{N}\sum_{m=1}^{N}U_t^{\perp m}\left(\sum_{s=0}^{t-1}\alpha_s\left(Y_{s+1}^n - \mathrm{B}_s^{n\cdot}\vec{U}_s - \epsilon_s^n\right)\right) \\
&\overset{\text{a.s.}}{=} \lim_{N\to\infty}\frac{2\mu}{N}\left(\sum_{n=1}^{N}U_t^{\perp n}\right)\lim_{N\to\infty}\frac{\mu}{N}\left(\sum_{n=1}^{N}U_t^{\parallel n}\right),
\end{aligned}
\tag{72}
$$

where in the last line we used the induction hypothesis Eq. (38b) and Eq. (35). Likewise, we get

$$
\lim_{N\to\infty}\frac{2}{N}\sum_{n=1}^{N}\left(\mathrm{B}_t^{n\cdot}\vec{U}_t\,[\boldsymbol{R}_t\vec{\alpha}_t]_n\right) \overset{\text{a.s.}}{=} \lim_{N\to\infty}\frac{2\mu_t}{N}\left(\sum_{n=1}^{N}U_t^{n}\right)\lim_{N\to\infty}\frac{\mu}{N}\left(\sum_{n=1}^{N}U_t^{\parallel n}\right),
\tag{73}
$$

We continue the proof by calculating the next term using Eq. (61) and Theorem 8:

$$
\lim_{N\to\infty}\frac{2}{N}\sum_{n=1}^{N}\left(\widetilde{\mathrm{A}}^{n\cdot}\vec{U}_t^{\perp}\mathrm{B}_t^{n\cdot}\vec{U}_t\right) \overset{\text{a.s.}}{=} \lim_{N\to\infty}\frac{2\mu\mu_t}{N^2}\sum_{n=1}^{N}U_t^{\perp n}\sum_{n=1}^{N}U_t^{n}.
\tag{74}
$$

Combining Eq. (71)-Eq. (74) yields:

$$
\lim_{N\to\infty}\frac{1}{N}\sum_{n=1}^{N}\left(Y_{t+1}^{n}\right)^2 \overset{\text{a.s.}}{=} \lim_{N\to\infty}\left(\frac{\mu+\mu_t}{N}\sum_{n=1}^{N}U_t^{n}\right)^2 + \lim_{N\to\infty}\frac{\sigma^2+\sigma_t^2}{N}\sum_{n=1}^{N}\left(U_t^{n}\right)^2 + \sigma_e^2.
$$

The desired result is immediate by applying the induction hypothesis (a). We next derive Eq. (38d) for $r = t$ and $0 \le s \le t-1$. Considering Eq. (44) and Eq. (68), we can write

$$
\begin{aligned}
&\lim_{N\to\infty}\frac{1}{N}\sum_{n=1}^{N}Y_{s+1}^{n}Y_{t+1}^{n} \\
&\overset{\text{a.s.}}{=} \lim_{N\to\infty}\frac{1}{N}\sum_{n=1}^{N}Y_{s+1}^{n}\left(\widetilde{\mathrm{A}}^{n\cdot}\vec{U}_t^{\perp} + [\boldsymbol{R}_t\vec{\alpha}_t]_n + \mathrm{B}_t^{n\cdot}\vec{U}_t + \epsilon_t^n\right) \\
&\overset{\text{a.s.}}{=} \lim_{N\to\infty}\frac{1}{N}\sum_{n=1}^{N}\left(\left(\widetilde{\mathrm{A}}^{n\cdot}\vec{U}_t^{\perp}\right)Y_{s+1}^{n} + \left([\boldsymbol{R}_t\vec{\alpha}_t]_n\right)Y_{s+1}^{n} + \left(\mathrm{B}_t^{n\cdot}\vec{U}_t\right)Y_{s+1}^{n}\right).
\end{aligned}
\tag{75}
$$

Note that after conditioning on $\mathcal{G}_t$, the quantity Y^n_{s+1} is known. Then, applying Theorem 8 and considering Eq. (61), we obtain

$$\begin{aligned}
\lim_{N\to\infty}\frac{1}{N}\sum_{n=1}^{N}\left(\widetilde{\mathrm{A}}^{n\cdot}\vec{U}_t^{\perp}\right)Y^n_{s+1} &\overset{\text{a.s.}}{=} \lim_{N\to\infty}\frac{\mu}{N^2}\sum_{n=1}^{N}\left(Y^n_{s+1}\sum_{m=1}^{N}U_t^{\perp m}\right)\\
&= \lim_{N\to\infty}\frac{\mu}{N^2}\left(\sum_{n=1}^{N}Y^n_{s+1}\sum_{n=1}^{N}U_t^{\perp n}\right)\\
&\overset{\text{a.s.}}{=} \lim_{N\to\infty}\frac{\mu(\mu+\mu_s)}{N^2}\left(\sum_{n=1}^{N}U^n_s\sum_{n=1}^{N}U_t^{\perp n}\right).
\end{aligned} \tag{76}$$

In a similar fashion, we can see that

$$\begin{aligned}
\lim_{N\to\infty}\frac{1}{N}\sum_{n=1}^{N}\left(\mathrm{B}^{n\cdot}_t\vec{U}_t\right)Y^n_{s+1} &\overset{\text{a.s.}}{=} \lim_{N\to\infty}\frac{\mu_t}{N^2}\sum_{n=1}^{N}\left(Y^n_{s+1}\sum_{m=1}^{N}U_t^{m}\right)\\
&= \lim_{N\to\infty}\frac{\mu_t}{N^2}\left(\sum_{n=1}^{N}Y^n_{s+1}\sum_{n=1}^{N}U_t^{n}\right)\\
&\overset{\text{a.s.}}{=} \lim_{N\to\infty}\frac{\mu_t(\mu+\mu_s)}{N^2}\left(\sum_{n=1}^{N}U^n_s\sum_{n=1}^{N}U_t^{n}\right).
\end{aligned} \tag{77}$$

Lastly, considering Eq. (34), by the induction hypothesis Eq. (38e), we get

$$\begin{aligned}
\lim_{N\to\infty}\frac{1}{N}\sum_{n=1}^{N}\left([\boldsymbol{R}_t\vec{\alpha}_t]_n\right)Y^n_{s+1} &= \lim_{N\to\infty}\frac{1}{N}\sum_{n=1}^{N}\left(\sum_{r=0}^{t-1}\alpha_r\left(Y^n_{r+1}-\mathrm{B}^{n\cdot}_r\vec{U}_r-\epsilon^n_r\right)Y^n_{s+1}\right)\\
&\overset{\text{a.s.}}{=} \sum_{r=0}^{t-1}\alpha_r\left(\lim_{N\to\infty}\frac{\mu(\mu+\mu_s)}{N^2}\sum_{n=1}^{N}U^n_r\sum_{n=1}^{N}U^n_s+\lim_{N\to\infty}\frac{\sigma^2}{N}\sum_{n=1}^{N}U^n_rU^n_s\right)\\
&\overset{\text{a.s.}}{=} \lim_{N\to\infty}\left(\frac{\mu(\mu+\mu_s)}{N^2}\sum_{n=1}^{N}\sum_{r=0}^{t-1}\alpha_rU^n_r\sum_{n=1}^{N}U^n_s+\frac{\sigma^2}{N}\sum_{n=1}^{N}\sum_{r=0}^{t-1}\alpha_rU^n_rU^n_s\right)\\
&= \lim_{N\to\infty}\frac{\mu(\mu+\mu_s)}{N^2}\left(\sum_{n=1}^{N}U^n_s\sum_{n=1}^{N}U_t^{\parallel n}\right)+\lim_{N\to\infty}\frac{\sigma^2}{N}\sum_{n=1}^{N}\left(U_t^{\parallel n}U^n_s\right)\\
&= \lim_{N\to\infty}\frac{\mu(\mu+\mu_s)}{N^2}\left(\sum_{n=1}^{N}U^n_s\sum_{n=1}^{N}U_t^{\parallel n}\right)+\lim_{N\to\infty}\frac{\sigma^2}{N}\sum_{n=1}^{N}\left(U^n_tU^n_s\right).
\end{aligned} \tag{78}$$

In the last line of Eq. (78), we used the fact that $\left\langle\vec{U}_t,\vec{U}_s\right\rangle=\left\langle\vec{U}_t^{\parallel},\vec{U}_s\right\rangle$ as $\vec{U}_t^{\perp}\perp\vec{U}_s$. Considering Eq. (75)-Eq. (78) together concludes the proof of Eq. (38d).

We follow a similar approach as above to obtain Eq. (38e). Fixing $0\le r\le t-1$ and letting $s=t$, by Eq. (44), Eq. (76), and Eq. (78), we can write

$$\begin{aligned}
&\lim_{N\to\infty}\frac{1}{N}\sum_{n=1}^{N}\left(Y^n_{t+1}-\mathrm{B}^{n\cdot}_t\vec{U}_t-\epsilon^n_t\right)Y^n_{r+1}\\
&\overset{\text{a.s.}}{=} \lim_{N\to\infty}\frac{1}{N}\sum_{n=1}^{N}\left(\widetilde{\mathrm{A}}^{n\cdot}\vec{U}_t^{\perp}+[\boldsymbol{R}_t\vec{\alpha}_t]_n\right)Y^n_{r+1}\\
&\overset{\text{a.s.}}{=} \lim_{N\to\infty}\frac{\mu(\mu+\mu_r)}{N^2}\left(\sum_{n=1}^{N}U^n_r\sum_{n=1}^{N}U_t^{\perp n}\right)\\
&\quad+\lim_{N\to\infty}\frac{\mu(\mu+\mu_r)}{N^2}\left(\sum_{n=1}^{N}U^n_r\sum_{n=1}^{N}U_t^{\parallel n}\right)+\lim_{N\to\infty}\frac{\sigma^2}{N}\sum_{n=1}^{N}\left(U^n_tU^n_r\right)
\end{aligned}$$

Likewise, we can show the result for the case that $r = t$ and $0 \le s \le t-1$:

$$\begin{aligned}
&\lim_{N\to\infty}\frac{1}{N}\sum_{n=1}^{N}\left(Y^n_{s+1}-\mathrm{B}^{n\cdot}_s\vec{U}_s-\epsilon^n_s\right)Y^n_{t+1}\\
&\overset{\text{a.s.}}{=}\lim_{N\to\infty}\frac{1}{N}\sum_{n=1}^{N}\left(Y^n_{s+1}-\mathrm{B}^{n\cdot}_s\vec{U}_s-\epsilon^n_s\right)\left(\widetilde{\mathrm{A}}^{n\cdot}\vec{U}^{\perp}_t+[\boldsymbol{R}_t\vec{\alpha}_t]_n+\mathrm{B}^{n\cdot}_t\vec{U}_t+\epsilon^n_t\right)\\
&=\lim_{N\to\infty}\frac{1}{N}\sum_{n=1}^{N}\left(\widetilde{\mathrm{A}}^{n\cdot}\vec{U}^{\perp}_t\right)\left(Y^n_{s+1}-\mathrm{B}^{n\cdot}_s\vec{U}_s-\epsilon^n_s\right)\\
&\quad+\lim_{N\to\infty}\frac{1}{N}\sum_{n=1}^{N}\left([\boldsymbol{R}_t\vec{\alpha}_t]_n\right)\left(Y^n_{s+1}-\mathrm{B}^{n\cdot}_s\vec{U}_s-\epsilon^n_s\right)\\
&\quad+\lim_{N\to\infty}\frac{1}{N}\sum_{n=1}^{N}\left(\mathrm{B}^{n\cdot}_t\vec{U}_t\right)\left(Y^n_{s+1}-\mathrm{B}^{n\cdot}_s\vec{U}_s-\epsilon^n_s\right),
\end{aligned}\tag{79}$$

where we also used Eq. (68). By Theorem 8 and the induction hypothesis Eq. (38b), we have

$$\lim_{N\to\infty}\frac{1}{N}\sum_{n=1}^{N}\left(\widetilde{\mathrm{A}}^{n\cdot}\vec{U}^{\perp}_t\right)\left(Y^n_{s+1}-\mathrm{B}^{n\cdot}_s\vec{U}_s-\epsilon^n_s\right)\overset{\text{a.s.}}{=}\lim_{N\to\infty}\frac{\mu^2}{N^2}\sum_{n=1}^{N}U^{\perp n}_t\sum_{n=1}^{N}U^n_s,\tag{80}$$

as well as,

$$\lim_{N\to\infty}\frac{1}{N}\sum_{n=1}^{N}\left(\mathrm{B}^{n\cdot}_t\vec{U}_t\right)\left(Y^n_{s+1}-\mathrm{B}^{n\cdot}_s\vec{U}_s-\epsilon^n_s\right)\overset{\text{a.s.}}{=}\lim_{N\to\infty}\frac{\mu\mu_t}{N^2}\sum_{n=1}^{N}U^n_t\sum_{n=1}^{N}U^n_s.\tag{81}$$

Finally, Eq. (34), Eq. (35), and the induction hypothesis Eq. (38f) imply that

$$\begin{aligned}
&\lim_{N\to\infty}\frac{1}{N}\sum_{n=1}^{N}\left([\boldsymbol{R}_t\vec{\alpha}_t]_n\right)\left(Y^n_{s+1}-\mathrm{B}^{n\cdot}_s\vec{U}_s-\epsilon^n_s\right)\\
&=\lim_{N\to\infty}\frac{1}{N}\sum_{n=1}^{N}\sum_{r=0}^{t-1}\alpha_r\left(Y^n_{r+1}-\mathrm{B}^{n\cdot}_r\vec{U}_r-\epsilon^n_r\right)\left(Y^n_{s+1}-\mathrm{B}^{n\cdot}_s\vec{U}_s-\epsilon^n_s\right)\\
&\overset{\text{a.s.}}{=}\lim_{N\to\infty}\frac{\mu^2}{N^2}\left(\sum_{n=1}^{N}U^n_s\right)\left(\sum_{n=1}^{N}U^{\parallel n}_t\right)+\lim_{N\to\infty}\frac{\sigma^2}{N}\sum_{n=1}^{N}U^n_sU^n_t,
\end{aligned}\tag{82}$$

where in the last line, we used $\left\langle\vec{U}_t,\vec{U}_s\right\rangle=\left\langle\vec{U}^{\parallel}_t,\vec{U}_s\right\rangle$. The desired result follows by aggregating Eq. (79)-Eq. (82).

To conclude the proof of part (b), we need to show Eq. (38f) for $r = s = t$ as well as $s = t,\ 0 \le r < t-1$. If $r = s = t$, then considering Eq. (44), the result immediately follows from Eq. (64), Eq. (67), and Eq. (72). For the case that $s = t,\ 0 \le r < t-1$, by Eq. (44), we have

$$\begin{aligned}
&\lim_{N\to\infty}\frac{1}{N}\sum_{n=1}^{N}\left(Y^n_{t+1}-\mathrm{B}^{n\cdot}_t\vec{U}_t-\epsilon^n_t\right)\left(Y^n_{r+1}-\mathrm{B}^{n\cdot}_r\vec{U}_r-\epsilon^n_r\right)\\
&=\lim_{N\to\infty}\frac{1}{N}\sum_{n=1}^{N}\left(\widetilde{\mathrm{A}}^{n\cdot}U^{\perp}_t+[\boldsymbol{R}_t\vec{\alpha}_t]_n\right)\left(Y^n_{r+1}-\mathrm{B}^{n\cdot}_r\vec{U}_r-\epsilon^n_r\right)\\
&\overset{\text{a.s.}}{=}\lim_{N\to\infty}\frac{\mu^2}{N^2}\sum_{n=1}^{N}U^{\perp n}_t\sum_{n=1}^{N}U^n_r+\lim_{N\to\infty}\frac{\mu^2}{N^2}\left(\sum_{n=1}^{N}U^n_r\right)\left(\sum_{n=1}^{N}U^{\parallel n}_t\right)+\lim_{N\to\infty}\frac{\sigma^2}{N}\sum_{n=1}^{N}U^n_rU^n_t\\
&=\lim_{N\to\infty}\frac{\mu^2}{N^2}\left(\sum_{n=1}^{N}U^n_r\right)\left(\sum_{n=1}^{N}U^n_t\right)+\lim_{N\to\infty}\frac{\sigma^2}{N}\sum_{n=1}^{N}U^n_rU^n_t,
\end{aligned}\tag{83}$$

where we used Eq. (80) and Eq. (82). The induction hypothesis (a) implies the last equality in Eq. (38f).

(a) Given, $\vec{Y}_1(N),\ldots,\vec{Y}_t(N)$, $\boldsymbol{W}(N)$, and $\boldsymbol{X}(N)$, define

$$\Psi_n(N):=\psi\left(Y^n_1(N),\ldots,Y^n_t(N),Y^n_{t+1}(N),\vec{W}^n(N),\vec{X}^n(N)\right).$$

Dropping the notation N in the right-hand side, based on Eq. (44), we can write

$$\Psi_n(N)\Big|_{\mathcal{G}_t} \overset{\mathrm{d}}{=} \psi\left(Y_1^n, \ldots, Y_t^n, \left[\widetilde{\mathbf{A}}\vec{U}_t^{\perp} + \boldsymbol{R}_t\vec{\alpha}_t + \mathbf{B}_t\vec{U}_t + \vec{\epsilon}_t\right]_n, \vec{W}^n, \vec{X}^n\right),$$

where $\widetilde{\mathbf{A}}$ has the same distribution as $\mathbf{A}$ independent of everything else. We also let

$$\widetilde{\Psi}_n(N) = \Psi_n(N) - \mathbb{E}_{\mathbf{G}_t,\vec{\epsilon}_t}[\Psi_n(N)].$$

where $\mathbb{E}_{\mathbf{G}_t,\vec{\epsilon}_t}$ denotes the expectation with respect to the randomness of the matrices $\widetilde{\mathbf{A}}$, $\mathbf{B}_t$, as well as the noise vector $\vec{\epsilon}_t$. We follow the same approach as Step 1-(a). Note that given $\mathcal{G}_t$, the elements of $\widetilde{\mathbf{A}}\vec{U}_t^{\perp} + \mathbf{B}_t\vec{U}_t + \vec{\epsilon}_t$ are i.i.d. Gaussian random variables with mean $\tilde{\nu}_{tN}$ and variance $\tilde{\rho}_{tN}^2$:

$$\begin{aligned} \tilde{\nu}_{tN} &:= \mathbb{E}\left[[\widetilde{\mathbf{A}}\vec{U}_t^{\perp} + \mathbf{B}_t\vec{U}_t + \vec{\epsilon}_t]_n \Big| \vec{U}_t\right] = \frac{\mu}{N}\sum_{n=1}^{N} U_t^{\perp n} + \frac{\mu_t}{N}\sum_{n=1}^{N} U_t^n, \\ \tilde{\rho}_{tN}^2 &:= \mathrm{Var}\left[[\widetilde{\mathbf{A}}\vec{U}_t^{\perp} + \mathbf{B}_t\vec{U}_t + \vec{\epsilon}_t]_n \Big| \vec{U}_t\right] = \frac{\sigma^2}{N}\sum_{n=1}^{N} \left(U_t^{\perp n}\right)^2 + \frac{\sigma_t^2}{N}\sum_{n=1}^{N} (U_t^n)^2 + \sigma_e^2, \end{aligned} \quad [84]$$

where $U_t^n = g_t\left(Y_t^n, \vec{W}^n, \vec{X}^n\right)$ is the nth element of the column vector $\vec{U}_t$ and let

$$\tilde{\nu}_t = \lim_{N\to\infty} \tilde{\nu}_{tN}, \qquad \tilde{\rho}_t^2 = \lim_{N\to\infty} \tilde{\rho}_{tN}^2. \quad [85]$$

We show that $\tilde{\nu}_t$ and $\tilde{\rho}_t^2$ are almost surely finite. That is, with a probability of 1, we have

$$\lim_{N\to\infty} \frac{1}{N}\sum_{n=1}^{N} U_t^{\perp n} < \infty, \quad \lim_{N\to\infty} \frac{1}{N}\sum_{n=1}^{N} U_t^n < \infty, \quad \lim_{N\to\infty} \frac{1}{N}\sum_{n=1}^{N} \left(U_t^{\perp n}\right)^2 < \infty, \quad \lim_{N\to\infty} \frac{1}{N}\sum_{n=1}^{N} (U_t^n)^2 < \infty. \quad [86]$$

By definition, we can write

$$\begin{aligned} \frac{1}{N}\sum_{n=1}^{N} U_t^{\perp n} &= \frac{1}{N}\sum_{n=1}^{N} U_t^n - \frac{1}{N}\sum_{n=1}^{N} U_t^{\parallel n}, \\ \frac{1}{N}\sum_{n=1}^{N} \left(U_t^{\perp n}\right)^2 = \left\langle \vec{U}_t^{\perp}, \vec{U}_t^{\perp} \right\rangle &= \left\langle \vec{U}_t, \vec{U}_t \right\rangle - \left\langle \vec{U}_t^{\parallel}, \vec{U}_t^{\parallel} \right\rangle = \frac{1}{N}\sum_{n=1}^{N} (U_t^n)^2 - \frac{1}{N}\sum_{n=1}^{N} \left(U_t^{\parallel n}\right)^2. \end{aligned} \quad [87]$$

Then, by the induction hypothesis and Assumption 3-(i) for functions $\psi = g_t\left(Y_t^n, \vec{W}^n, \vec{X}^n\right)$ and $\psi = g_t\left(Y_t^n, \vec{W}^n, \vec{X}^n\right)^2$, we get

$$\begin{aligned} \lim_{N\to\infty} \frac{1}{N}\sum_{n=1}^{N} U_t^n &= \lim_{N\to\infty} \frac{1}{N}\sum_{n=1}^{N} g_t\left(Y_t^n, \vec{W}^n, \vec{X}^n\right) \overset{\text{a.s.}}{=} \mathbb{E}\left[g_t\left(\nu_t + \rho_t Z, \vec{W}, \vec{X}\right)\right] < \infty \\ \lim_{N\to\infty} \frac{1}{N}\sum_{n=1}^{N} (U_t^n)^2 &= \lim_{N\to\infty} \frac{1}{N}\sum_{n=1}^{N} g_t\left(Y_t^n, \vec{W}^n, \vec{X}^n\right)^2 \overset{\text{a.s.}}{=} \mathbb{E}\left[g_t\left(\nu_t + \rho_t Z, \vec{W}, \vec{X}\right)^2\right] < \infty, \end{aligned} \quad [88]$$

where $Z \sim \mathcal{N}(0, 1)$. Further, by Eq. (35), we have

$$\begin{aligned} \frac{1}{N}\sum_{n=1}^{N} U_t^{\parallel n} &= \frac{1}{N}\sum_{n=1}^{N}\sum_{s=0}^{t-1} \alpha_s U_s^n = \sum_{s=0}^{t-1} \frac{\alpha_s}{N}\sum_{n=1}^{N} U_s^n \\ \frac{1}{N}\sum_{n=1}^{N} \left(U_t^{\parallel n}\right)^2 &= \frac{1}{N}\sum_{n=1}^{N} \left(\sum_{s=0}^{t-1} \alpha_s U_s^n\right)^2 = \sum_{r,s=0}^{t-1} \alpha_r\alpha_s \left\langle \vec{U}_r, \vec{U}_s \right\rangle. \end{aligned}$$

Considering Corollary 3, the vector $\vec{\alpha}$ has a finite limit as $N \to \infty$. Similar to Eq. (88), the induction hypothesis for functions $\psi = g_s\left(Y_s^n, \vec{W}^n, \vec{X}^n\right)$ and $\psi = g_r\left(Y_r^n, \vec{W}^n, \vec{X}^n\right) g_s\left(Y_s^n, \vec{W}^n, \vec{X}^n\right)$ implies that almost surely

$$\lim_{N\to\infty} \frac{1}{N}\sum_{n=1}^{N} U_t^{\parallel n} < \infty, \qquad \lim_{N\to\infty} \frac{1}{N}\sum_{n=1}^{N} \left(U_t^{\parallel n}\right)^2 = \lim_{N\to\infty} \sum_{r,s=0}^{t-1} \alpha_r\alpha_s \left\langle \vec{U}_r, \vec{U}_s \right\rangle < \infty. \quad [89]$$

Consequently, by Eq. (87)-Eq. (89), we get the result in Eq. (86). This also implies that $\tilde{\nu}_{tN}$ and $\tilde{\rho}^2_{tN}$, in Eq. (84), are almost surely bounded for all values of N. As an immediate result, for $l \geq 1$, we get

$$\mathbb{E}\left[\left|\left[\widetilde{\mathbf{A}}\vec{U}_t^{\perp} + \mathbf{B}_t\vec{U}_t + \vec{\epsilon}_t\right]_n + \left[\boldsymbol{R}_t\vec{\alpha}_t\right]_n\right|^l\right] \leq 2^{l-1}\mathbb{E}\left[\left|\left[\widetilde{\mathbf{A}}\vec{U}_t^{\perp} + \mathbf{B}_t\vec{U}_t + \vec{\epsilon}_t\right]_n\right|^l + \left|\left[\boldsymbol{R}_t\vec{\alpha}_t\right]_n\right|^l\right] \leq c, \tag{90}$$

where c is a constant independent of N and we used the inequality $(v_1 + v_2)^l \leq 2^{l-1}(v_1^l + v_2^l)$, $v_1, v_2 \geq 0$. Note that in Eq. (90), given $\mathcal{G}_t$, the term $\boldsymbol{R}_t\vec{\alpha}_t$ is deterministic and bounded in view of Corollary 3. Now, fixing $0 < \kappa < 1$ and using the fact that $\psi \in \mathcal{CP}(k)$ and so $|\psi(\vec{\omega})| \leq c(1 + \|\vec{\omega}\|^k)$, similar to Eq. (50), we get that

$$\frac{1}{N}\sum_{n=1}^{N}\mathbb{E}\left[\left|\widetilde{\Psi}_n(N)\right|^{2+\kappa}\right] \leq cN^{\kappa/2}. \tag{91}$$

In order to obtain Eq. (91), the only difference, compared to Eq. (50), is that we need the following inequality for $l \geq 1$ as a straightforward application of Jensen's inequality:

$$\left(\frac{v_1 + \ldots + v_j}{l}\right)^l \leq \frac{v_1^l + \ldots + v_j^l}{l}, \qquad v_i \geq 0.$$

Therefore, we can apply the SLLN for triangular arrays, Theorem 8. We get

$$\begin{aligned} &\lim_{N\to\infty}\frac{1}{N}\sum_{n=1}^{N}\psi\big(Y_1^n, \ldots, Y_t^n, Y_{t+1}^n, \vec{W}^n, \vec{X}^n\big) \\ &\overset{\text{a.s.}}{=} \lim_{N\to\infty}\frac{1}{N}\sum_{n=1}^{N}\mathbb{E}_{\mathbf{G}_t,\vec{\epsilon}_t}\left[\psi\left(Y_1^n, \ldots, Y_t^n, \left[\widetilde{\mathbf{A}}\vec{U}_t^{\perp} + \boldsymbol{R}_t\vec{\alpha}_t + \mathbf{B}_t\vec{U}_t + \vec{\epsilon}_t\right]_n, \vec{W}^n, \vec{X}^n\right)\right]. \end{aligned} \tag{92}$$

Note that for any Borel measurable function ϕ and $Z \sim \mathcal{N}(0,1)$, the following random variables have the same distribution:

$$\phi\left(\left[\widetilde{\mathbf{A}}\vec{U}_t^{\perp} + \boldsymbol{R}_t\vec{\alpha}_t + \mathbf{B}_t\vec{U}_t + \vec{\epsilon}_t\right]_n\right), \qquad \phi\left(\tilde{\nu}_{tN} + \tilde{\rho}_{tN}Z + \sum_{s=0}^{t-1}\alpha_s\big(Y_{s+1}^n - \mathrm{B}_s^{n\cdot}\vec{U}_s - \epsilon_s^n\big)\right).$$

Thus, we define

$$\begin{aligned} &\widehat{\psi}\big(Y_1^n, Y_1^n - \mathrm{B}_0^{n\cdot}\vec{U}_0 - \epsilon_0^n, \ldots, Y_t^n, Y_t^n - \mathrm{B}_{t-1}^{n\cdot}\vec{U}_{t-1} - \epsilon_{t-1}^n, \vec{W}^n, \vec{X}^n\big) \\ &:= \mathbb{E}_Z\left[\psi\left(Y_1^n, \ldots, Y_t^n, \tilde{\nu}_{tN} + \tilde{\rho}_{tN}Z + \sum_{s=0}^{t-1}\alpha_s\big(Y_{s+1}^n - \mathrm{B}_s^{n\cdot}\vec{U}_s - \epsilon_s^n\big), \vec{W}^n, \vec{X}^n\right)\right]. \end{aligned}$$

Using the induction hypothesis in Eq. (56), for the function $\widehat{\psi}$, by Eq. (92), we have

$$\begin{aligned} &\lim_{N\to\infty}\frac{1}{N}\sum_{n=1}^{N}\psi\big(Y_1^n, \ldots, Y_t^n, Y_{t+1}^n, \vec{W}^n, \vec{X}^n\big) \\ &\overset{\text{a.s.}}{=} \lim_{N\to\infty}\frac{1}{N}\sum_{n=1}^{N}\widehat{\psi}\big(Y_1^n, Y_1^n - \mathrm{B}_0^{n\cdot}\vec{U}_0 - \epsilon_0^n, \ldots, Y_t^n, Y_t^n - \mathrm{B}_{t-1}^{n\cdot}\vec{U}_{t-1} - \epsilon_{t-1}^n, \vec{W}^n, \vec{X}^n\big) \\ &\overset{\text{a.s.}}{=} \mathbb{E}\left[\widehat{\psi}\big(\nu_1 + \rho_1 Z_1, \bar{\nu}_1 + \bar{\rho}_1 Z_1', \ldots, \nu_t + \rho_t Z_t, \bar{\nu}_t + \bar{\rho}_t Z_t', \vec{W}, \vec{X}\big)\right] \\ &= \mathbb{E}\mathbb{E}_Z\left[\psi\left(\nu_1 + \rho_1 Z_1, \ldots, \nu_t + \rho_t Z_t, \tilde{\nu}_t + \tilde{\rho}_t Z + \sum_{s=0}^{t-1}\alpha_s\big(\bar{\nu}_{s+1} + \bar{\rho}_{s+1}Z_{s+1}'\big), \vec{W}, \vec{X}\right)\right], \end{aligned} \tag{93}$$

where Z is an independent Normal random variable. Similar to Eq. (49), we used the DCT and the continuous mapping theorem to interchange the limit and the expectation and then pass the limit through the function in Eq. (93). Now, we need to show that

$$\begin{aligned} \mathbb{E}\left[\tilde{\nu}_t + \tilde{\rho}_t Z + \sum_{s=0}^{t-1}\alpha_s\big(\bar{\nu}_{s+1} + \bar{\rho}_{s+1}Z_{s+1}'\big)\right] &= \nu_{t+1}, \\ \mathrm{Var}\left[\tilde{\nu}_t + \tilde{\rho}_t Z + \sum_{s=0}^{t-1}\alpha_s\big(\bar{\nu}_{s+1} + \bar{\rho}_{s+1}Z_{s+1}'\big)\right] &= \rho_{t+1}^2. \end{aligned} \tag{94}$$

Then, the proof is complete because $(Z'_1, \ldots, Z'_t)$ has a joint Normal distribution independent of Z and so the random variable $\tilde{\nu}_t + \tilde{\rho}_t Z + \sum_{s=0}^{t-1} \alpha_s \big(\bar{\nu}_{s+1} + \bar{\rho}_{s+1} Z'_{s+1}\big)$ is Gaussian as well. To obtain Eq. (94), we let $\psi(y_1, \ldots, y_{t+1}, w, \vec{X}) = y_{t+1}$ and $\psi(y_1, \ldots, y_{t+1}, w, \vec{X}) = y_{t+1}^2$ in Eq. (93). We get

$$\begin{aligned} \lim_{N\to\infty} \frac{1}{N} \sum_{n=1}^{N} Y_{t+1}^n &= \mathbb{E}\left[\tilde{\nu}_t + \tilde{\rho}_t Z + \sum_{s=0}^{t-1} \alpha_s \big(\bar{\nu}_{s+1} + \bar{\rho}_{s+1} Z_{s+1}\big)\right], \\ \lim_{N\to\infty} \frac{1}{N} \sum_{n=1}^{N} \big(Y_{t+1}^n\big)^2 &= \mathbb{E}\left[\left(\tilde{\nu}_t + \tilde{\rho}_t Z + \sum_{s=0}^{t-1} \alpha_s \big(\bar{\nu}_{s+1} + \bar{\rho}_{s+1} Z_{s+1}\big)\right)^2\right]. \end{aligned} \tag{95}$$

But, by part (b), we have

$$\lim_{N\to\infty} \frac{1}{N} \sum_{n=1}^{N} Y_{t+1}^n \overset{\text{a.s.}}{=} \nu_{t+1}, \qquad \lim_{N\to\infty} \frac{1}{N} \sum_{n=1}^{N} {Y_{t+1}^n}^2 \overset{\text{a.s.}}{=} \nu_{t+1}^2 + \rho_{t+1}^2.$$

That yields the desired result in Eq. (94).

To conclude the proof, we need to show that the relations in Eq. (56) and Eq. (57) hold true for $s = t$. Following a similar argument as above, the former result is immediate. Here, we show that Eq. (57) is true for $s = t$; that is, for $\phi : \mathbb{R}^{2(t+1)+T+M} \mapsto \mathbb{R}$ within $\mathcal{CP}(\frac{k}{2})$, we claim that

$$\begin{aligned} \lim_{N\to\infty} \frac{1}{N} \sum_{n=1}^{N} \Big(& g_0\big(Y_0^n, \vec{W}^n, \vec{X}^n\big) \phi\big(Y_1^n, Y_1^n - \mathrm{B}_0^{n\cdot} \vec{U}_0 - \epsilon_0^n, \ldots, \\ & Y_{t+1}^n, Y_{t+1}^n - \mathrm{B}_t^{n\cdot} \vec{U}_t - \epsilon_t^n, \vec{W}^n, \vec{X}^n\big)\Big) \\ \overset{\text{a.s.}}{=} \mathbb{E}\Big[& \bar{g}_0(\vec{W}, \vec{X}) \phi\big(\nu_1 + \rho_1 Z_1, \bar{\nu}_1 + \bar{\rho}_1 Z'_1, \ldots, \nu_{t+1} + \rho_{t+1} Z_{t+1}, \bar{\nu}_{t+1} + \bar{\rho}_{t+1} Z'_{t+1}, \vec{W}, \vec{X}\big)\Big], \end{aligned} \tag{96}$$

Note that the vector $\vec{Y}_0$ is given and both g_0 and ϕ are $\mathcal{CP}(\frac{k}{2})$ functions. Therefore, we can verify the conditions of Theorem 8 by following the same argument as the one resulting in Eq. (91). Then, applying the SLLN for the triangular arrays, similar to Eq. (93), we get

$$\begin{aligned} \lim_{N\to\infty} \frac{1}{N} \sum_{n=1}^{N} \Big(& g_0\big(Y_0^n, \vec{W}^n, \vec{X}^n\big) \phi\big(Y_1^n, Y_1^n - \mathrm{B}_0^{n\cdot} \vec{U}_0 - \epsilon_0^n, \ldots, \\ & Y_{t+1}^n, Y_{t+1}^n - \mathrm{B}_t^{n\cdot} \vec{U}_t - \epsilon_t^n, \vec{W}^n, \vec{X}^n\big)\Big) \\ \overset{\text{a.s.}}{=} \lim_{N\to\infty} \frac{1}{N} \sum_{n=1}^{N} & \mathbb{E}_{\mathbf{G}_t, \vec{\epsilon}_t}\Big[g_0\big(Y_0^n, \vec{W}^n, \vec{X}^n\big) \phi\big(Y_1^n, Y_1^n - \mathrm{B}_0^{n\cdot} \vec{U}_0 - \epsilon_0^n, \ldots, \\ & Y_t^n, Y_t^n - \mathrm{B}_{t-1}^{n\cdot} \vec{U}_{t-1} - \epsilon_{t-1}^n, \\ & \Big[\widetilde{\mathbf{A}} \vec{U}_t^{\perp} + \boldsymbol{R}_t \vec{\alpha}_t + \mathrm{B}_t^{n\cdot} \vec{U}_t + \vec{\epsilon}_t\Big]_n, \Big[\widetilde{\mathbf{A}} \vec{U}_t^{\perp} + \boldsymbol{R}_t \vec{\alpha}_t\Big]_n, \vec{W}^n, \vec{X}^n\big)\Big] \\ \overset{\text{a.s.}}{=} \mathbb{E}\mathbb{E}_Z \Big[& \bar{g}_0(\vec{W}, \vec{X}) \phi\big(\nu_1 + \rho_1 Z_1, \bar{\nu}_1 + \bar{\rho}_1 Z'_1, \ldots, \nu_t + \rho_t Z_t, \bar{\nu}_t + \bar{\rho}_t Z'_t, \tilde{\nu}_t + \tilde{\rho}_t Z + \sum_{s=0}^{t-1} \alpha_s \big(\bar{\nu}_{s+1} + \bar{\rho}_{s+1} Z'_{s+1}\big), \\ & \bar{\nu}_t + \bar{\rho}_t Z' + \sum_{s=0}^{t-1} \alpha_s \big(\bar{\nu}_{s+1} + \bar{\rho}_{s+1} Z'_{s+1}\big), \vec{W}, \vec{X}\big)\Big], \end{aligned}$$

where in the last equality we used the induction hypothesis stated in Eq. (57). An argument similar to Eq. (94) besides Eq. (38b) and Eq. (38f) concludes the proof. □

11.5. Proof of Theorem 2: Strong consistency of the estimator. Without loss of generality, and by relabeling, we assume that Assumptions 3-(iv) and (v) hold true for $t = -1$. This allows us to consider the state evolution equations in Eq. (4) for $t \geq 0$. Now, let f be the function corresponding to Algorithm 2. That is,

$$f : \mathbb{R}^{T+1} \mapsto \mathbb{R}^{T+1}, \qquad \Big(\widehat{\mathrm{TTE}}_0(\breve{\pi}, 0), \ldots, \widehat{\mathrm{TTE}}_T(\breve{\pi}, 0)\Big) = f\big(\hat{\nu}_0, \ldots, \hat{\nu}_T\big) \tag{97}$$

Here, the function f maps the sample means of the observed outcomes over time to the output of Algorithm 2. Consequently, f implicitly depends on the other inputs of the algorithm, including the desired treatment level denoted by $\breve{\pi}$. Below, we first

demonstrate that f is a continuous function in its arguments. In the second step, we show that $\left(\mathrm{TTE}_0(\check{\pi},0),\ldots,\mathrm{TTE}_T(\check{\pi},0)\right) = f\left(\nu_0(\mathcal{E}),\ldots,\nu_T(\mathcal{E})\right)$. Then, considering Theorem 1, we know that $\nu_t(\mathcal{E}) \overset{\text{a.s.}}{=} \lim_{N\to\infty}\hat{\nu}_t$, $t\in[T]_0$, which implies the following and concludes the proof:

$$\begin{aligned}\lim_{N\to\infty}\left(\widehat{\mathrm{TTE}}_0(\check{\pi},0),\ldots,\widehat{\mathrm{TTE}}_T(\check{\pi},0)\right) &= \lim_{N\to\infty} f\left(\hat{\nu}_0,\ldots,\hat{\nu}_T\right)\\ &\overset{\text{a.s.}}{=} f\left(\nu_0(\mathcal{E}),\ldots,\nu_T(\mathcal{E})\right)\\ &= \left(\mathrm{TTE}_0(\check{\pi},0),\ldots,\mathrm{TTE}_T(\check{\pi},0)\right),\end{aligned}$$

where the second line holds due to the continuous mapping theorem, Theorem 2.3 in (36).

Step 1. In order to verify the continuity of f, note that regression coefficients a_1, b_1, a_2, and b_2 are continuous in the input data $\left(\hat{\nu}_0,\ldots,\hat{\nu}_T\right)$. As a result, the coefficients $(\widehat{\Xi},\widehat{\Gamma},\widehat{\Lambda})$ can also be understood as continuous functions of the input data. Now, we use an induction on t to show that $\check{\nu}_t$ and $\widehat{\mathrm{TTE}}_t(\check{\pi},0)$ are continuous in $\left((\hat{\nu}_0,\ldots,\hat{\nu}_T\right)$, as well. The result for $t=0$ is immediate by the definition. For the induction step, considering $\check{\nu}_{t+1} = \hat{\nu}_{t+1} + \widehat{\Xi}\left(\check{\nu}_t - \hat{\nu}_t\right) + \widehat{\Lambda}(\check{\pi}-\pi_j) + \widehat{\Gamma}\left(\check{\pi}\check{\nu}_t - \pi_j\hat{\nu}_t\right),\ j=1,2,$ the right-hand side is a continuous function of the data, as the sum and product of a finite number of continuous functions is a continuous function. The same result is valid for $\widehat{\mathrm{TTE}}_{t+1}(\check{\pi},0) = \widehat{\Xi}\widehat{\mathrm{TTE}}_t(\check{\pi},0) + \widehat{\Lambda}\check{\pi} + \widehat{\Gamma}\check{\pi}\check{\nu}_t$ and the induction is complete. Finally, since the composition of continuous functions is continuous, the whole procedure in Steps 2 and 3 of Algorithm 2 is continuous in $\left(\hat{\nu}_0,\ldots,\hat{\nu}_T\right)$, implying the continuity of f.

Step 2. Note that by Theorem 1, there exists a set $\mathcal{C}\subset\Omega$ with $\mathbb{P}(\mathcal{C})=1$ such that $\hat{\nu}_t(\mathcal{E}) = \frac{1}{N}\sum_{n=1}^N Y_t^n$ converges point-wise to $\nu_t(\mathcal{E})$ over $\mathcal{C}$ as $N\to\infty$, where $t\geq 0$ (note that a countable union of zero-measure sets also has measure zero). Henceforth, we focus on the set $\mathcal{C}$ and do all the computations over that. By Eq. (6) and Eq. (4), we get

$$\begin{aligned}&\nu_{t+1}(\mathcal{E}) = \delta + \xi\nu_t(\mathcal{E}) + \lambda\pi_1 + \gamma\nu_t(\mathcal{E})\pi_1 + \vec{\theta}^{\top}\vec{x}, && t=0,\ldots,T_1-1,\\ &\nu_{t+1}(\mathcal{E}) = \delta + \xi\nu_t(\mathcal{E}) + \lambda\pi_2 + \gamma\nu_t(\mathcal{E})\pi_2 + \vec{\theta}^{\top}\vec{x}, && t=T_1,\ldots,T_1+T_2-1.\end{aligned} \quad [98]$$

Above, we utilized the following notations:

$$\delta = \mathbb{E}[\Delta^n], \qquad \xi = \mathbb{E}[\Xi^n], \qquad \lambda = \mathbb{E}[\Lambda^n], \qquad \gamma = \mathbb{E}[\Gamma^n], \qquad \vec{\theta} = \mathbb{E}[\vec{\Theta}^n], \qquad \vec{x} = \mathbb{E}[\boldsymbol{X}],$$

where $\vec{\theta}$ and $\vec{x}$ are the vectors representing the mean of the columns of matrices $\boldsymbol{\Theta}$ and $\boldsymbol{X}$, respectively. Specifically, in Eq. (98), we handle the randomness of $\Delta^n,\Xi^n,\Lambda^n,\Gamma^n$, and $\vec{\Theta}^n$ by extending the matrix $\boldsymbol{X}$; that is, we incorporate these coefficients as components of the individual's covariate vector within an extended matrix $\widetilde{\boldsymbol{X}}$. Note that Assumption 3 holds for the upgraded model, because of the independence and bounded moment assumption of $\Delta^n,\Xi^n,\Lambda^n,\Gamma^n$, and $\vec{\Theta}^n$.

Then, we can rewrite Eq. (98) as follows:

$$\begin{aligned}&\nu_{t+1}(\mathcal{E}) = \left(\xi+\gamma\pi_1\right)\nu_t(\mathcal{E}) + \delta + \lambda\pi_1 + \vec{\theta}^{\top}\vec{x}, && t=0,\ldots,T_1-1,\\ &\nu_{t+1}(\mathcal{E}) = \left(\xi+\gamma\pi_2\right)\nu_t(\mathcal{E}) + \delta + \lambda\pi_2 + \vec{\theta}^{\top}\vec{x}, && t=T_1,\ldots,T_1+T_2-1.\end{aligned} \quad [99]$$

Therefore, regressing $\left(\nu_1(\mathcal{E}),\ldots,\nu_{T_1}(\mathcal{E})\right)^{\top}$ on $\left(\nu_0(\mathcal{E}),\ldots,\nu_{T_1-1}(\mathcal{E})\right)^{\top}$ is exact and we get the coefficient $b_1 = \xi+\gamma\pi_1$ and intercept $a_1 = \delta+\lambda\pi_1+\vec{\theta}^{\top}\vec{x}$. Likewise, we obtain $b_2 = \xi+\gamma\pi_2$ and $a_2 = \delta+\lambda\pi_2+\vec{\theta}^{\top}\vec{x}$. This implies that

$$\widehat{\Xi} = \xi, \qquad \widehat{\Gamma} = \gamma, \qquad \widehat{\Lambda} = \lambda. \quad [100]$$

On the other hand, by Theorem 1 and Eq. (98), for $t=0,\ldots,T_1-1$, we can write

$$\begin{aligned}\nu_{t+1}(\check{\pi}) &= \delta + \xi\nu_t(\check{\pi}) + \lambda\check{\pi} + \gamma\check{\pi}\nu_t(\check{\pi}) + \vec{\theta}^{\top}\vec{x}\\ &= \delta + \xi\nu_t(\mathcal{E}) + \lambda\pi_1 + \gamma\pi_1\nu_t(\mathcal{E}) + \vec{\theta}^{\top}\vec{x} + \xi\left(\nu_t(\check{\pi}) - \nu_t(\mathcal{E})\right) + \gamma\left(\check{\pi}\nu_t(\check{\pi}) - \pi_1\nu_t(\mathcal{E})\right) + \lambda(\check{\pi}-\pi_1)\\ &= \nu_{t+1}(\mathcal{E}) + \xi\left(\nu_t(\check{\pi}) - \nu_t(\mathcal{E})\right) + \gamma\left(\check{\pi}\nu_t(\check{\pi}) - \pi_1\nu_t(\mathcal{E})\right) + \lambda(\check{\pi}-\pi_1),\end{aligned} \quad [101]$$

where we set $\nu_0(\check{\pi}) = \nu_0(\mathcal{E})$. Here, with a slight abuse of notation, we used $\nu_t(\check{\pi})$ since the corresponding experimental design has only one element, which is $\check{\pi}$. Likewise, for $t=T_1,\ldots,T_1+T_2-1$, we can obtain

$$\nu_{t+1}(\check{\pi}) = \nu_{t+1}(\mathcal{E}) + \xi\left(\nu_t(\check{\pi}) - \nu_t(\mathcal{E})\right) + \gamma(\check{\pi}\nu_t(\check{\pi}) - \pi_2\nu_t(\mathcal{E})) + \lambda(\check{\pi}-\pi_2). \quad [102]$$

Also, it is straightforward to check that

$$\begin{aligned}&\nu_{t+1}(0) = \nu_{t+1}(\mathcal{E}) + \xi\left(\nu_t(0) - \nu_t(\mathcal{E})\right) - \gamma\pi_1\nu_t(\mathcal{E}) - \lambda\pi_1, && t=0,\ldots,T_1-1,\\ &\nu_{t+1}(0) = \nu_{t+1}(\mathcal{E}) + \xi\left(\nu_t(0) - \nu_t(\mathcal{E})\right) - \gamma\pi_2\nu_t(\mathcal{E}) - \lambda\pi_2, && t=T_1,\ldots,T_1+T_2-1,\end{aligned} \quad [103]$$

where $\nu_0(0) = \nu_0(\mathcal{E})$.

Considering Eq. (100)-Eq. (103), by the definition of the total treatment effect in Eq. (2) and Theorem 1, over the set $\mathcal{C}$, we have

$$\mathrm{TTE}_{t+1}(\check{\pi},0) = \nu_{t+1}(\check{\pi}) - \nu_{t+1}(0) = \xi\mathrm{TTE}_t(\check{\pi},0) + \gamma\check{\pi}\nu_t(\check{\pi}) + \lambda\check{\pi}. \quad [104]$$

Recalling the fact that $\mathbb{P}(\mathcal{C})=1$ concludes the proof. □

11.6. Proof of Theorem 6: Analysis of the estimator at equilibrium. The proof technique is similar to the proof of Theorem 2. Based on Eq. (104), it is straightforward to obtain the following result:

$$\mathrm{TTE}(1,0) \overset{\text{a.s.}}{=} \frac{\gamma\nu(1)+\lambda}{1-\xi}. \tag{105}$$

But, we have

$$\begin{aligned}\nu(\pi_1) &= (\xi+\gamma\pi_1)\,\nu(\pi_1)+\delta+\lambda\pi_1+\vec{\theta}^\top\vec{x},\\ \nu(\pi_2) &= (\xi+\gamma\pi_2)\,\nu(\pi_2)+\delta+\lambda\pi_2+\vec{\theta}^\top\vec{x}.\end{aligned} \tag{106}$$

On the other hand, by Eq. (31) and Theorem 1, we have

$$\begin{aligned}\lim_{N\to\infty}\widehat{\mathrm{TTE}}(1,0) &= \frac{1}{\pi_2-\pi_1}\lim_{N\to\infty}\sum_{n=1}^{N}\frac{(Y^n(\pi_2)-Y^n(\pi_1))}{N}\\ &\overset{\text{a.s.}}{=} \frac{\nu(\pi_2)-\nu(\pi_1)}{\pi_2-\pi_1}\\ &= \frac{\xi(\nu(\pi_2)-\nu(\pi_1))+\gamma(\pi_2\nu(\pi_2)-\pi_1\nu(\pi_1))}{\pi_2-\pi_1}+\lambda\\ &= \frac{\xi(\nu(\pi_2)-\nu(\pi_1))}{\pi_2-\pi_1}+\frac{\gamma(\pi_2\nu(\pi_2)-\pi_1\nu(\pi_1))}{\pi_2-\pi_1}+\lambda\\ &\overset{\text{a.s.}}{=} \xi\lim_{N\to\infty}\widehat{\mathrm{TTE}}(1,0)+\frac{\gamma(\pi_2\nu(\pi_2)-\pi_1\nu(\pi_1))}{\pi_2-\pi_1}+\lambda,\end{aligned}$$

that implies

$$\lim_{N\to\infty}\widehat{\mathrm{TTE}}(1,0) \overset{\text{a.s.}}{=} \frac{1}{1-\xi}\left(\frac{\gamma(\pi_2\nu(\pi_2)-\pi_1\nu(\pi_1))}{\pi_2-\pi_1}+\lambda\right) \tag{107}$$

Then, by Eq. (105)-Eq. (107), we get

$$\lim_{N\to\infty}\widehat{\mathrm{TTE}}(1,0)-\mathrm{TTE}(1,0) \overset{\text{a.s.}}{=} \frac{\gamma}{1-\xi}\left(\frac{\pi_2\nu(\pi_2)-\pi_1\nu(\pi_1)}{\pi_2-\pi_1}-\nu(1)\right).$$

But, by Theorem 1, we know that $\lim_{N\to\infty}\hat{\nu}(\mathcal{E}) \overset{\text{a.s.}}{=} \nu(\mathcal{E})$, and the proof is complete. □

11.7. Two Versions of Strong Law of Large Numbers. We need the following strong law of large numbers (SLLN) for triangular arrays of independent but not identically distributed random variables. The form stated below is Theorem 3 in (34) that is adapted from Theorem 2.1 in (37).

Theorem 8 (SLLN) *Let $\{X_{n,i}: 1\le i\le n,\ n\ge 1\}$ be a triangular array of random variables such that $(X_{n,1},\ldots,X_{n,n})$ are mutually independent with a mean equal to zero for each n and $\frac{1}{n}\sum_{i=1}^{n}E\left[|X_{n,i}|^{2+\kappa}\right]\le cn^{\kappa/2}$ for some $0<\kappa<1$ and $c<\infty$. Then, we have*

$$\lim_{N\to\infty}\frac{1}{n}\sum_{i=1}^{n}X_{n,i} \overset{\text{a.s.}}{=} 0. \tag{108}$$

We also need the following form of the law of large numbers which is an extension of Lemma 4 in (34).

Theorem 9 *Fix $k\ge 2$ and an integer l and let $\{\boldsymbol{v}(N)\}_{N\ge 1}$ be a sequence of vectors that $\boldsymbol{v}(N)\in\mathbb{R}^{N\times l}$. That means, $\boldsymbol{v}(N)$ is a matrix with N rows and l columns. Assume that the empirical distribution of $\boldsymbol{v}(N)$, denoted by $\hat{p}_N$, converges weakly to a probability measure p_v on $\mathbb{R}^l$ such that $\mathbb{E}_{p_v}\left[\|\vec{V}\|^k\right]<\infty$ and $\mathbb{E}_{\hat{p}_N}\left[\|\vec{V}\|^k\right]\to\mathbb{E}_{p_v}\left[\|\vec{V}\|^k\right]$ as $N\to\infty$. Then, for any continuous function $f:\mathbb{R}^l\mapsto\mathbb{R}$ with at most polynomial growth of order k, we have*

$$\lim_{N\to\infty}\frac{1}{N}\sum_{n=1}^{N}f\big(\boldsymbol{v}_n(N)\big) \overset{\text{a.s.}}{=} \mathbb{E}_{p_v}[f(\vec{V})]. \tag{109}$$

Proof. We use the same truncation technique as Lemma 4 in (34). For a positive integer h, we define

$$f_h(\vec{\omega}) := \begin{cases} h & f(\vec{\omega})>h,\\ f(\vec{\omega}) & |f(\vec{\omega})|\le h,\\ -h & f(\vec{\omega})<h,\end{cases} \tag{110}$$

and write $\tilde{f}_h(\vec{\omega}) := f(\vec{\omega}) - f_h(\vec{\omega})$. Then, by definition of empirical measure, we have

$$\frac{1}{N}\sum_{n=1}^{N} f\big(\boldsymbol{v}_n(N)\big) = \mathbb{E}_{\hat{p}_N}[f(\vec{V})] = \mathbb{E}_{\hat{p}_N}[\tilde{f}_h(\vec{V})] + \mathbb{E}_{\hat{p}_N}[f_h(\vec{V})].$$

Also, we can write

$$\begin{aligned}\liminf_{N\to\infty}\left(\mathbb{E}_{\hat{p}_N}[\tilde{f}_h(\vec{V})] + \mathbb{E}_{\hat{p}_N}[f_h(\vec{V})]\right) &= \liminf_{N\to\infty}\mathbb{E}_{\hat{p}_N}[f(\vec{V})] \\ &\le \limsup_{N\to\infty}\mathbb{E}_{\hat{p}_N}[f(\vec{V})] = \limsup_{N\to\infty}\left(\mathbb{E}_{\hat{p}_N}[\tilde{f}_h(\vec{V})] + \mathbb{E}_{\hat{p}_N}[f_h(\vec{V})]\right).\end{aligned} \qquad [111]$$

On the other hand, because $\hat{p}_N$ converges weakly to p_v, for the bounded continuous function f_h (see, e.g., Section 2 of (38)), we have

$$\lim_{N\to\infty}\mathbb{E}_{\hat{p}_N}[f_h(\vec{V})] = \mathbb{E}_{p_v}[f_h(\vec{V})]. \qquad [112]$$

Considering that f has at most polynomial growth of order k, we can write

$$\left|\tilde{f}_h(\vec{V})\right| \le \left|f(\vec{V})\right| \mathbb{1}_{\{|f(\vec{V})|>h\}} \le c\left(1+\left\|\vec{V}\right\|^k\right)\mathbb{1}_{\{|f(\vec{V})|>h\}} \le c\left(1+\left\|\vec{V}\right\|^k\right)\mathbb{1}_{\left\{\frac{h}{c}-1<\left\|\vec{V}\right\|^k\right\}}. \qquad [113]$$

Putting Eq. (111)-Eq. (113) together, we get

$$\begin{aligned}&\mathbb{E}_{p_v}[f_h(\vec{V})] - \limsup_{N\to\infty}\mathbb{E}_{\hat{p}_N}\left[c\left(1+\left\|\vec{V}\right\|^k\right)\mathbb{1}_{\left\{\frac{h}{c}-1<\left\|\vec{V}\right\|^k\right\}}\right] \\ &\le \liminf_{N\to\infty}\mathbb{E}_{\hat{p}_N}[f(\vec{V})] \\ &\le \limsup_{N\to\infty}\mathbb{E}_{\hat{p}_N}[f(\vec{V})] \\ &\le \mathbb{E}_{p_v}[f_h(\vec{V})] + \limsup_{N\to\infty}\mathbb{E}_{\hat{p}_N}\left[c\left(1+\left\|\vec{V}\right\|^k\right)\mathbb{1}_{\left\{\frac{h}{c}-1<\left\|\vec{V}\right\|^k\right\}}\right].\end{aligned}$$

Now, based on the weak convergence of $\hat{p}_N$ to p_v, we can write

$$\lim_{N\to\infty}\mathbb{E}_{\hat{p}_N}\left[c\left(1+\left\|\vec{V}\right\|^k\right)\mathbb{1}_{\left\{\frac{h}{c}-1\ge\left\|\vec{V}\right\|^k\right\}}\right] = \mathbb{E}_{p_v}\left[c\left(1+\left\|\vec{V}\right\|^k\right)\mathbb{1}_{\left\{\frac{h}{c}-1\ge\left\|\vec{V}\right\|^k\right\}}\right].$$

Recalling the assumption $\lim_{N\to\infty}\mathbb{E}_{\hat{p}_N}\left[\left\|\vec{V}\right\|^k\right] = \mathbb{E}_{p_v}\left[\left\|\vec{V}\right\|^k\right]$, this implies that

$$\begin{aligned}\limsup_{N\to\infty}\mathbb{E}_{\hat{p}_N}\left[c\left(1+\left\|\vec{V}\right\|^k\right)\mathbb{1}_{\left\{\frac{h}{c}-1<\left\|\vec{V}\right\|^k\right\}}\right] &= \lim_{N\to\infty}\mathbb{E}_{\hat{p}_N}\left[c\left(1+\left\|\vec{V}\right\|^k\right)\mathbb{1}_{\left\{\frac{h}{c}-1<\left\|\vec{V}\right\|^k\right\}}\right] \\ &= \mathbb{E}_{p_v}\left[c\left(1+\left\|\vec{V}\right\|^k\right)\mathbb{1}_{\left\{\frac{h}{c}-1<\left\|\vec{V}\right\|^k\right\}}\right].\end{aligned}$$

But, applying the dominated convergence theorem (e.g., Theorem 16.4 in (35)), we get

$$\lim_{h\to\infty}\mathbb{E}_{p_v}\left[c\left(1+\left\|\vec{V}\right\|^k\right)\mathbb{1}_{\left\{\frac{h}{c}-1<\left\|\vec{V}\right\|^k\right\}}\right] = 0,$$

where we used $\mathbb{E}_{p_v}\left[\left\|\vec{V}\right\|^k\right] < \infty$. To conclude the proof, note that $\mathbb{E}_{p_v}[f_h(\vec{V})] \to \mathbb{E}_{p_v}[f(\vec{V})]$ as $h \to \infty$ by reusing the dominated convergence theorem. □

1. F Sävje, P Aronow, M Hudgens, Average treatment effects in the presence of unknown interference. *Annals statistics* **49**, 673 (2021).
2. S Li, S Wager, Random graph asymptotics for treatment effect estimation under network interference. *The Annals Stat.* **50**, 2334–2358 (2022).
3. I Goodfellow, Y Bengio, A Courville, *Deep learning.* (MIT press), (2016).
4. A Rahimi, B Recht, Random features for large-scale kernel machines in *Advances in Neural Information Processing Systems*, eds. J Platt, D Koller, Y Singer, S Roweis. (Curran Associates, Inc.), Vol. 20, (2007).
5. D Eckles, B Karrer, J Ugander, Design and analysis of experiments in networks: Reducing bias from interference. *J. Causal Inference* **5**, 20150021 (2016).
6. DL Sussman, EM Airoldi, Elements of estimation theory for causal effects in the presence of network interference. *arXiv preprint arXiv:1702.03578* (2017).
7. MP Leung, Treatment and spillover effects under network interference. *Rev. Econ. Stat.* **102**, 368–380 (2020).
8. D Viviano, Experimental design under network interference. *arXiv preprint arXiv:2003.08421* (2020).
9. A Agarwal, S Cen, D Shah, CL Yu, Network synthetic interventions: A framework for panel data with network interference. *arXiv preprint arXiv:2210.11355* (2022).
10. A Belloni, F Fang, A Volfovsky, Neighborhood adaptive estimators for causal inference under network interference. *arXiv preprint arXiv:2212.03683* (2022).
11. M Cortez, M Eichhorn, C Yu, Staggered rollout designs enable causal inference under interference without network knowledge in *Advances in Neural Information Processing Systems.* (2022).
12. M Cortez, M Eichhorn, CL Yu, Exploiting neighborhood interference with low order interactions under unit randomized design. *arXiv preprint arXiv:2208.05553* (2022).
13. CL Yu, EM Airoldi, C Borgs, JT Chayes, Estimating the total treatment effect in randomized experiments with unknown network structure. *Proc. Natl. Acad. Sci.* **119**, e2208975119 (2022).
14. MP Leung, Causal inference under approximate neighborhood interference. *Econometrica* **90**, 267–293 (2022).
15. Y Jiang, H Wang, Causal inference under network interference using a mixture of randomized experiments. *arXiv preprint arXiv:2309.00141* (2023).
16. S Li, S Wager, Network interference in micro-randomized trials. *arXiv preprint arXiv:2202.05356* (2022).
17. Z Bai, J Silverstein, *Spectral Analysis of Large Dimensional Random Matrices.* (Springer), (2005).
18. GW Anderson, A Guionnet, O Zeitouni, *An introduction to random matrices.* (Cambridge University Press), (2009).
19. T Tao, V Vu, Random matrices: The Universality phenomenon for Wigner ensembles. arXiv:1202.0068 (2012).
20. M Bayati, M Lelarge, A Montanari, Universality in polytope phase transitions and message passing algorithms. *The Annals Appl. Probab.* **25**, 753 – 822 (2015).
21. A Montanari, R Venkataramanan, Estimation of low-rank matrices via approximate message passing. *The Annals Stat.* **49**, 321 – 345 (2021).
22. R Berthier, A Montanari, PM Nguyen, State evolution for approximate message passing with non-separable functions. *Inf. Inference: A J. IMA* **9**, 33–79 (2020).
23. WK Chen, WK Lam, Universality of approximate message passing algorithms. *arXiv preprint arXiv:2003.10431* (2020).
24. X Zhong, T Wang, Z Fan, Approximate Message Passing for orthogonally invariant ensembles: Multivariate non-linearities and spectral initialization. *arXiv e-prints* (2021).
25. R Dudeja, YM Lu, S Sen, Universality of approximate message passing with semirandom matrices. *The Annals Probab.* **51**, 1616 – 1683 (2023).
26. T Wang, X Zhong, Z Fan, Universality of Approximate Message Passing algorithms and tensor networks. *arXiv e-prints* p. arXiv:2206.13037 (2022).
27. F Krzakala, M Mézard, F Sausset, Y Sun, L Zdeborová, Statistical-physics-based reconstruction in compressed sensing. *Phys. Rev. X* **2**, 021005 (2012).
28. D Donoho, A Javanmard, A Montanari, Information-theoretically optimal compressed sensing via spatial coupling and approximate message passing. *Inf. Theory, IEEE Transactions on* **59**, 7434–7464 (2013).
29. A Javanmard, A Montanari, State evolution for general approximate message passing algorithms, with applications to spatial coupling. *Inf. Inference: A J. IMA* **2**, 115–144 (2013).
30. S Athey, D Eckles, GW Imbens, Exact p-values for network interference. *J. Am. Stat. Assoc.* **113**, 230–240 (2018).
31. L Forastiere, F Mealli, A Wu, EM Airoldi, Estimating causal effects under network interference with bayesian generalized propensity scores. *J. Mach. Learn. Res.* **23**, 1–61 (2022).
32. R Xiong, S Athey, M Bayati, G Imbens, Optimal experimental design for staggered rollouts. *arXiv preprint arXiv:1911.03764* (2019).
33. K Imai, Z Jiang, , et al., Identification and sensitivity analysis of contagion effects in randomized placebo-controlled trials. *JR Stat Soc Ser A Stat Soc* **11** (2019).
34. M Bayati, A Montanari, The dynamics of message passing on dense graphs, with applications to compressed sensing. *IEEE Transactions on Inf. Theory* **57**, 764–785 (2011).
35. P Billingsley, *Probability and measure.* (John Wiley & Sons), (2008).
36. AW Van der Vaart, *Asymptotic statistics.* (Cambridge university press) Vol. 3, (2000).
37. TC Hu, R Taylor, On the strong law for arrays and for the bootstrap mean and variance. *Int. J. Math. Math. Sci.* **20**, 375–382 (1997).
38. P Billingsley, *Convergence of probability measures.* (John Wiley & Sons), (2013).
